# Converging *ab initio* phonon simulations for organic molecular crystals: the effect of charge density grids and phonon dispersion sampling

**DOI:** 10.1039/d5ce01090j

**Published:** 2025-12-03

**Authors:** Mateusz Mojsak, Tahlia M. Palmer, Adam A. L. Michalchuk

**Affiliations:** a School of Chemistry, University of Birmingham Edgbaston Birmingham B15 2TT UK a.a.l.michalchuk@bham.ac.uk; b Federal Institute for Materials Research and Testing (BAM) Wilhelm-Ostwald-Strasse 18 12489 Berlin Germany

## Abstract

We here explore how some frequently overlooked computational parameters affect the simulation of phonon frequencies in organic molecular crystals within the framework of density functional perturbation theory in a pseudo-core plane wave basis set. Specifically, we investigate how the density of the Fourier grid that is used to map real-space charge density affects the phonon frequencies and eigenvectors. We find that varying the density of this Fourier grid can affect low-frequency phonons by tens of wavenumbers and significantly alter the associated normal mode eigenvectors. Furthermore, we demonstrate that poorly converged charge density representations can lead to substantial errors in simulated thermodynamic quantities, with vibrational free energies affected by 3–4 kJ mol^−1^ in certain systems. We show how this variation in predicted free energies can have a significant impact on our ability to correctly predict the relative stability of a series of model polymorphic systems. We finally discuss how careful convergence with respect to the Brillouin zone (*q*-point) sampling is imperative for the correct modelling of phonon dispersion relations in organic molecular crystals, particularly for systems characterised by weak, anisotropic interactions. Whilst no definitive ‘rules of thumb’ emerge for the convergence of these parameters, our findings highlight the critical role they play in obtaining reliable phonon frequencies from density functional perturbation theory. Our results also offer insight into the potential magnitude of errors that could arise in phonon simulations of organic molecular crystals if these parameters are not chosen carefully.

## Abstract 2

The quality of charge density representations in linear response phonon calculations for organic molecular crystals significantly impacts the predicted thermodynamic properties.

## Graphical abstract

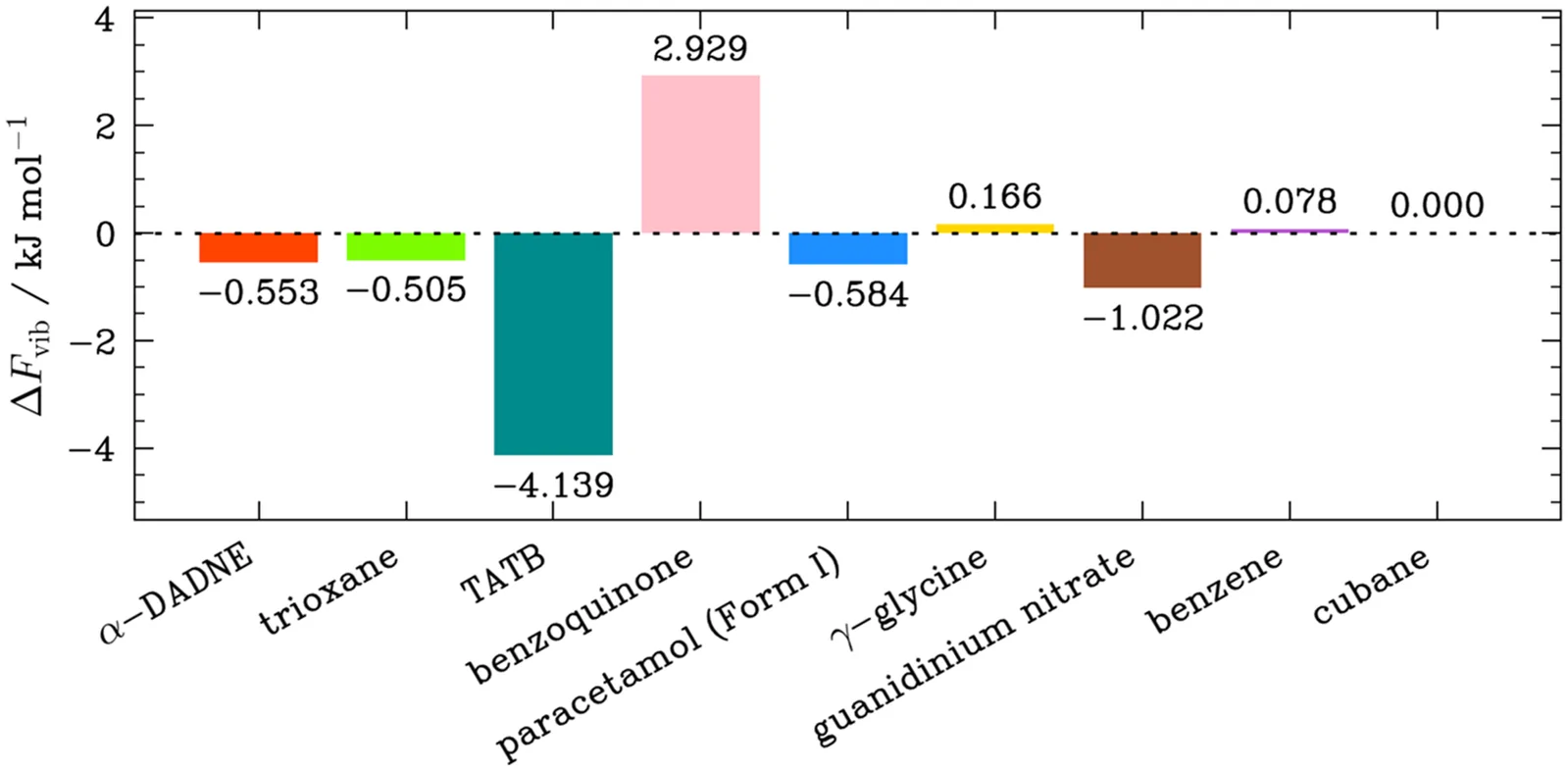

## 1 Introduction

Organic molecular crystals (OMCs) have wide-ranging technological applications, from pharmaceuticals^1^ and high-energy-density materials,^2^ to components in micro-electronic,^3^ micro-optical,^4^ piezoelectric energy-harvesting,^5^ and mechanically actuating devices.^6^ The ability to design, and ultimately optimise, the performance of OMCs for these various technologies requires a clear link between the desired functional behaviour and the underlying molecular and crystal structure. Our typical understanding of ‘structure’ is rooted in representations of static nuclear geometries.^7^ These representations are, of course, extremely useful and underpin most of the last century's developments in the chemical sciences.

As research targets increasingly complex physical phenomena and requires a more detailed characterisation of crystal behaviour, traditional models based on static nuclei are no longer sufficient. For example, to correctly describe the crystal structure landscapes associated with crystal form (polymorph) screening, we must consider the vibrational contributions to the free energy,^8^ which are essential to resolve the subtle energy differences between polymorphs. Similarly, the thermal response of OMCs is also governed by the vibrational behaviour of molecules within the crystalline structure.^9,10^ In fact, even seemingly ‘simple’ properties of OMCs, which are often studied by using static-nuclei models, can be affected by vibrational dynamics. For example, a marked renormalisation of electronic band gaps has been observed in molecular crystals once nuclear motion is taken into account.^11^ Beyond the intrinsic physical properties of crystals, the dynamics of OMCs are central to understanding the chemical reactivity of molecules in the solid state. Mechanisms for the mechanochemical initiation of explosives, for example, have been linked to the channelling of kinetic energy through the vibrational modes of OMCs.^12,13^ In reality, most functional properties of OMCs depend on crystal dynamics to some (sometimes subtle) degree. Hence, accurate theoretical models of this behaviour are essential for studying the intricate link between crystal dynamics and OMC properties.

Given the increasingly recognised importance of vibrational dynamics in the study of OMCs, it is not surprising that recent years have seen a significant increase in efforts to simulate this behaviour. Within quantum chemical codes, there are two conventional ways of calculating vibrational dynamics: (1) the ‘brute force’ *finite difference* method,^14^ and (2) the perturbative *linear response* method.^15^ In the former, the force constant matrix is constructed explicitly by computing the forces associated with small atomic displacements in a model crystal structure. This method is relatively straightforward to implement, and is compatible with any energy calculator (including *via* classical potentials), which has made it particularly popular for modelling phonons. However, because the force constant matrix is calculated explicitly, one can only obtain dynamical matrices (*i.e.* reciprocal space representations of the force constants) at wavevectors that are commensurate with the number of primitive representations in the supercell that is used in the model. Correspondingly, to generate accurate phonon dispersion curves, large supercells are typically required, quickly rendering the method computationally intractable for large, low-symmetry OMCs. Reducing the cost of finite difference methods for OMC phonon simulations using various approximations or lower-cost force calculators has been the subject of active research.^16,17^ In contrast, the linear response method obtains the force constants through a first-order (linear) perturbation of the calculated charge density, and is therefore only compatible with quantum chemical methods. However, the phase of the perturbation can be arbitrarily chosen and hence dynamical matrices at any wavevector are directly available from the primitive unit cell. Therefore, this method is often preferred for simulating the phonon spectra of OMCs.

‘Rules of thumb’ have been explored as strategies for achieving convergence of various calculated properties of OMCs, for example with respect to electronic structure sampling.^18^ However, apart from efforts to determine how different levels of theory (*e.g.*, density functionals, force fields, *etc.*) affect internuclear force constants and hence phonon frequencies,^17,19^ little work is available for understanding how calculation parameters themselves can influence the convergence of phonon spectra in OMCs using linear response methods. Given the rapidly growing community of both modellers and experimentalists interested in accessing phonon properties through computational methods, we feel that some discussion of the convergence behaviour, especially of less commonly discussed computational parameters, is timely. Such an investigation is therefore the aim of the present manuscript. We note that it is not our intention to provide a ‘one-size-fits-all’ process to converge phonon frequency calculations, but rather to demonstrate the influence that some less obvious simulation parameters can have on phonon frequencies and common related properties. It is our hope that the present work will provide food-for-thought guidance for those interested in simulating phonon spectra for OMCs, and will help consolidate strategies for obtaining reliable and reproducible phonon data for OMC property prediction.

## 2 Computational methods

### 2.1 Input crystal structures

All structures used in this manuscript were obtained from the Inorganic Crystal Structure Database (ICSD)—1,1-diamino-2,2-dinitroethylene (α-polymorph code: 172530, β-polymorph code: 172534)^20^—or the Cambridge Structural Database: trioxane (REF: TROXAN11),^21^ 1,3,5-triamino-2,4,6-trinitrobenzene (REF: TATNBZ03),^22^ paracetamol form I (REF: HXACAN36),^23^ paracetamol form II (REF: HXACAN08),^24^ γ-glycine (REF: GLYCIN15),^25^ α-glycine (REF: GLYCIN04),^26^ guanidinium nitrate (REF: LETGIA),^27^ benzoquinone (REF: BNZQUI03),^28^ benzene (REF: BENZEN20),^29^ and cubane (REF: CUBANE01).^30^

### 2.2 Structure relaxation

Experimental structures were relaxed within the framework of plane-wave density functional theory (DFT) as implemented in CASTEP v22.11.^31^ The Hamiltonian was approximated with the generalised gradient approximation (GGA) functional of Perdew–Burke–Ernzerhof (PBE)^32^ with the addition of the Grimme D2 dispersion correction.^33^ This method has been shown to yield good agreement between calculated and experimental inelastic neutron scattering spectra for CHNO molecular crystals,^34^ and hence is a reliable approach for modelling phonon spectra in this class of crystals. The nuclear Coulomb potential was attenuated with norm-conserving pseudopotentials as obtained on-the-fly within the CASTEP suite. The wavefunction was expanded in a plane-wave basis set to a kinetic energy cut-off of 1200 eV and sampled on a Monkhorst–Pack *k*-point grid with 0.05 Å^−1^ spacing; these parameters were found to be more than sufficient to obtain well-converged total electronic energies and forces for all the model systems. The convergence criteria for the self-consistent field cycles were set for the electronic energy change at <1 × 10^−10^ eV and for the electronic eigenvalue change at <1 × 10^−12^ eV. The Broyden density-mixing scheme was used to accelerate the convergence of the SCF calculation, with a mixing amplitude of 0.5 and a maximum mixing *G*-vector of 1.5 Å^−1^. Structure relaxation was performed using the limited-memory Broyden–Fletcher–Goldfarb–Shanno (LBFGS) algorithm^35^ and convergence was accepted once the total energy change reached <2 × 10^−6^ eV per atom, residual forces <1 × 10^−4^ eV Å^−1^, ionic displacements <1 × 10^−5^ Å and cell strain <0.1 GPa.

### 2.3 Phonon calculations

All phonon calculations were performed within the linear response method, as implemented in CASTEP v22.11.^31^ In the plane wave linear response formalism, we can define the forces on atom *I* at position **R**_*I*_ as^36^1
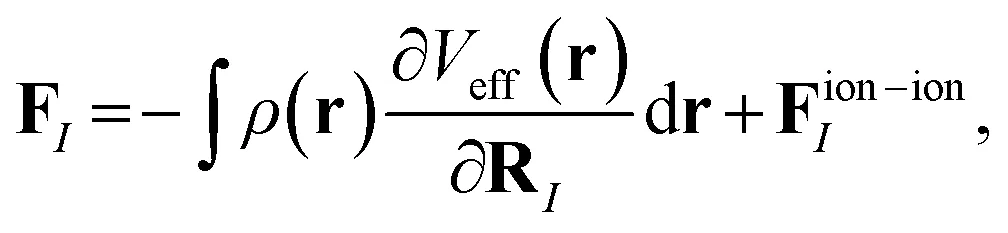
where, *V*_eff_ is the effective potential exerted on the nucleus from the electron density distribution, **F**^ion–ion^_*I*_ describes the force associated with the inter-nuclear interactions, and 2
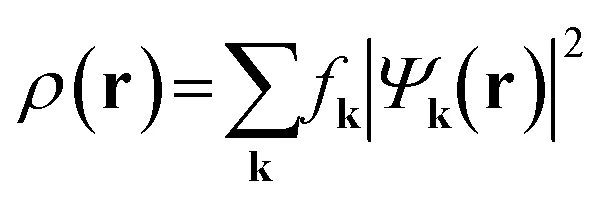
is the electron density constructed from wavefunctions *Ψ*_**k**_(**r**) evaluated at selected wavevectors **k** in the first Brillouin zone. These wavefunctions are expanded as a Fourier series,3
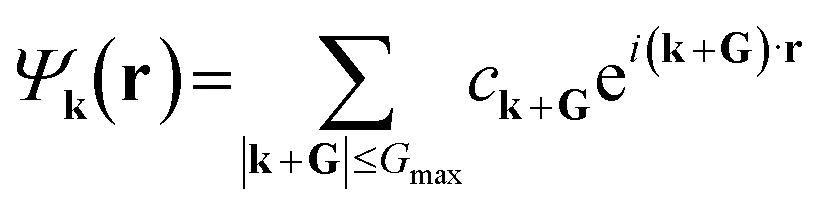
with *c*_**k**+**G**_ representing the coefficients associated with plane wave contributions to *Ψ*_**k**_(**r**). The maximum frequency plane wave included in the expansion, defined by the cut-off energy for the basis set *E*_cut_, is limited by 
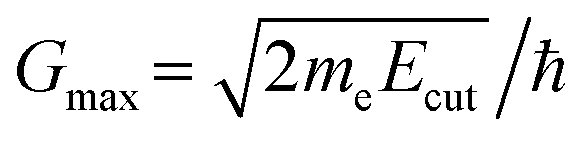
. Since *ρ*(**r**) is obtained as the square of the wavefunction, its exact representation contains components up to twice the *G*_max_ used in the expansion of the wavefunction. Hence, the real-space representation of *ρ*(**r**) is defined by a Fourier grid with up to double the point density as compared to the wavefunction grid. In CASTEP, the density of the *ρ*(**r**) Fourier grid (the ‘standard grid’) is characterised by *G*^std^_max_, where the grid scale relative to the wavefunction grid can be modified by the user. In other solid-state DFT codes, *G*^std^_max_ is either determined automatically from the user-specified *E*_cut_ (Vienna *ab initio* simulation package,^37^ VASP) or calculated from a separate kinetic energy cut-off (ABINIT,^38^ QuantumESPRESSO^39^).

The charge density stored on a grid defined by *G*^std^_max_ = 2*G*_max_ is exact, and hence provides an exact energy. However, the finite basis set size means it inherently omits the higher-frequency wavefunction components that correspond to the atomic core regions when the wavefunction is pseudised, the representation of which would require a finer sampling of *ρ*(**r**). In practice, this can lead to numerical errors when evaluating quantities that depend on the charge density and its spatial derivatives, such as the forces in eqn (1) and, correspondingly, the quality of calculated phonon frequencies. To mitigate this issue, many codes employ a finer grid scale, typically constructed by adding additional *G*-vectors beyond the formal (2*G*_max_) limit required to represent the charge density. In CASTEP, the scale of this ‘fine’ grid relative to *G*_max_ defined by the basis set cut-off energy can be directly set by the user, resulting in the maximum Fourier component *G*^fine^_max_ = fine grid scale × *G*_max_.

In the present work, we performed linear response phonon calculations using a standard grid defined by *G*^std^_max_ = 2*G*_max_ (corresponding to 35.4943 Å^−1^ at the selected kinetic energy cut-off of 1200 eV), varying the quality of the fine grid, as described in Table 1. Since the numerical evaluation of the atomic forces depends critically on the sampling of *ρ*(**r**), this allows us to investigate the impact of the fine grid scale on the phonon frequencies and eigenvectors calculated for OMCs.

**Table 1:** Quality of the fine Fourier grid used in the phonon calculations, defined by scale of the fine grid *G*^fine^_max_ relative to the basis set *G*_max_ = 17.7472 Å^−1^ at the selected *E*_cut_ = 1200 eV

Label	Fine grid scale	Size of *G*^fine^_max_/Å^−1^
I	2.0	35.4943
II	3.0	53.2415
III	4.0	70.9887
IV	5.0	88.7359
V	6.0	106.4830
VI	7.0	124.2302

To calculate the phonon dispersion relations for our OMCs, their respective Brillouin zones were sampled on *Γ*-centred Monkhorst–Pack grids with increasingly finer *q*-point spacing, as discussed in the main text. The phonon frequencies were interpolated from these explicitly calculated grids onto a path between high-symmetry Brillouin zone points, as generated by the SeeKpath utility.^40,41^ To study how phonon mode frequencies converge across the dispersion relations, the calculated frequencies were re-ordered at each *q*-point in order to maximise the magnitude of the scalar product between pairs of eigenvectors that were calculated using different sampling grids. This procedure ensured that our comparisons were made against equivalent eigenvectors (unless otherwise stated in the text).

Vibrational free energies were obtained by performing a standard thermodynamic calculation in CASTEP. Local mode force constants were obtained using an in-house modified version of the solid-state implementation of the LModeA software,^42,43^ capable of an automated analysis^44^ of dynamical matrices obtained from *Γ*-point phonon calculations in CASTEP.

### 2.4 Simulated inelastic neutron scattering spectra

Experimental inelastic neutron scattering (INS) spectra were obtained from the ISIS INS database for α-1,1-diamino-2,2-dinitroethylene,^45^ trioxane,^46^ 1,3,5-triamino-2,4,6-trinitro-benzene,^34^ γ-glycine,^47^ α-glycine,^47^ and benzene.^48^ We compared this experimental data to simulated INS spectra obtained using the Mantid 6.8.0 software,^49,50^ computed without consideration of higher order quantum events (section S2). All simulations were performed using a temperature of 20 K to best reflect the experimental conditions against which we are comparing.

## 3 Results and discussion

It is our primary aim to gauge how the construction of the Fourier grid that is used for the real-space charge density (*G*^fine^_max_) affects the behaviour of the phonon frequencies and eigenvectors obtained through linear response theory. Our study assumes that the other convergence criteria are well converged. In this respect, we have not performed a complete analysis of how density functionals or dispersion corrections affect the accuracy of the frequencies, though some such analyses are available elsewhere,^17,19^ and a brief discussion is given with respect to INS spectra in SI section S2. Moreover, we note that the exact convergence behaviour will depend on the specific pseudopotential construction that one uses. However, we anticipate that our analyses will serve as a useful guide when performing and assessing the reliability of phonon calculations of organic molecular crystals (OMCs).

**Fig. 1:**
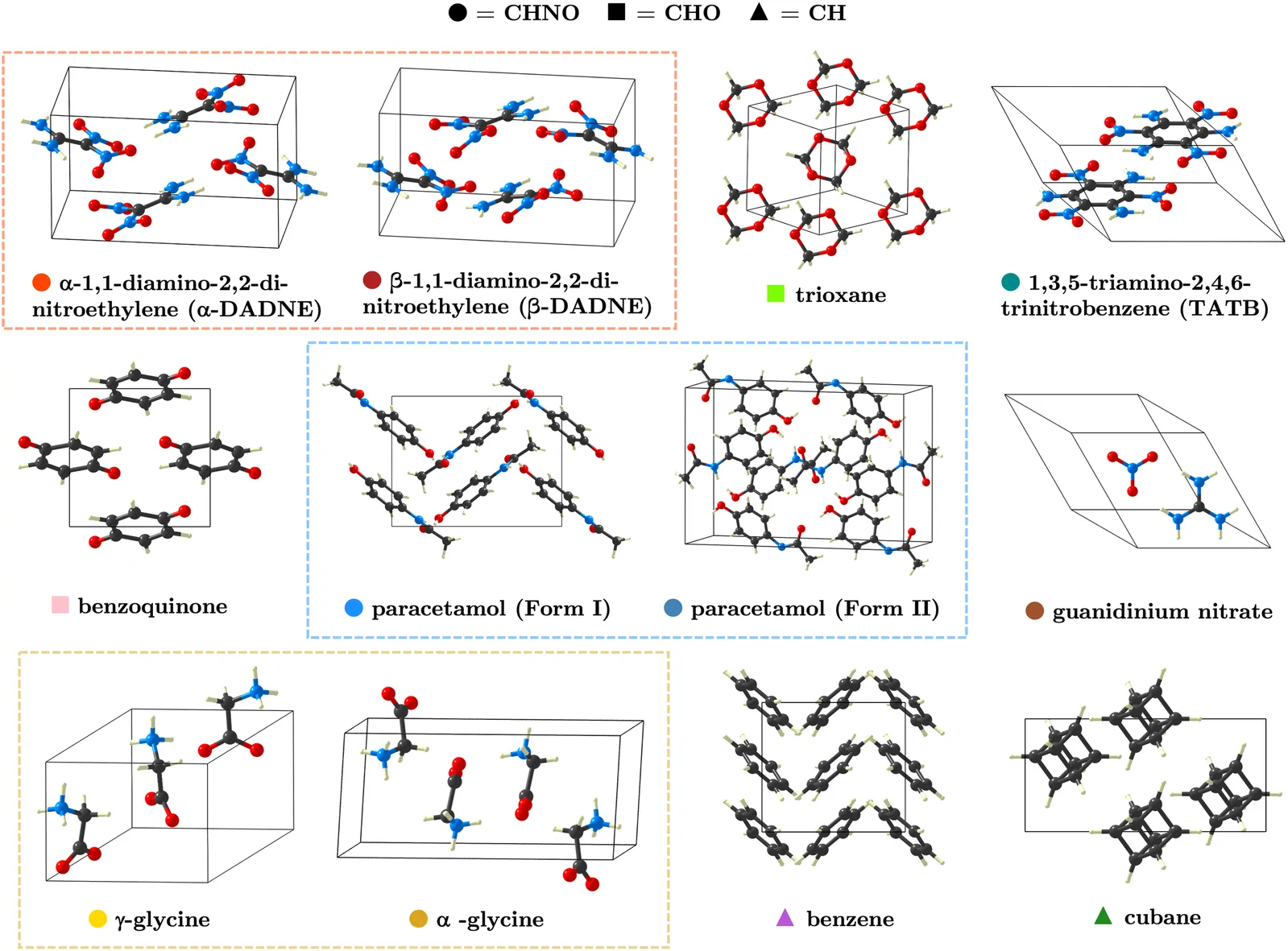
Crystal packing diagrams for the eight OMCs studied in this work. In each case, atoms are coloured as oxygen (red), nitrogen (blue), carbon (black), and hydrogen (white). The unit cells are shown as black wires in each case. The coloured shapes alongside OMC names are used throughout the text to identify them, and correspond to CHNO (circle), CHO (square), and CH (triangle) systems. Pairs of polymorphic forms are shown in coloured boxes for DADNE, paracetamol and glycine.

As model systems for our study, we selected a series of CHNO compounds that reflect common types of bonding in OMCs, Fig. 1. This includes a dispersion-bound crystal (cubane), π-stacked systems (benzene and 1,3,5-triamino-2,4,6-trinitrobenzene (TATB)), examples of crystals with intra-(TATB) and inter-(polymorphs of 1,1-diamino-2,2-dinitroethylene (DADNE), glycine, and paracetamol) molecular hydrogen bonding, and charged systems (glycine and guanidinium nitrate). Within the study, we have also ensured the inclusion of both small, rigid molecules and large, flexible molecules, thereby allowing us to assess how both external and soft internal vibrations respond to the increasing sampling of the charge density.

### 3.1 Phonon convergence at the Brillouin zone centre

Prior to performing phonon calculations, we relaxed all crystal geometries at the PBE-D2 level of theory, subject to the convergence criteria noted in Section 2.2, using a plane wave basis set expanded to 1200 eV (*G*_max_ = 17.7472 Å^−1^). For all cases, we used a standard Fourier grid defined by *G*^std^_max_ = 2*G*_max_ = 35.4943 Å^−1^, and varied the fine grid *G*^fine^_max_ as described in Table 1. Using these parameters, the unit cells simulated for each system reproduced the low temperature experimental geometries well, SI section S1. We note that increasing *G*^fine^_max_ had a negligible effect on the crystal geometries; any change in the phonon frequencies is purely the result of changes in numerical accuracy of the real-space charge densities. For the optimised OMC geometries obtained with the highest fine grid scale (*G*^fine^_max_ = 124.2302 Å^−1^, VI in Table 1), we compared simulated inelastic neutron scattering (INS) spectra to experimental spectra available in the ISIS INS Database. This comparison of INS spectra showed good agreement, thereby validating the models used in this study, see SI section S2.

**Fig. 2:**
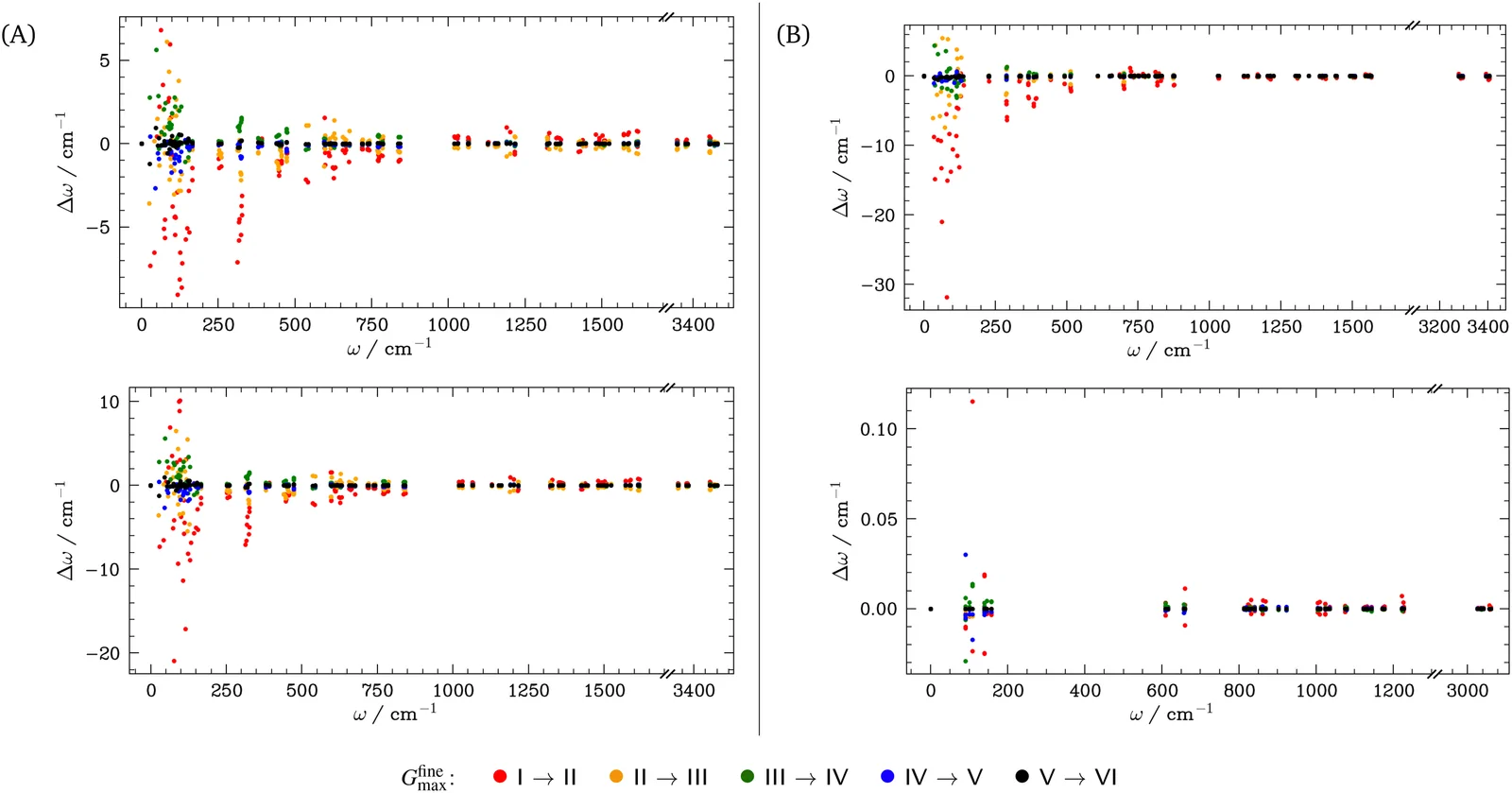
Convergence behaviour of *Γ*-point phonon frequencies for OMCs with increasing *G*^fine^_max_. (A) Convergence behaviour of α-DADNE (top) based on a frequency-ordered list and (bottom) using a re-ordered list according to eigenvector. (B) Convergence behaviour of (top) intramolecular hydrogen-bonded crystal TATB and (bottom) dispersion-bound crystal cubane, both using eigenvector re-ordered lists. Plots for all model OMCs are given in SI section S3.

We next explore the convergence of Brillouin zone-centre frequencies by calculating the change in frequency, Δ*ω*, as *G*^fine^_max_ is varied according to Table 1. We compare the frequencies (1) directly in ascending order, as conventionally reported by simulation codes, Fig. 2A (top) and SI section S3.1, and (2) re-ordered to maximise the magnitude of the scalar product between pairs of eigenvectors, to ensure that comparison is made between equivalent normal modes, Fig. 2A (bottom), Fig. 2B and SI section S3.1. As a general observation, we find that only the low-frequency phonon modes are significantly affected by the change in *G*^fine^_max_, with negligible effect seen above *ca.* 400 cm^−1^ in all cases. In fact, above *ω* ≈ 400 cm^−1^, the frequencies change by <1 cm^−1^ as *G*^fine^_max_ is increased. This is consistent with the relation *ω* ∝ 
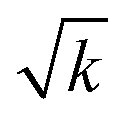
, where *k* is an effective force constant. Within linear response theory, *k* depends on the response of the charge density *ρ*(**r**) to an infinitesimal perturbation *λ*, *k* ∝ ∂*ρ*(**r**)/∂*λ*. Consequently, lower frequency phonons, characterised by lower magnitude force constants, are more susceptible to numerical noise in *ρ*(**r**).

As a representative example, we consider the hydrogen-bonded crystal α-DADNE. When *ω* are compared in ascending order, an increase in *G*^fine^_max_ from setting I (*G*^fine^_max_ = *G*^*s*td^_max_ = 2*G*_max_) to setting II (*G*^fine^_max_ = 3*G*_max_) yields Δ*ω* with magnitudes up to ±10 cm^−1^ for the low frequency modes, Fig. 2A (top). The magnitude of Δ*ω* decreases with a continued increase in *G*^fine^_max_, though frequency variations of >5 cm^−1^ are still observed up to grid setting IV (*G*^fine^_max_ = 5*G*_max_). The effect that changing *G*^fine^_max_ has on the phonon frequencies is magnified when *ω* are re-ordered according to their corresponding eigenvectors, Fig. 2A (bottom), with a change in *G*^fine^_max_ from settingI to II yielding Δ*ω* > 20 cm^−1^ in some cases. However, this variation drops again to *ca.* 2–3 cm^−1^ once a grid setting of IV is reached.

Fig. 2B compares the influence that *G*^fine^_max_ has on the phonon frequencies in two different molecular crystals: TATB, which is hydrogen-bonded and contains CHNO ions, and cubane, which is composed only of CH and is stabilised by dispersion forces. As in the case of α-DADNE, Fig. 2A, the lowest phonon frequencies in TATB are highly sensitive to changes in *G*^fine^_max_. When normal modes are reordered by their eigenvectors, we observe Δ*ω* of up to *ca.* 30 cm^−1^ as the grid setting increases from I to II. However, convergence within 2–3 cm^−1^ is achieved once grid setting IV is used. In contrast, the phonon frequencies of cubane remain virtually unchanged across all grid settings, indicating minimal sensitivity to *G*^fine^_max_. This stark difference between the sensitivity of hydrogen-bonded, CHNO-containing systems like DADNE and TATB, and the insensitivity of dispersion-bound CH-containing systems like cubane (and benzene, SI section S3) raises an important question: is this sensitivity of *ω* to *G*^fine^_max_ driven only by the specific ions that are involved, or is it also influenced by the nature of the intermolecular interactions?

**Fig. 3:**
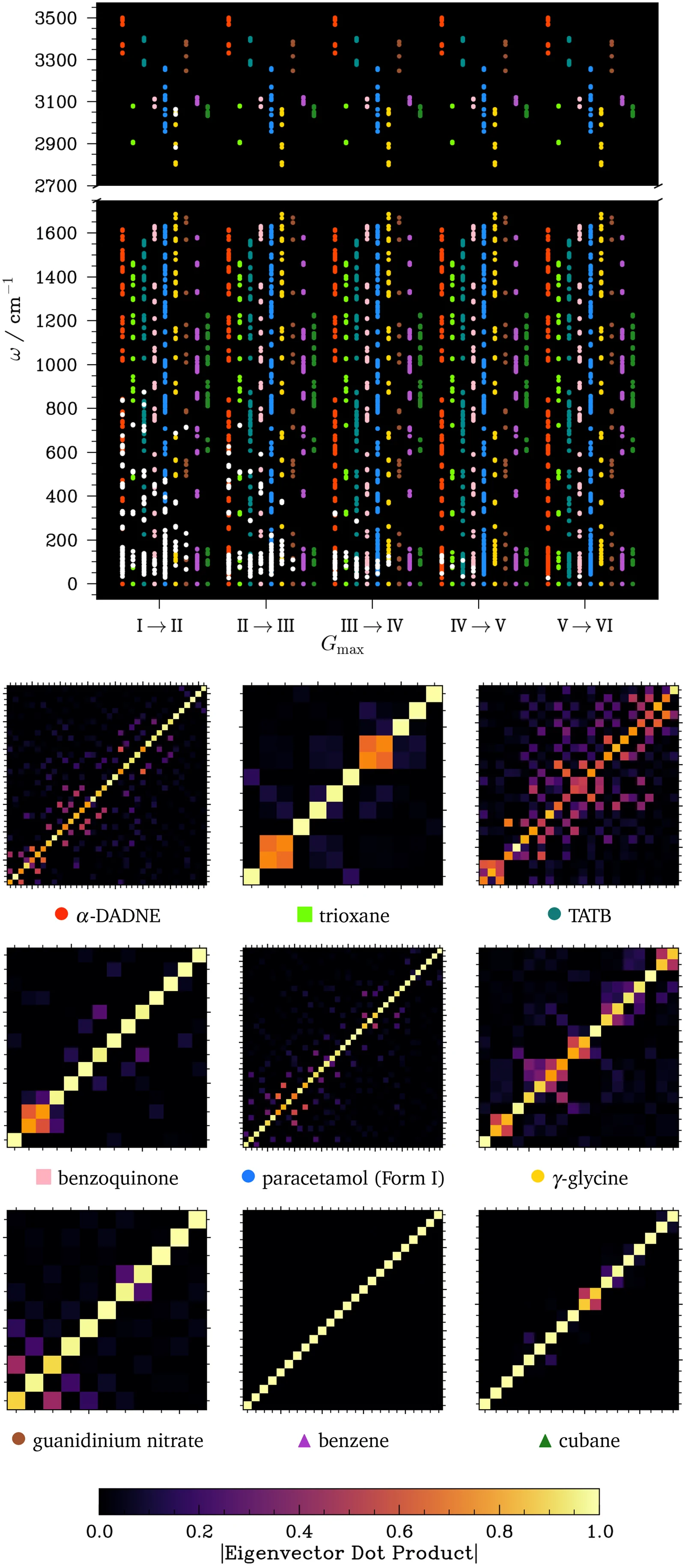
Effect of *G*^fine^_max_ on the convergence behaviour of phonon simulations for model OMCs. (Top) Summary of convergence behaviour for the OMCs studied in this work, where convergence is considered when Δ*ω* < 1 cm^−1^ as *G*^fine^_max_ is increased according to the values in Table 1. Unconverged frequencies are shown in white, with converged frequencies displayed in colour. (Bottom) Absolute dot product matrix between phonon eigenvectors calculated at the fine grid setting I (vertical) and fine grid setting VI (horizontal), shown for all phonon modes with *ω* < 200 cm^−1^. Tick marks represent mode indices, counting from the bottom-left of each matrix plot.

To delve into this question, we have identified which value of *G*^fine^_max_ is needed to converge the phonon frequencies in our eight test OMCs to Δ*ω* < 1 cm^−1^, Fig. 3 (top). Equivalent convergence behaviour analyses are shown in SI section S3.2, using convergence thresholds of 0.5, 2 and 5 cm^−1^. At the outset, we anticipated that higher values of *G*^fine^_max_ would be needed to converge phonon frequencies for systems that contain harder pseudised cores (here, O ions have the hardest cores). Indeed, we do find that the phonon frequencies for our CH-containing systems (cubane and benzene) are fully converged to within our 1 cm^−1^ threshold, even with grid setting I. In contrast, much higher *G*^fine^_max_ are needed to converge the phonon frequencies for CHO (benzoquinone, trioxane) and CHNO (glycine, paracetamol, TATB and DADNE) systems. Of these six OMCs, a grid setting of IV is needed to converge CHNO systems γ-glycine and paracetamol, while a setting of V is needed to achieve convergence for the remaining four systems, albeit with α-DADNE still showing one unconverged low-frequency (*ca.* 27 cm^−1^) mode. We do not see any obvious correlation between the nature of intermolecular bonding and the phonon convergence behaviour (note CHO systems that are primarily dispersion-bound also require high *G*^fine^_max_ to achieve convergence), beyond the general observation that the lowest frequency vibrations are consistently the last to achieve convergence. Moreover, as it is equally challenging to achieve convergence for the rigid molecules trioxane and benzoquinone as for the more flexible molecules DADNE and paracetamol, our findings suggest molecular flexibility is also not a good indicator for the ease of phonon frequency convergence.

The change in *ω* with *G*^fine^_max_ indicates a change in the strength of the interatomic forces derived from variations in the charge density. Unsurprisingly, we also find that notable changes can arise in the phonon eigenvectors associated with modes of *ω* < 200 cm^−1^, Fig. 3 (bottom). This sensitivity of the phonon eigenvectors to *G*^fine^_max_ is particularly pronounced for the more strongly-bonded OMCs, DADNE, TATB, glycine, and paracetamol. In contrast, weakly bonded crystals cubane, benzoquinone, and trioxane contain only a small number of eigenvectors (two, two, and four, respectively) that are strongly affected by *G*^fine^_max_. We do not find that any of the phonon eigenvectors in benzene are affected by changing *G*^fine^_max_. These results highlight that also the interpretation of vibrational eigenvectors, whether to establish structure–property relations when interpreting vibrational spectra,^51^ to map phonon-driven transformations,^52,53^ or otherwise, requires the careful convergence of phonon calculations.

### 3.2 Effect of *Γ*-point phonon frequency convergence on physico-chemical properties

We have so far shown that, to obtain converged phonon frequencies and eigenvectors for CHNO-containing molecular crystals in the linear response formalism, one requires considerably finer charge density representations than those dictated by the basis set kinetic energy cut-off. This, and the necessity to perform additional convergence testing, inevitably comes at the expense of increased computational cost. One cannot help but ask the question: *is it worth the effort?* The answer to this question naturally depends on the purpose of the phonon simulations and the material properties of interest. In this regard, we here explore the effects that convergence of the phonon frequencies with respect to *G*^fine^_max_ has on phonon-dependent properties. First, we probe the effect of *G*^fine^_max_ on the vibrational contributions to the crystal free energy, which depend only on the phonon density of states, and hence allows us to examine the importance of an overall converged set of frequencies. Second, we perform local mode analysis (a method to project normal modes onto internal coordinates to obtain effective local mode force constants),^54^ which depends on both the phonon frequencies and the corresponding eigenvectors.

#### 3.2.1 Phonon convergence and vibrational free energy

While density functional theory calculations are inherently performed at 0 K, we can capture some of the free energy's temperature-dependence by including the vibrational free energy using calculated phonon frequencies. Specifically, the vibrational free energy, *F*_vib_, is given by4
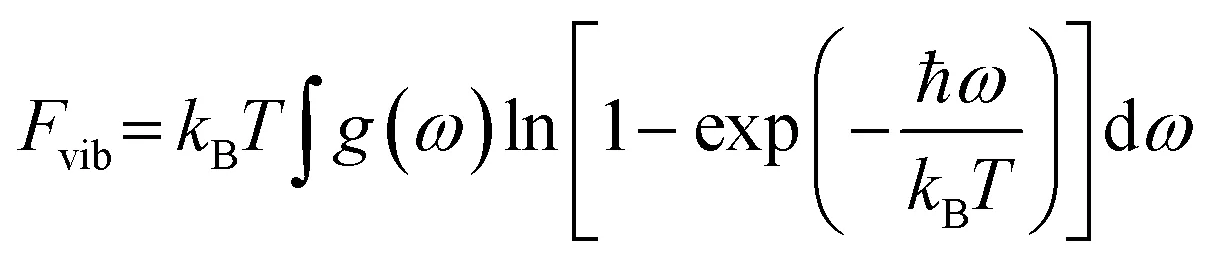
where *g*(*ω*) is the phonon density of states. The accuracy of these vibrational contributions to the crystal free energy is especially important, given that the energy differences between polymorphic forms are typically on the order of only *ca.* 2–3 kJ mol^−1^.^55–57^

**Fig. 4:**
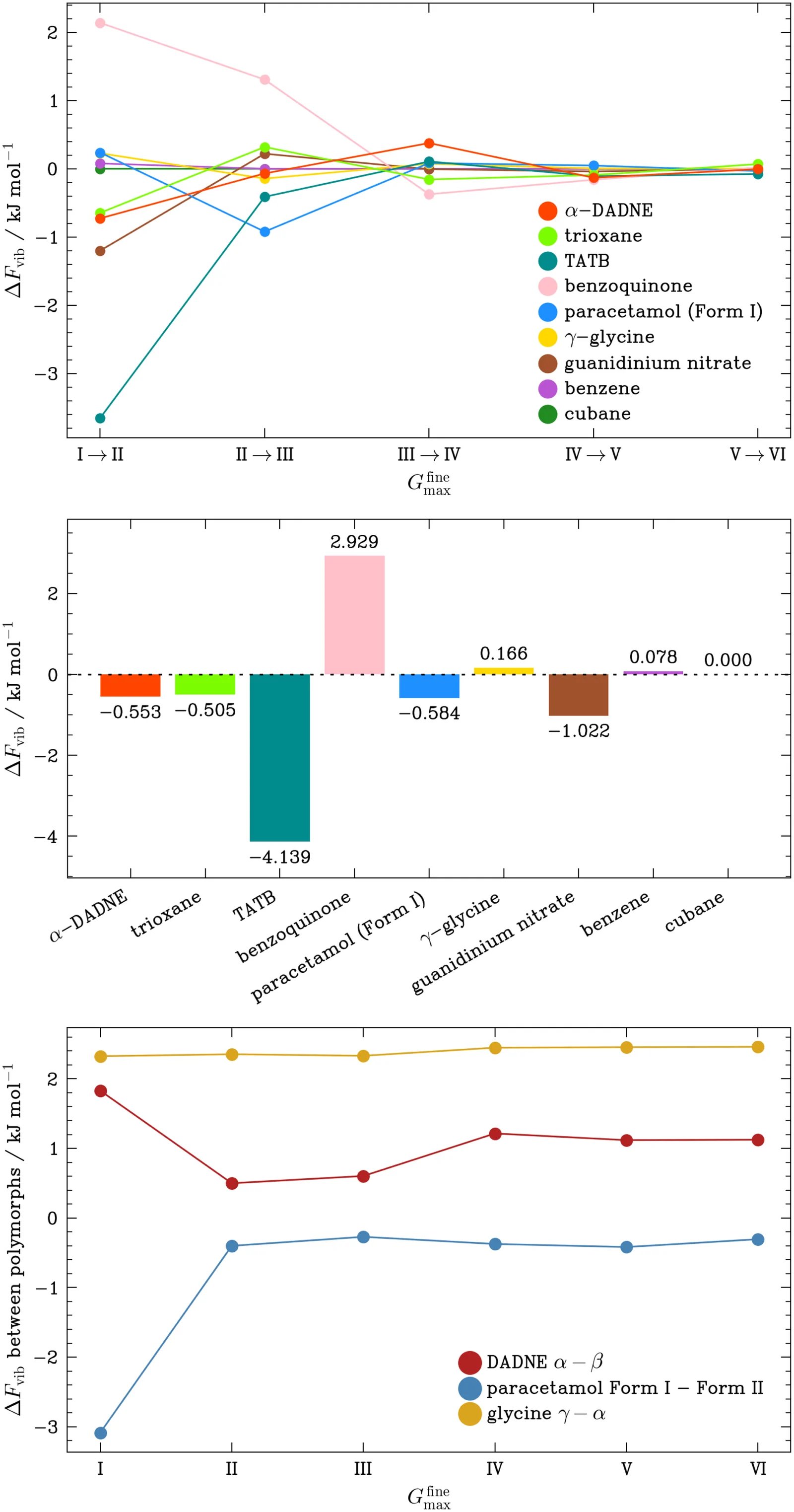
(Top) Convergence behaviour of the vibrational free energy for OMCs calculated using phonon frequencies obtained with increasing *G*^fine^_max_ (Table 1). (Middle) Change in the vibrational free energy for OMCs as *G*^fine^_max_ is increased from setting I (35.4943 Å^−1^) to setting VI (124.2302 Å^−1^). (Bottom) Difference in the vibrational free energies calculated for pairs of polymorphs of DADNE, paracetamol and glycine calculated using phonon frequencies obtained with increasing *G*^fine^_max_ (Table 1). All thermodynamic data are calculated at 300 K.

Using the phonon frequencies obtained in section 2.3, we calculated for each of our OMCs how *F*_vib_ change as *G*^fine^_max_ is increased, Fig. 4 and SI section S4 (note only small changes in zero-point energy with changes in *G*^fine^_max_). Stemming from the minimal influence that *G*^fine^_max_ has on the phonon frequencies for CH crystals, we see that *F*_vib_ for cubane and benzene is only negligibly affected by *G*^fine^_max_. For these CH systems, increasing *G*^fine^_max_ from setting I to II was accompanied by a change in *F*_vib_ of <0.1 kJ mol^−1^. In contrast, *F*_vib_ for the CHO and CHNO systems is much more sensitive to *G*^fine^_max_, again reflecting the sensitivity of their phonon frequencies to this fine grid parameter. In fact, for benzoquinone and TATB, *F*_vib_ changes by >2 kJ mol^−1^—*i.e.*, an amount that is typical of the energy difference between polymorphic forms^55^—when *G*^fine^_max_ is increased from setting I to II, and 3–4 kJ mol^−1^ when comparing setting I to VI. For all systems, increasing *G*^fine^_max_ beyond a setting of III has minimal further effect. Our results demonstrate that errors in *F*_vib_ due to insufficient convergence of phonon frequencies with respect to *G*^fine^_max_ lead to notable change in the overall predicted thermodynamic stability of OMCs, Fig. 4 (middle), and that in some cases this error may be sufficient to cause the re-ordering of predicted polymorph stabilities.

As a demonstration of this, we compute *F*_vib_ for a series of polymorphic forms at a range of *G*^fine^_max_ settings. We note that the internal energy for our OMCs is unaffected by *G*^fine^_max_, and hence the change in the predicted relative polymorph stability becomes dependent only on the relative values of *F*_vib_ as *G*^fine^_max_ is varied, which we plot in Fig. 4 (bottom) and SI section S4. For our model system (glycine) whose *F*_vib_ is only minimally affected by *G*^fine^_max_, the relative stability of the α- and γ-polymorphs is largely independent of *G*^fine^_max_. In contrast, for both DADNE and paracetamol polymorphs, a low, unconverged value of *G*^fine^_max_ estimates the difference in polymorph stability to be 1–2 kJ mol^−1^ as compared with predictions done at higher values of *G*^fine^_max_.

Using the example of the vibrational free energy, we highlight that bulk thermodynamic properties show similar convergence behaviour with respect to the quality of the charge density as the individual phonon frequencies, despite depending on the overall set of frequencies. In line with our overarching goal to highlight the sensitivity of linear-response phonon calculations to computational parameters, we emphasise the importance of rigorous convergence testing to ensure the reliability of property predictions derived from phonon calculations. It is worth noting that site symmetry splitting, prominent in low symmetry crystals, leads to the presence of a larger number of symmetry independent modes as compared with high symmetry crystals. As such, for low symmetry systems, one might expect that error in the predicted frequencies will have a larger impact on the overall thermodynamic integration.

#### 3.2.2 Local mode analysis

Having seen that *G*^fine^_max_ can significantly impact on phonon properties that depend on the overall density of states, we now consider a property based on the projection of specific frequencies and eigenvectors: bond strengths, as determined by the effective local mode force constants derived from local vibrational mode theory.^54^ Since local vibrational mode analysis is a bonding descriptor based entirely on vibrational frequencies and eigenvectors as inputs, it serves as an excellent benchmark against which to study the effect *G*^fine^_max_ has on solid state bonding analysis.

Fortunately, as we have observed in section 3.1, phonons with *ω* > 400 cm^−1^ are negligibly affected by the choice of *G*^fine^_max_. We might therefore expect that the local vibrational mode analysis of chemical bonding in OMCs will be equally insensitive to *G*^fine^_max_. Indeed, local mode analysis of our model OMCs does show only minor variation in the calculated local mode force constants (see SI section S5). For systems found in section 3.1 to require higher *G*^fine^_max_ values to achieve convergence, we see variation in local mode force constants of no more than *ca.* 0.1–0.5 mdyn Å^−1^. This variation is on the order of the differences seen for the local mode force constants associated with the aromatic C–C bonds in different polycyclic aromatic hydrocarbons,^54^ and hence can have impact on bonding studies where fine differentiation between bonding environments is required. However, the local mode force constants were unaffected by *G*^fine^_max_ for the OMCs whose phonon frequencies also exhibited minimal sensitivity to *G*^fine^_max_, such as benzene.

While we find the fortunate situation that local vibrational mode descriptors of chemical bonding are largely insensitive to *G*^fine^_max_, this insensitivity is primarily the result of current local mode analysis being restricted to intramolecular bonding analysis. We anticipate the same insensitivity will not hold if local vibrational mode analysis becomes extended to allow for the study of solid state intermolecular interactions in the future.

### 3.3 Phonon dispersion convergence with respect to Brillouin zone sampling

Having explored the convergence behaviour of Brillouin zone-centre phonon frequencies with respect to the quality of the charge density representation, we now turn our attention to the convergence of the phonon dispersion relations. Phonon dispersion relations describe the dependence of phonon frequencies, *ω*, on wavevector, **q**, obtained by diagonalising the dynamical matrix at each sampled *q*-point in the Brillouin zone. While in theory a complete representation of the phonon dispersion would necessitate an infinite number of dynamical matrices to be calculated, in practice we explicitly calculate the dynamical matrices for a small subset of *q*-points, and Fourier-interpolate between these points to generate a finer grid. While a sufficiently large *G*^fine^_max_ ensures well-converged frequencies at individual wavevectors, the phonon dispersion depends on the selection of *q*-points for which dynamical matrices are explicitly calculated. A sufficiently dense *q*-point mesh is necessary to accurately interpolate phonon branches across the Brillouin zone. Insufficient sampling can lead to artificial distortions in the dispersion curves, affecting the accuracy of derived properties—but the extent of this effect is not well explored. Here, we examine how the choice of *q*-point sampling for the explicit computation of the dynamical matrices influences the construction of the phonon dispersion, using a subset of our model OMCs: benzoquinone, γ-glycine, benzene, and cubane. The explicit computation of dynamical matrices was done using *G*^fine^_max_ values for which *Γ*-point frequency convergence within Δ*ω ca.* <1 cm^−1^ was achieved (benzoquinone and γ-glycine: V, benzene: II, cubane: I).

Dynamical matrices were explicitly calculated on increasingly finer *Γ*-centred Monkhorst–Pack (MP) *q*-point grids, generated based on a gradually decreasing *q*-point spacing, SI section S6.1, and the force constants were interpolated onto high-symmetry Brillouin zone paths with 0.05 Å^−1^ step size to produce the phonon dispersion plots, SI section S6.2. For all of our selected OMCs (except cubane), imaginary frequencies were obtained when the phonon dispersion was interpolated from *Γ*-point phonons only. We found that all phonon branches in our OMCs had positive frequencies when at least two *q*-points were explicitly sampled along the largest reciprocal space lattice vectors (achieved with MP grid spacing of 0.14 Å^−1^). This *q*-point grid spacing (0.14 Å^−1^) was therefore used as the starting point for our further comparisons.

**Fig. 5:**
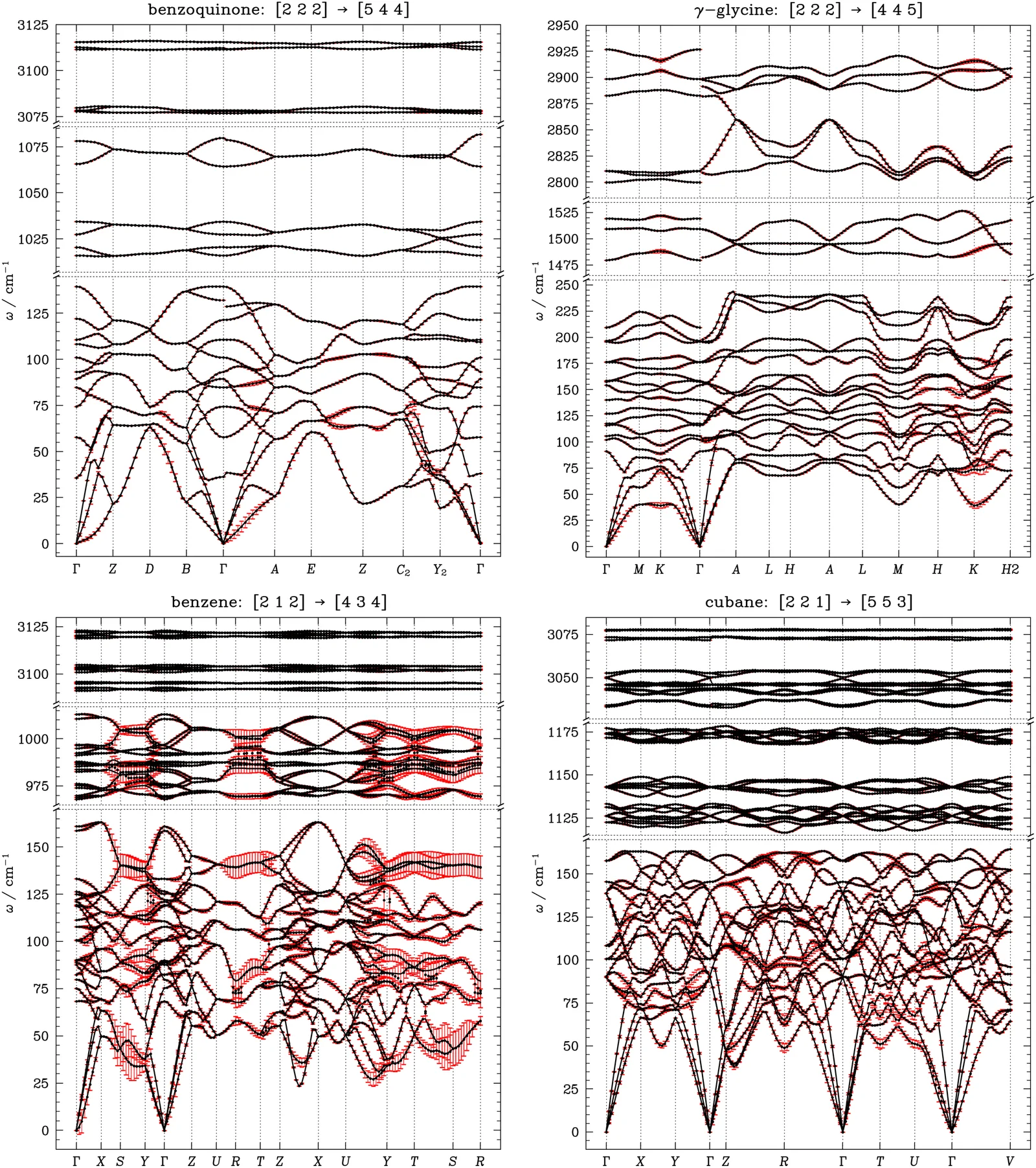
Change in phonon dispersion as *q*-point sampling in the Brillouin zone is increased. Phonon frequencies averaged between the coarser and finer *q*-point spacing are shown as black dots, with the red ‘error bars’ representing the maximum and minimum value of a given frequency at the corresponding *q*-point, with frequencies matched based eigenvector. Black lines are drawn as a guide for the eye and do not strictly conserve band connectivity. Only selected portions of the phonon dispersion are displayed to represent the convergence behaviour in the low, intermediate, and high frequency range. Further detail is given in SI section S6.2.

As we increase the density of *q*-point sampling from a spacing of 0.14 Å^−1^ to 0.04 Å^−1^ (the finest MP grid we sampled), we see substantial variation in the interpolated phonon branch frequencies in the weakly, dispersion-bonded OMCs, cubane and benzene, Fig. 5. This is most prominent in benzene, where variations of Δ*ω* > 20 cm^−1^ are observed across a significant portion of the Brillouin zone, and for branches with average frequencies up to *ca.* 1100 cm^−1^. The impact of increasing the *q*-point sampling density in cubane is somewhat less significant, with Δ*ω* on the order of *ca.* 5–10 cm^−1^ for branches with average frequency *ca.* <200 cm^−1^, but seen across the entire Brillouin zone. In stark contrast, we find that changing the *q*-point spacing for explicitly calculated dynamical matrices has much less impact on the interpolated phonon branches for benzoquinone and γ-glycine, which contain hydrogen bonds (albeit weak CH⋯O bonds in benzoquinone). In fact, while we do see Δ*ω* on the order of 5–10 cm^−1^ as we decrease the *q*-point sampling spacing, this remains localised to very small segments of the phonon dispersion curve; across the majority of the dispersion curves for benzoquinone and γ-glycine we only observe Δ*ω* on the order of 1–2 cm^−1^, SI section S6.2. In all the OMCs, it is important to note that the response of the phonon branch frequencies is not linear with increasing *q*-point grid, with branch frequencies showing much more significant responses to the change in *q*-point sampling densities for coarser grids. We tend to find that using *q*-point grids with spacings ≈<0.06 Å^−1^ yield overall little additional effect on the branch frequencies, albeit with the more weakly bound systems (benzene and cubane) still remaining less well-converged compared to benzoquinone and γ-glycine, SI section S6.

The difference in convergence behaviour of the phonon dispersion relation seen across the OMCs can be attributed to the fact that in strongly bonded crystals, the dynamical matrix is well-defined by the presence of several strongly interacting moieties, such as those involved in hydrogen bonding. These strong interactions influence molecular motion over long ranges, leading to phonon dispersions that tend to be less sensitive to changes in the *q*-point sampling. In contrast, for OMCs where weak interactions play a dominant role, phonon spectra rely on accurately resolving all relevant interatomic force constants, and as a result, greater frequency variations are observed when the Brillouin zone grid spacing is reduced. Whilst we have only a limited dataset, we do not see any obvious correlation between the ease of phonon dispersion convergence and the crystal symmetry. This is reflected in the fact that our low symmetry (monoclinic) system, benzoquinone, converges similarly to our higher symmetry systems, benzene (orthorhombic) and cubane (rhombohedral).

**Fig. 6:**
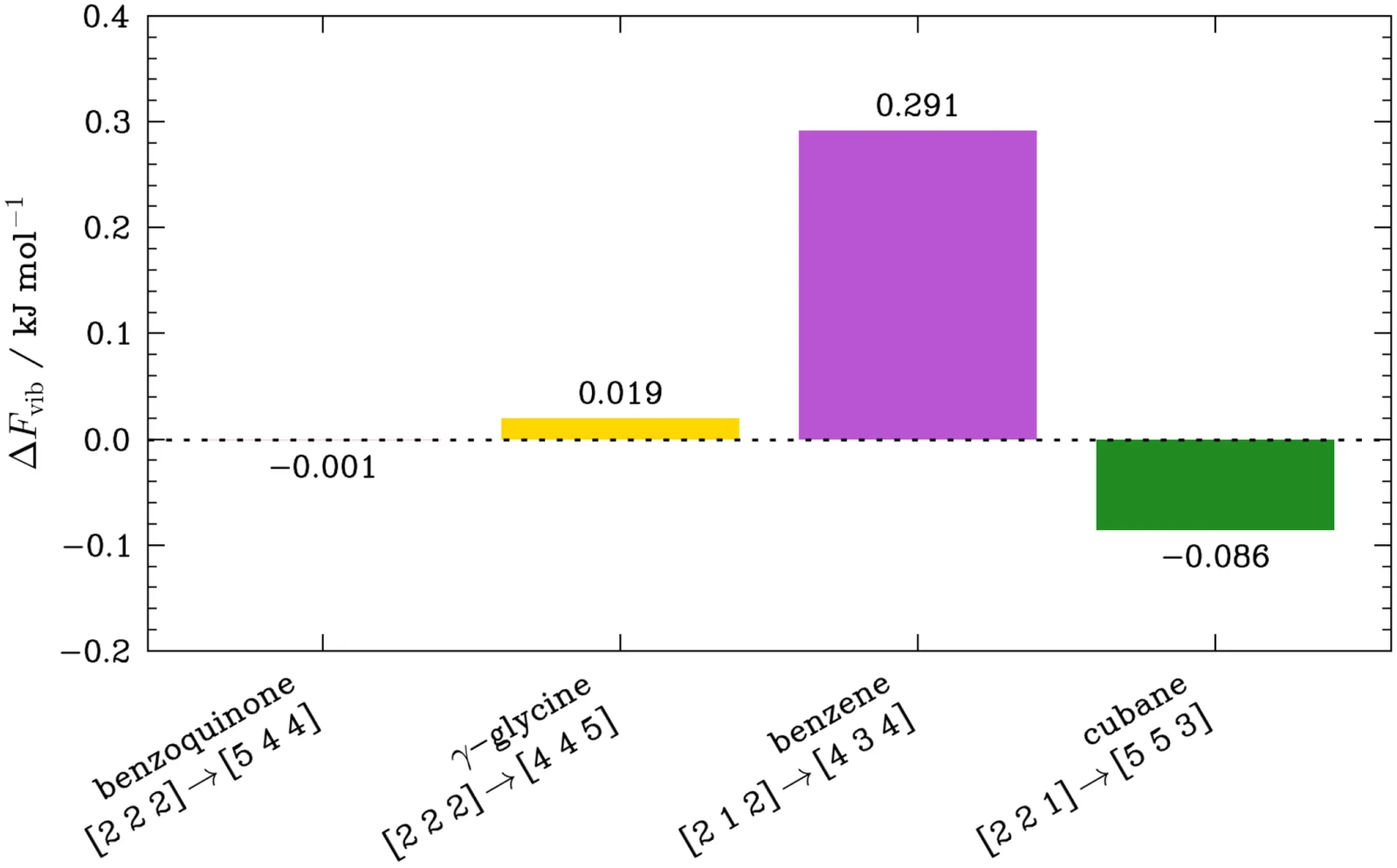
Change in the vibrational free energy for OMCs as *q*-point sampling in the Brillouin zone is increased from the coarsest MP grid yielding a stable dispersion (*ca.* 0.14 Å^−1^*q*-point spacing) and the finest MP grid used in this study (*ca.* 0.04 Å^−1^*q*-point spacing), limited by computational cost.

Finally, we turn our attention once again to the vibrational contributions to the free energy of the OMCs, and investigate the variation in *F*_vib_ calculated from the stable dispersion relations interpolated from the *q*-point grids discussed above, Fig. 6. In contrast to the large Δ*F*_vib_ observed when *Γ*-point phonon frequencies calculated with different *G*^fine^_max_ were used, the largest Δ*F*_vib_ in the subset, seen for benzene, is only 0.291 kJ mol^−1^ despite the notable Δ*ω* variations in the dispersion plots. This can be attributed to the phonon frequencies being well-defined at the MP grid points due to a sufficiently fine charge density representation, ensuring that any differences in the interpolation between the explicitly sampled *q*-points approximately average out. While this is expected for bulk properties like *F*_vib_, more significant errors can be expected in the prediction of anisotropic properties that depend intimately on the degree of dispersion in the phonon branches, like thermal transport,^58^ though this is beyond the scope of the present work.

## 4 Conclusions

We have here explored the convergence behaviour of simulated phonon frequencies and eigenvectors, as obtained through linear response theory in the plane wave density functional theory formalism. In particular, we have probed the sensitivity of both frequencies and eigenvectors to the construction of the charge density grid, a parameter that is often overlooked in literature simulation of phonon properties for organic molecular crystals. We demonstrate that errors on the order of tens of wavenumbers can arise for low-frequency phonons when coarse charge-density grids are used. These errors have significant repercussions for the prediction of thermodynamic quantities. For some of our modelled systems, the errors in thermodynamic quantities that arise from the insufficient convergence of calculated phonon frequencies are on the order of 3–4 kJ mol^−1^, which is comparable to differences in polymorph stabilities in organic molecular crystals. We demonstrate how the predicted relative stability between polymorphic forms can differ significantly, depending on the degree of convergence in the charge density grid. This finding exemplifies the importance of ensuring sufficient convergence of phonon frequency calculations for studying even ‘simple’ properties of organic molecular crystals. It is worth stressing that to compare polymorphic stabilities one must also take care that the electronic structure descriptors (*e.g. k*-point sampling and plane wave constructions) are chosen to ensure that the quality of the representation is equivalent for differently sized unit cells. We finally highlight the impact of *q*-point sampling density on the calculation of phonon dispersion relations for organic molecular crystals, especially in systems with weak, anisotropic intermolecular interactions.

Our studies have been restricted to a single density functional (the generalised gradient approximation functional, PBE) with Grimme's D2 dispersion correction. As the selection of functional and dispersion correction has usually only a small influence on the charge density distribution, and often limited to the low oscillatory components of the charge density structure, we do not anticipate that the convergence behaviour observed in this work will be significantly different for different levels of theory. However, we do stress that the convergence behaviour is intimately linked to the choice of pseudopotential, and hence our work shows only the importance of converging this parameter carefully for a given simulation set-up. Moreover, our work has only considered the impact of this convergence behaviour on the harmonic force constants, but will undoubtedly have an equally important impact on anharmonic force constants. Not only will the inclusion of anharmonic effects influence the accuracy of computed frequencies, but the accurate convergence of anharmonic force constants will be essential should more complex behaviour be sought, including accurate simulations of thermal conductivity and spectral line widths.

Our investigation does not hone in on clear rules for achieving convergence with respect to the charge density representation or *q*-point sampling. However, we hope that our study will serve as an important demonstration of the significance that these parameters have in the simulation of phonon frequencies within the linear response formalism when using a plane wave basis set. Moreover, we hope that our results contribute to a clearer understanding of the potential magnitude of errors that may be inherent to simulated phonon frequencies where full convergence testing of the demonstrated parameters may be prohibitively expensive.

## Conflicts of interest

There are no conflicts to declare.

## Supplementary Material

CE-028-D5CE01090J-s001

CE-028-D5CE01090J-s002

## Data Availability

All data associated with this study are available in the supplementary information (SI), or can be obtained at the Zenodo repository, at DOI: https://doi.org/10.5281/zenodo.16631359. Supplementary information is available. See DOI: https://doi.org/10.1039/d5ce01090j.
