# Supplementary material for: Converging ab initio phonon simulations for organic molecular crystals: the effect of charge density grids and phonon dispersion sampling
Source: CrystEngComm. 2025 Dec 3;28(2):547–58. doi: 10.1039/d5ce01090j (PMC13580395; doi:10.1039/d5ce01090j)
Supplement: Supplementary file 1 [file CE-028-D5CE01090J-s001.pdf]

Supplementary Information (SI) for CrystEngComm.
This journal is © The Royal Society of Chemistry 2025

Converging ab initio phonon simulations for organic molecular crystals: the effect of charge density grids and phonon dispersion sampling

Mateusz Mojsak,[a] Tahlia M. Palmer,[a] and Adam A. L. Michalchuk*[a,b]
[a] School of Chemistry, University of Birmingham, Birmingham, UK, B15 2TT
[b] Federal Institute for Materials Research and Testing (BAM), Berlin, Germany

Contents

S1 Structure Optimisation 2

S2 Comparison of Simulated and Experimental Inelastic Neutron Scattering Spectroscopy Data 7

S3 Effect of G_{max}^{fine} on the Γ-point phonon frequencies 10
S3.1 Comparison of phonon frequencies calculated with increasing G_{max}^{fine} 10
S3.2 Summary of frequency convergence to < 0.5 cm^{-1}, < 2 cm^{-1} and 5 cm^{-1} with respect to increasing G_{max}^{fine} 14
S3.3 Comparison of phonon density of states calculated with increasing G_{max}^{fine} . 15

S4 Thermodynamic data 20

S5 Local Vibrational Mode Analysis 23

S6 Converging Phonon Dispersion Behaviour 26
S6.1 Phonon dispersion parameters and thermodynamic behaviour 26
S6.2 Phonon dispersion calculated with different q-point grids. 28
S6.2.1 Phonon dispersion for γ-glycine 28
S6.2.2 Phonon dispersion for benzoquinone 30
S6.2.3 Phonon dispersion for benzene 32
S6.2.4 Phonon dispersion for cubane 34

S1 Structure Optimisation

For each of our simulations, we used a wave function generated from a plane wave expansion to a maximum kinetic energy cut-off of 1200 eV, with associated $G_{\text{max}} = 17.7472$ Å^{-1}, as defined by

$$G_{\text{max}} = S \times \sqrt{\frac{E_{\text{cut}} \times 2m_e}{\hbar^2}} \tag{S1.1}$$

with scaling coefficient $S = 1$. Using this wavefunction, we maintained a standard Fourier grid $G^{\text{std}}_{\text{max}}$ for total energy analysis by setting $S = 2$ in Equation S1.1, whilst varying the value of S to generate a series of fine Fourier grids $G^{\text{fine}}_{\text{max}}$ for the generation of the real-space charge density, Table S1.1.

Table S1.1. Parameters for $G^{\text{fine}}_{\text{max}}$ use to calculate phonon frequencies. The size of $G^{\text{fine}}_{\text{max}}$ is obtained from Equation S1.1 by substituting the grid scale parameter for S. In all cases, a value of $E_{\text{cut}} = 1200$eV was used for plane wave expansion, corresponding to a value of $G_{\text{max}} = 17.7472$ Å^{-1}. For all calculations $G^{\text{std}}_{\text{max}}$ was set using $S = 2$.

Grid Label	Fine grid scale	Size of fine G_{max} / Å^{-1}
I	2.0	35.4943
II	3.0	53.2415
III	4.0	70.9887
IV	5.0	88.7359
V	6.0	106.483
VI	7.0	124.2302

Using the charge density grids in Table S1.1, we optimised each of our crystal structures at PBE-D2 level of theory (see main text Methods for details), and compare these against experimental geometries in Tables S1.2 (α-DADNE), S1.3 (β-DADNE), S1.4 (trioxane), S1.5 (TATB), S1.6 (benzoquinone), S1.7 (paracetamol Form I), S1.8 (paracetamol Form II), S1.9 (γ-glycine), S1.10 (α-glycine), S1.11 (guanidinium nitrate), S1.12 (benzene), S1.13 (cubane). In all cases the volume of our simulated crystal structures was within *ca* 5 % of the low temperature experimental structures, except for the dispersion-bound OMCs (benzene and cubane), for which simulated geometries differ by *ca* 10 %. Moreover we note that for all of our systems, an increase in resulted in no meaningful change in crystal structure geometry, thereby ensuring any changes in phonon frequencies are not related to variation in crystal structure geometries.

Table S1.2. Comparison of experimental (Exp) and optimised (Calc) unit cell geometries for α-**DADNE** at different fine charge density grids. Note the experimental geometry was measured at 200 K (ICSD collection code 172530).[1]

	Fine grid scale	a / Å	b / Å	c / Å	α /deg.	β /deg.	γ /deg.	Vol / Å^3
Exp		6.9209	6.5515	11.2741	90	90.0600	90	511.193
Calc	2.0	7.0115	6.4864	11.3095	90	90.9357	90	514.2817
Δ%	2.0	+1.309	−0.994	+0.314	–	+0.972	–	+0.604
Calc	3.0	7.0077	6.4853	11.3060	90	90.9521	90	513.7468
Δ%	3.0	+1.254	−1.010	+0.283	–	+0.991	–	+0.500
Calc	4.0	7.0092	6.4861	11.3085	90	90.9292	90	513.0489
Δ%	4.0	+1.276	−0.998	+0.305	–	+0.965	–	+0.363
Calc	5.0	7.0090	6.4856	11.3104	90	90.9405	90	514.0785
Δ%	5.0	+1.273	−1.006	+0.322	–	+0.978	–	+0.564
Calc	6.0	7.0089	6.4854	11.3109	90	90.9423	90	514.0736
Δ%	6.0	+1.272	−1.009	+0.326	–	+0.980	–	+0.564
Calc	7.0	7.0089	6.4854	11.3109	90	90.9421	90	514.0748
Δ%	7.0	+1.272	−1.009	+0.326	–	+0.979	–	+0.564

Table S1.3. Comparison of experimental (Exp) and optimised (Calc) unit cell geometries for *β*-**DADNE** at different fine charge density grids. Note the experimental geometry was measured at 393 K (ICSD collection code 172534).[1]

	Fine grid scale	a /Å	b /Å	c /Å	α /deg.	β /deg.	γ /deg.	Vol / Å^3
Exp		6.9738	6.6350	11.6475	90	90	90	538.94
Calc	2.0	7.0275	6.3563	11.6902	90	90	90	522.1825
Δ%	2.0	+0.770	−4.200	+0.367	–	–	–	−3.109
Calc	3.0	7.0237	6.3589	11.6764	90	90	90	521.4994
Δ%	3.0	+0.715	−4.161	+0.248	–	–	–	−3.236
Calc	4.0	7.0238	6.3600	11.6742	90	90	90	521.5030
Δ%	4.0	+0.717	−4.145	+0.229	–	–	–	−3.236
Calc	5.0	7.0246	6.3592	11.6741	90	90	90	521.4919
Δ%	5.0	+0.728	−4.157	+0.228	–	–	–	−3.238
Calc	6.0	7.0247	6.3591	11.6751	90	90	90	521.5303
Δ%	6.0	+0.730	−4.158	+0.237	–	–	–	−3.230
Calc	7.0	7.0246	6.3591	11.6748	90	90	90	521.5183
Δ%	7.0	+0.728	−4.158	+0.234	–	–	–	−3.232

Table S1.4. Comparison of experimental (Exp) and optimised (Calc) unit cell geometries for **trioxane** at different fine charge density grids. Note the experimental geometry was measured at 103 K (CCDC REF: TROXAN11).[2]

	Fine Grid Scale	a /Å	b /Å	c /Å	α /deg.	β /deg.	γ /deg.	Vol / Å^3
Exp		6.0347	6.0347	6.0347	101.1032	101.1032	101.1032	205.5149
Calc	2.0	5.9303	5.9303	5.9303	101.2369	101.2369	101.2369	194.6780
Δ%	2.0	−1.730	−1.730	−1.730	+0.132	+0.132	+0.132	-5.273
Calc	3.0	5.9307	5.9307	5.9307	101.2418	101.2418	101.2418	194.7003
Δ%	3.0	−1.723	−1.723	−1.723	+0.137	+0.137	+0.137	-5.262
Calc	4.0	5.9302	5.9302	5.9302	101.2342	101.2342	101.2342	194.6705
Δ%	4.0	−1.732	−1.732	−1.732	+0.130	+0.130	+0.130	-5.277
Calc	5.0	5.9301	5.9301	5.9301	101.2330	101.2330	101.2330	194.6627
Δ%	5.0	−1.733	−1.733	−1.733	+0.128	+0.128	+0.128	-5.280
Calc	6.0	5.9301	5.9301	5.9301	101.2340	101.2340	101.2340	194.6654
Δ%	6.0	−1.733	−1.733	−1.733	+0.129	+0.129	+0.129	-5.279
Calc	7.0	5.9301	5.9301	5.9301	101.2340	101.2340	101.2340	194.6654
Δ%	7.0	−1.733	−1.733	−1.733	+0.129	+0.129	+0.129	-5.279

Table S1.5. Comparison of experimental (Exp) and optimised (Calc) unit cell geometries for **TATB** at different fine charge density grids. Note the experimental geometry was measured at 20 K (CCDC REF: TATNBZ03).[3]

	Fine Grid Scale	a /Å	b /Å	c /Å	α /deg.	β /deg.	γ /deg.	Vol / Å^3
Exp		8.9970	9.0201	6.5975	108.870	92.384	119.960	425.252
Calc	2.0	9.1006	9.1140	6.6096	109.3862	91.9736	119.9135	434.6281
Δ%	2.0	+1.151	+1.041	+0.183	+0.474	−0.444	−0.039	+2.205
Calc	3.0	9.1012	9.1143	6.6104	109.4748	91.7325	119.9425	434.7967
Δ%	3.0	+1.158	+1.044	+0.196	+0.556	−0.705	−0.015	+2.244
Calc	4.0	9.1009	9.1149	6.6090	109.4486	91.7494	119.9409	434.7942
Δ%	4.0	+1.155	+1.051	+0.174	+0.531	−0.687	−0.016	+2.244
Calc	5.0	9.1013	9.1149	6.6093	109.4674	91.7244	119.9419	434.8083
Δ%	5.0	+1.159	+1.051	+0.179	+0.549	−0.714	−0.015	+2.247
Calc	6.0	9.1013	9.1149	6.6092	109.4647	91.7284	119.9420	434.8001
Δ%	6.0	+1.159	+1.051	+0.177	+0.546	−0.710	−0.015	+2.245
Calc	7.0	9.1012	9.1148	6.6091	109.4628	91.7312	119.9415	434.7940
Δ%	7.0	+1.158	+1.050	+0.176	+0.545	−0.707	−0.015	+2.244

Table S1.6. Comparison of experimental (Exp) and optimised (Calc) unit cell geometries for **benzoquinone** at different fine charge density grids. Note the experimental geometry was measured at 173 K (CCDC REF: BNZQUI03).[4]

	Fine Grid Scale	a / Å	b / Å	c / Å	α /deg.	β /deg.	γ /deg.	Vol / Å^3
Exp		5.7430	6.7710	6.8670	90	100.1400	90	262.8583
Calc	2.0	5.7666	6.6393	6.5756	90	97.3097	90	249.7129
Δ%	2.0	+0.411	−1.945	−4.243	–	−2.826	–	−5.001
Calc	3.0	5.7628	6.6389	6.5708	90	97.2432	90	249.3831
Δ%	3.0	+0.345	−1.951	−4.313	–	−2.893	–	−5.126
Calc	4.0	5.7671	6.6403	6.5741	90	97.3241	90	249.7016
Δ%	4.0	+0.420	−1.930	−4.265	–	−2.812	–	−5.005
Calc	5.0	5.7661	6.6399	6.5744	90	97.3000	90	249.6675
Δ%	5.0	+0.402	−1.936	−4.261	–	−2.836	–	−5.018
Calc	6.0	5.7660	6.6397	6.5739	90	97.2941	90	249.6426
Δ%	6.0	+0.400	−1.939	−4.268	–	−2.842	–	−5.028
Calc	7.0	5.7660	6.6398	6.5739	90	97.2941	90	249.6426
Δ%	7.0	+0.400	−1.938	−4.268	–	−2.842	–	−5.028

Table S1.7. Comparison of experimental (Exp) and optimised (Calc) unit cell geometries for **paracetamol (Form I)** at different fine charge density grids. Note the experimental geometry was measured at 173 K (CCDC REF: HXACAN36).[5]

	Fine grid scale	a / Å	b / Å	c / Å	α /deg.	β /deg.	γ /deg.	Vol / Å^3
Exp		7.0842	9.1734	11.5470	90	97.99	90	743.1147
Calc	2.0	6.9958	8.7889	11.6448	90	98.96	90	707.2571
Δ %	2.0	−1.248	−4.191	+0.847	–	+0.992	–	−4.825
Calc	3.0	6.9945	8.7884	11.6414	90	98.94	90	706.9141
Δ %	3.0	−1.266	−4.197	+0.818	–	+0.972	–	−4.871
Calc	4.0	6.9946	8.7879	11.6416	90	98.93	90	706.9002
Δ %	4.0	−1.265	−4.202	+0.819	–	+0.961	–	−4.873
Calc	5.0	6.9946	8.7881	11.6403	90	98.91	90	706.8923
Δ %	5.0	−1.265	−4.200	+0.808	–	+0.941	–	−4.874
Calc	6.0	6.9948	8.7880	11.6401	90	98.91	90	706.8925
Δ %	6.0	−1.262	−4.201	+0.806	–	+0.941	–	−4.874
Calc	7.0	6.9948	8.7880	11.6401	90	98.91	90	706.8923
Δ %	7.0	−1.262	−4.201	+0.806	–	+0.941	–	−4.874

Table S1.8. Comparison of experimental (Exp) and optimised (Calc) unit cell geometries for **paracetamol (Form II)** at different fine charge density grids. Note the experimental geometry was measured at 123 K (CCDC REF: HXACAN08).[6]

	Fine grid scale	a / Å	b / Å	c / Å	α /deg.	β /deg.	γ /deg.	Vol / Å^3
Exp		17.1657	11.7773	7.2120	90	90	90	1458.0183
Calc	2.0	17.2188	11.5234	6.8954	90	90	90	1368.1887
Δ %	2.0	+0.309	−2.156	−4.389	–	–	–	−6.161
Calc	3.0	17.2236	11.5203	6.8919	90	98.94	90	1367.4902
Δ %	3.0	+0.338	−2.183	−4.439	–	–	–	−6.209
Calc	4.0	17.2261	11.5198	6.8894	90	90	90	1367.131
Δ %	4.0	+0.352	−2.187	−4.474	–	–	–	−6.234
Calc	5.0	17.2252	11.5198	6.89062	90	90	90	1367.3092
Δ %	5.0	+0.347	−2.187	−4.474	–	–	–	−6.221
Calc	6.0	17.2259	11.5197	6.8900	90	90	90	706.8925
Δ %	6.0	+0.350	−2.188	−4.465	–	–	–	−6.227
Calc	7.0	17.2258	11.5196	6.8902	90	90	90	1367.2576
Δ %	7.0	+0.350	−2.188	−4.462	–	–	–	−6.225

Table S1.9. Comparison of experimental (Exp) and optimised (Calc) unit cell geometries for γ-**glycine** at different fine charge density grids. Note the experimental geometry was measured at 20 K (CCDC REF: GLYCIN15).[7]

	Fine grid scale	a /Å	b /Å	c /Å	α /deg.	β /deg.	γ /deg.	Vol / Å^3
Exp		7.046	7.046	5.491	90	90	120	236.084
Calc	2.0	6.9156	6.9156	5.4968	90	90	120	227.6707
Δ%	2.0	−1.851	−1.851	+0.106	–	–	–	−3.564
Calc	3.0	6.9163	6.9163	5.4968	90	90	120	227.7135
Δ%	3.0	−1.841	−1.841	+0.106	–	–	–	−3.546
Calc	4.0	6.9159	6.9159	5.4964	90	90	120	227.6729
Δ%	4.0	−1.846	−1.846	+0.098	–	–	–	−3.563
Calc	5.0	6.9160	6.9160	5.4964	90	90	120	227.6736
Δ%	5.0	−1.845	−1.845	+0.098	–	–	–	−3.562
Calc	6.0	6.9160	6.9160	5.4964	90	90	120	227.6718
Δ%	6.0	−1.845	−1.845	+0.098	–	–	–	−3.563
Calc	7.0	6.9160	6.9160	5.4964	90	90	120	227.6718
Δ%	7.0	−1.845	−1.845	+0.098	–	–	–	−3.563

Table S1.10. Comparison of experimental (Exp) and optimised (Calc) unit cell geometries for α-**glycine** at different fine charge density grids. Note the experimental geometry was measured at room temperature (CCDC REF: GLYCIN04).[8]

	Fine grid scale	a /Å	b /Å	c /Å	α /deg.	β /deg.	γ /deg.	Vol / Å^3
Exp		5.1054	11.9688	5.4645	90	111.6970	90	310.2540
Calc	2.0	5.0545	11.7555	5.4640	90	112.8628	90	299.1527
Δ%	2.0	−0.997	−1.782	−0.010	–	+0.376	–	−3.578
Calc	3.0	5.0549	11.7553	5.4642	90	112.8721	90	299.1607
Δ%	3.0	−0.989	−1.784	−0.006	–	+0.379	–	−3.576
Calc	4.0	5.0545	11.7560	5.4640	90	112.8732	90	299.1436
Δ%	4.0	−0.996	−1.778	−0.010	–	+0.379	–	−3.581
Calc	5.0	5.0543	11.7556	5.4640	90	112.8789	90	299.1086
Δ%	5.0	−1.002	−1.781	−0.009	–	+0.381	–	−3.592
Calc	6.0	5.0543	11.7555	5.4640	90	112.8788	90	299.1121
Δ%	6.0	−1.000	−1.782	−0.009	–	+0.381	–	−3.591
Calc	7.0	5.0544	11.7555	5.4640	90	112.8789	90	299.1135
Δ%	7.0	−1.000	−1.782	−0.008	–	+0.381	–	−3.591

Table S1.11. Comparison of experimental (Exp) and optimised (Calc) unit cell geometries for **guanidinium nitrate** at different fine charge density grids. Note the experimental geometry was measured at room temperature (CCDC REF: LETGIA).[9]

	Fine grid scale	a /Å	b /Å	c /Å	α /deg.	β /deg.	γ /deg.	Vol / Å^3
Exp		7.3117	7.3117	3.6290	116.4139	116.4139	59.6589	143.7480
Calc	2.0	7.3171	7.3171	5.4640	134.9972	134.9972	59.9901	138.5818
Δ%	2.0	+0.073	+0.073	+42.636	+15.963	+15.963	+0.555	−3.594
Calc	3.0	7.3159	7.3159	5.1727	135.0007	135.0007	60.0100	138.4243
Δ%	3.0	+0.057	+0.057	+42.538	+15.966	+15.966	+0.589	−3.703
Calc	4.0	7.3160	7.3160	5.1724	135.0023	135.0023	60.0035	138.4138
Δ%	4.0	+0.059	+0.059	+42.530	+15.968	+15.968	+0.578	−3.711
Calc	5.0	7.3162	7.3162	5.1727	135.0056	135.0056	59.9980	138.4119
Δ%	5.0	+0.062	+0.062	+42.537	+15.970	+15.970	+0.568	−3.712
Calc	6.0	7.3162	7.3162	5.1727	135.0058	135.0058	59.9979	138.4119
Δ%	6.0	+0.062	+0.062	+42.537	+15.971	+15.971	+0.568	−3.712
Calc	7.0	7.3162	7.3162	5.4640	135.0054	135.0054	59.9982	138.4143
Δ%	7.0	+0.062	+0.062	+42.538	+15.970	+15.970	+0.569	−3.710

Table S1.12. Comparison of experimental (Exp) and optimised (Calc) unit cell geometries for **benzene** at different fine charge density grids. Note the experimental geometry was measured at 100 K (CCDC REF: BENZEN20).[10]

	Fine grid scale	a /Å	b /Å	c /Å	α /deg.	β /deg.	γ /deg.	Vol / Å^3
Exp		7.4093	9.4541	6.7809	90	90	90	474.99
Calc	2.0	7.1404	9.0932	6.4559	90	90	90	419.1774
Δ%	2.0	−3.629	−3.817	−4.793	–	–	–	−11.75
Calc	3.0	7.1389	9.0901	6.4526	90	90	90	418.7305
Δ%	3.0	−3.649	−3.850	−4.842	–	–	–	−11.844
Calc	4.0	7.1890	9.0900	6.4526	90	90	90	418.7297
Δ%	4.0	−3.649	−3.851	−4.842	–	–	–	−11.845
Calc	5.0	7.1387	9.0899	6.4529	90	90	90	418.7299
Δ%	5.0	−3.652	−3.852	−4.8370	–	–	–	−11.845
Calc	6.0	7.1390	9.0901	6.4526	90	90	90	418.7323
Δ%	6.0	−3.648	−3.850	−4.8420	–	–	–	−11.8440

Table S1.13. Comparison of experimental (Exp) and optimised (Calc) unit cell geometries for **cubane** at different fine charge density grids. Note the experimental geometry was measured at 93 K (CCDC REF: CUBANE01).[11]

	Fine grid scale	a /Å	b /Å	c /Å	α /deg.	β /deg.	γ /deg.	Vol / Å^3
Exp		6.199	6.199	11.440	90	90	120	380.727
Calc	2.0	5.933	5.933	10.986	90	90	120	334.871
Δ%	2.0	−4.296	−4.296	−3.968	–	–	–	−12.044
Calc	3.0	5.933	5.933	10.986	90	90	120	334.871
Δ%	3.0	−4.296	−4.296	−3.968	–	–	–	−12.044
Calc	4.0	5.933	5.933	10.986	90	90	120	334.871
Δ%	4.0	−4.296	−4.296	−3.968	–	–	–	−12.044
Calc	5.0	5.933	5.933	10.986	90	90	120	334.871
Δ%	5.0	−4.296	−4.296	−3.968	–	–	–	−12.044
Calc	6.0	5.933	5.933	10.986	90	90	120	334.871
Δ%	6.0	−4.296	−4.296	−3.968	–	–	–	−12.044

S2 Comparison of Simulated and Experimental Inelastic Neutron Scattering Spectroscopy Data

Where possible, our simulated Γ-point phonon frequencies were benchmarked against inelastic neutron scattering (INS) spectra (G_{max}^{fine} = 124.2302 Å^{-1}; for full details, see the Methods section in the main text). This was done to ensure that our simulated phonon frequencies were representative of experimental vibrational spectra for this class of OMC. In addition, we compared the low-frequency region (up to *ca* 200 cm^{-1}) of the experimental vibrational spectra with the simulated frequencies obtained using the lowest (G_{max}^{fine} = 35.4943 Å^{-1}) and highest (G_{max}^{fine} = 124.2302 Å^{-1}) fine grid cut-offs tested. These low-frequency phonons provide the most significant contributions to the vibrational free energies of the OMCs. Based on data available in the literature, this benchmarking was possible for α-DADNE (Figure S2.1), TATB (Figure S2.2), trixoane (Figure S2.3), γ-glycine (Figure S2.4), α-glycine (Figure S2.5) and benzene (Figure S2.6).

As the simulations used for this comparison are based only on Γ-point frequencies, there is an expected discrepancy in the intensities of our simulated and experimental spectra. However, there is overall good agreement between the frequencies in all cases, indicating that PBE-D2 provides a good representation for modelling OMC phonon spectra. We do note a slight discrepancy in the predicted and experimental vibrational spectra of γ-glycine (*i.e.*, the absence of a simulated frequency at *ca* 600 cm^{-1}). We suspect this results from the hardening of the simulated vibrational mode, which amalgamates with the *ca* 700 cm^{-1} band.

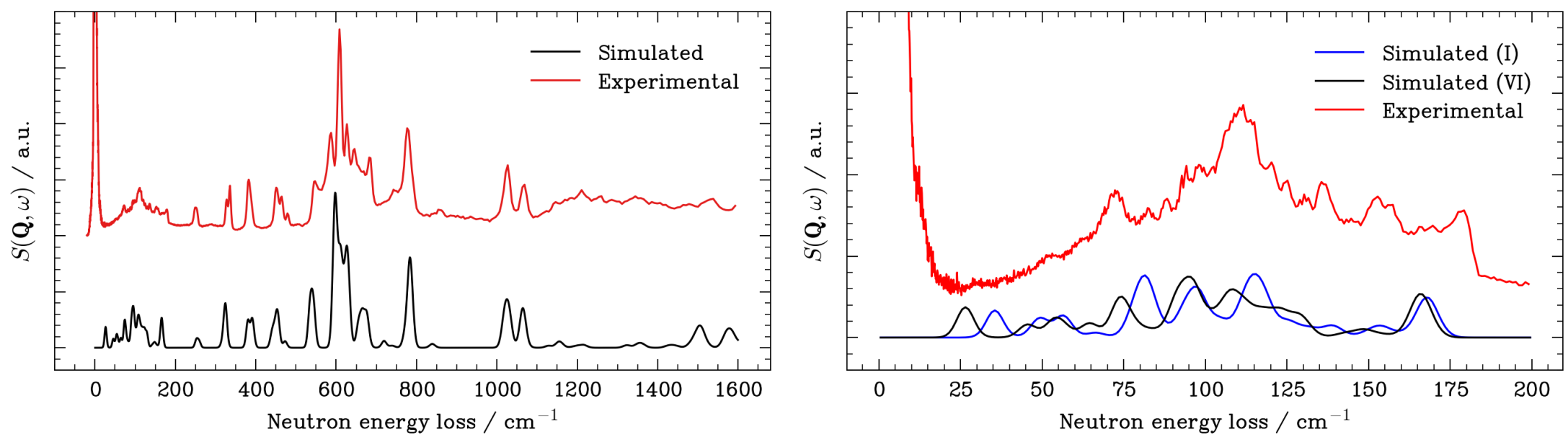

Fig. S2.1. Comparison of simulated and experimental INS spectra for α-**DADNE** at 10 K for: (left) frequencies simulated using the highest fine grid setting (VI, Table S1.1), up to 1600 cm^{-1}, and (right) frequencies simulated using the lowest and the highest grid settings (I and VI, Table S1.1) up to *ca* 200 cm^{-1}. Experimental data was obtained from Ref. 12.

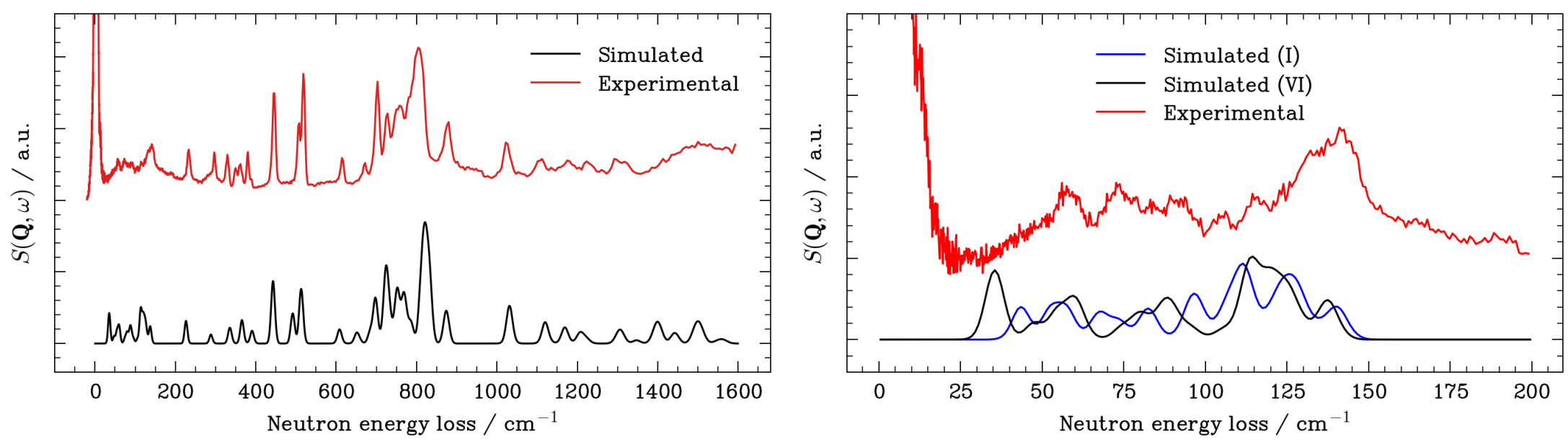

Fig. S2.2. Comparison of simulated and experimental INS spectra for **TATB** at 10 K for: (left) frequencies simulated using the highest fine grid setting (VI, Table S1.1), up to 1600 cm^{-1}, and (right) frequencies simulated using the lowest and the highest grid settings (I and VI, Table S1.1) up to *ca* 200 cm^{-1}. Experimental data was obtained from Ref. 13.

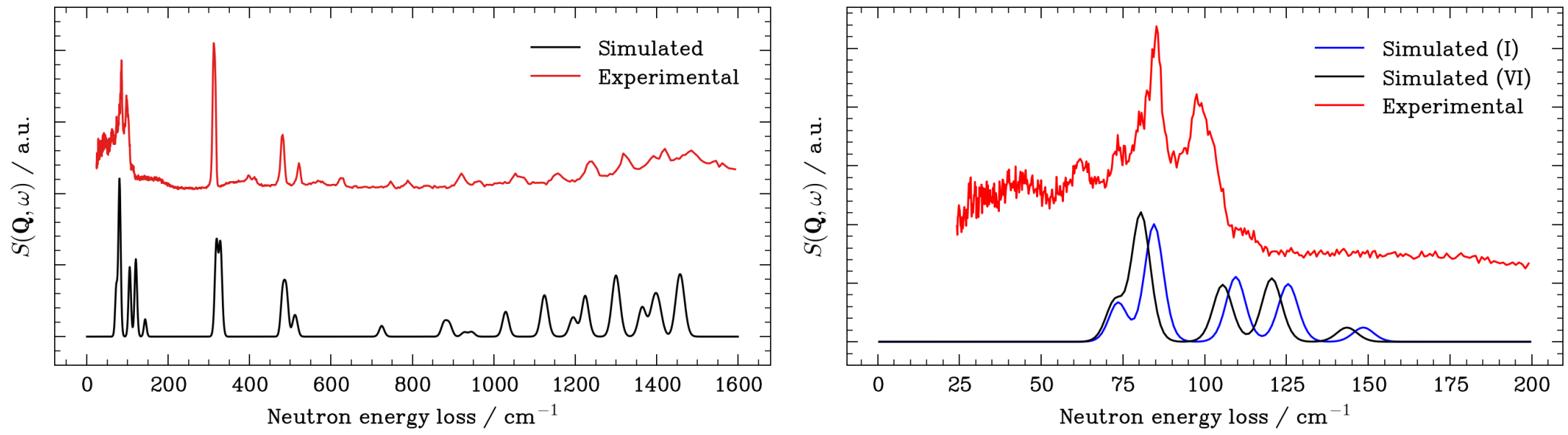

Fig. S2.3. Comparison of simulated and experimental INS spectra for **trioxane** at 10 K for: (left) frequencies simulated using the highest fine grid setting (VI, Table S1.1), up to 1600 cm^{-1}, and (right) frequencies simulated using the lowest and the highest grid settings (I and VI, Table S1.1) up to *ca* 200 cm^{-1}. Experimental data was obtained from Ref. 14.

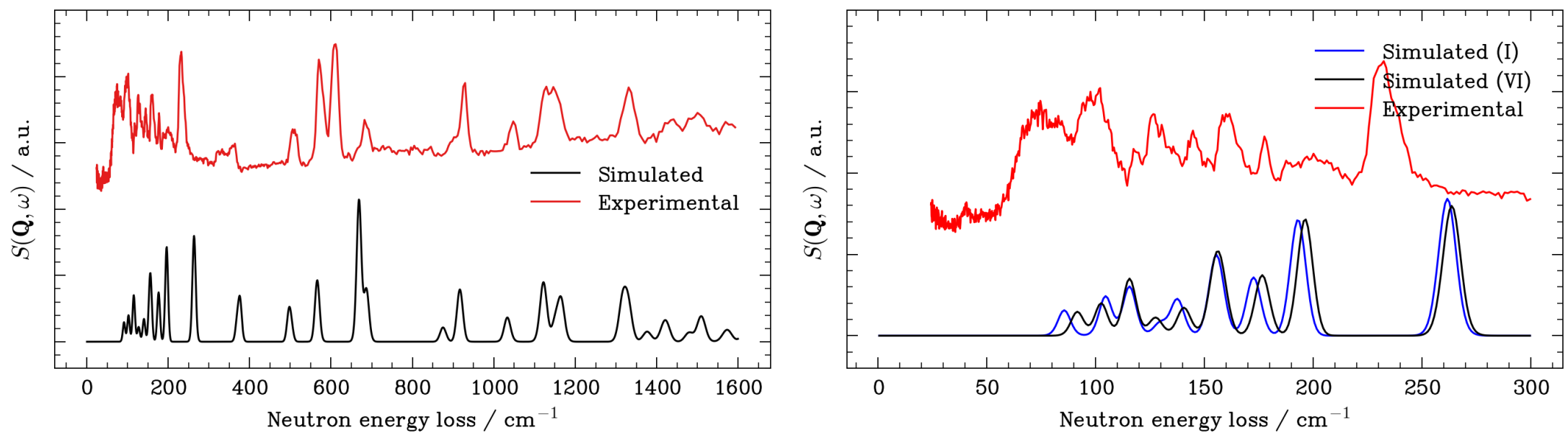

Fig. S2.4. Comparison of simulated and experimental INS spectra for γ-**glycine** at 10 K for: (left) frequencies simulated using the highest fine grid setting (VI, Table S1.1), up to 1600 cm^{-1}, and (right) frequencies simulated using the lowest and the highest grid settings (I and VI, Table S1.1) up to *ca* 300 cm^{-1}. Experimental data was obtained from Ref. 15.

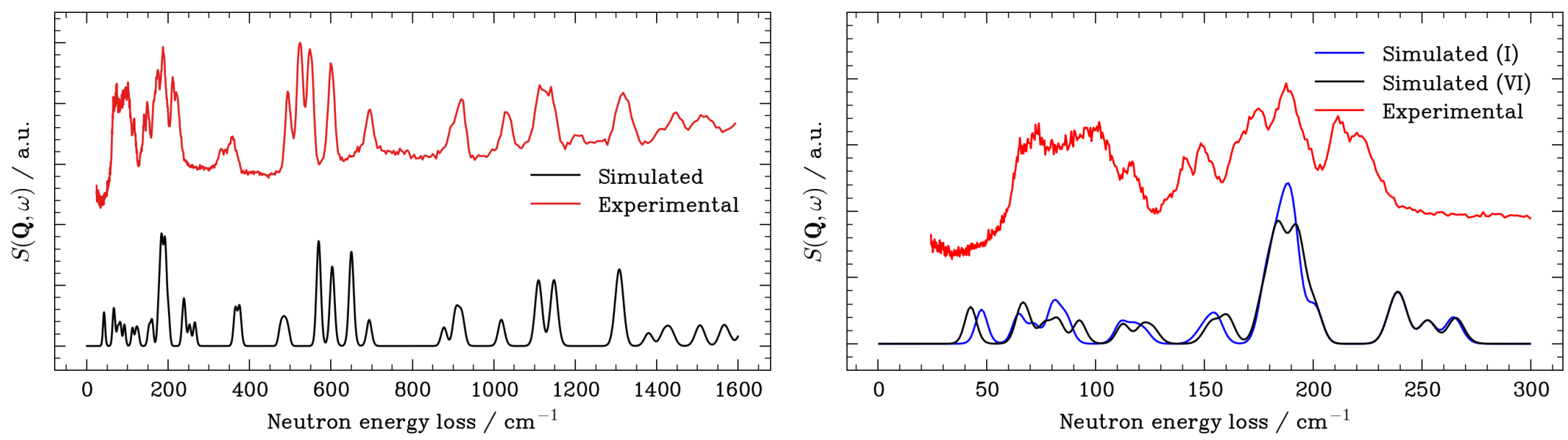

Fig. S2.5. Comparison of simulated and experimental INS spectra for α-**glycine** at 10 K for: (left) frequencies simulated using the highest fine grid setting (VI, Table S1.1), up to 1600 cm^{-1}, and (right) frequencies simulated using the lowest and the highest grid settings (I and VI, Table S1.1) up to *ca* 300 cm^{-1}. Experimental data was obtained from Ref. 15.

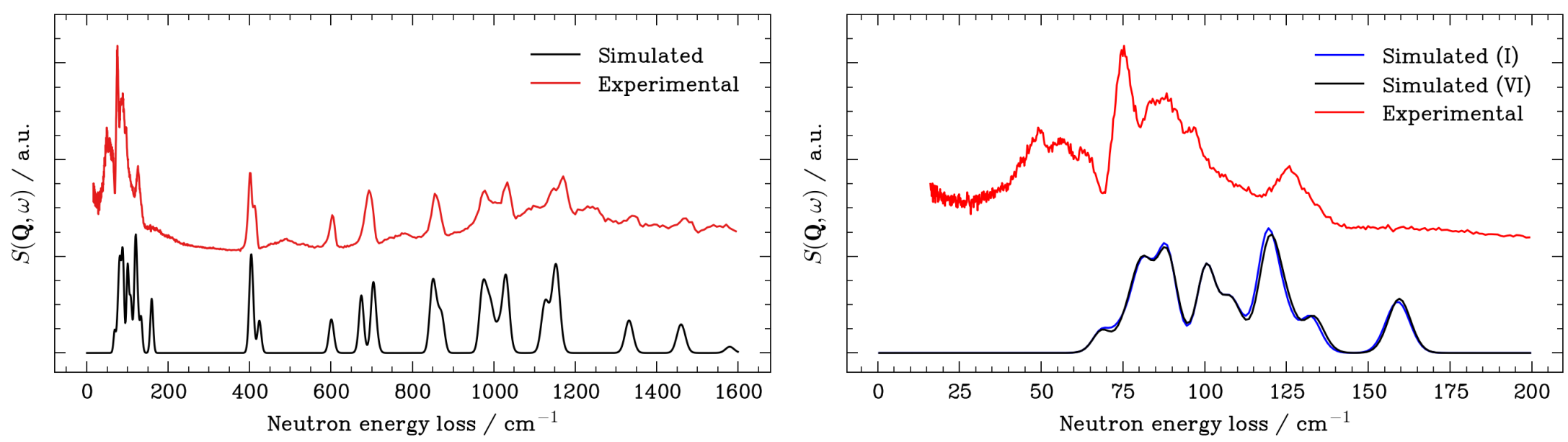

Fig. S2.6. Comparison of simulated and experimental INS spectra for **benzene** at 10 K for: (left) frequencies simulated using the highest fine grid setting (VI, Table S1.1), up to 1600 cm^{-1}, and (right) frequencies simulated using the lowest and the highest grid settings (I and VI, Table S1.1) up to *ca* 200 cm^{-1}. Experimental data was obtained from Ref. 16.

S3 Effect of G_{max}^{fine} on the Γ-point phonon frequencies

To explore the impact of varying G_{max}^{fine} on the Γ-point phonon frequencies in our model OMCs, we compared frequency sets calculated with values of G_{max}^{fine} increasing as described in Table S1.1. We compared the frequencies in the order that maximises the magnitude of the dot product between corresponding pairs of eigenvectors to ensure comparisons are made against equivalent modes, Figures S3.1–S3.12 (left), and as conventionally reported by DFT codes, i.e. directly in ascending order, Figures S3.1–S3.12 (right).

S3.1 Comparison of phonon frequencies calculated with increasing G_{max}^{fine}

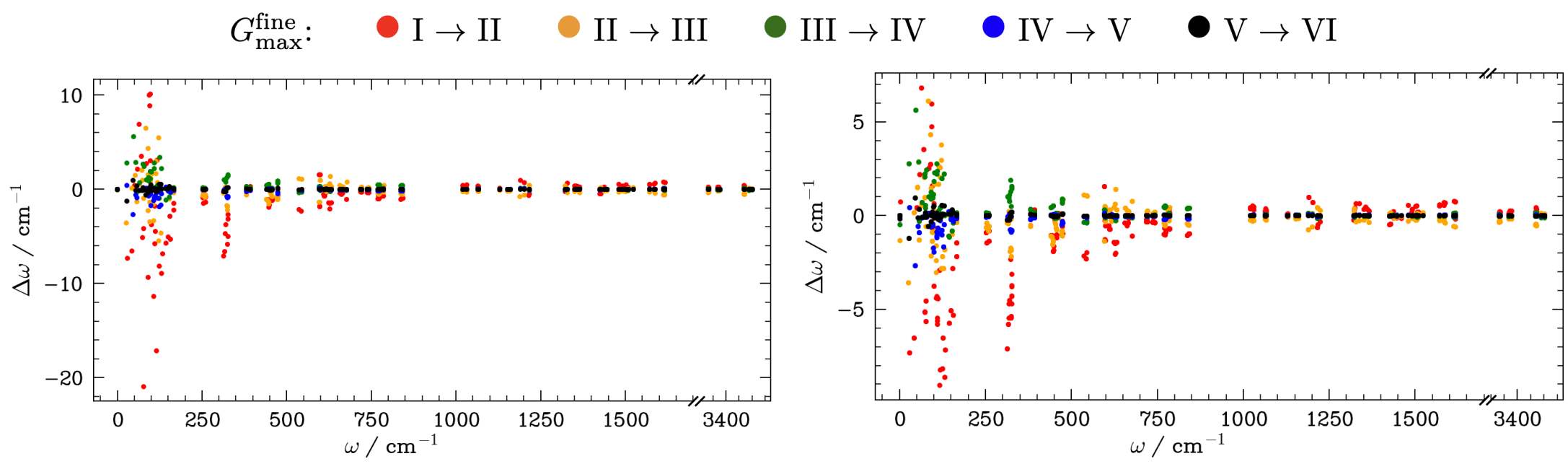

Fig. S3.1. Phonon frequency convergence with respect to increasing G_{max}^{fine} in α-**DADNE**, reported for: (left) frequencies re-ordered to maximise the magnitude of the scalar product between pairs of eigenvectors, and (right) frequencies compared directly in ascending order.

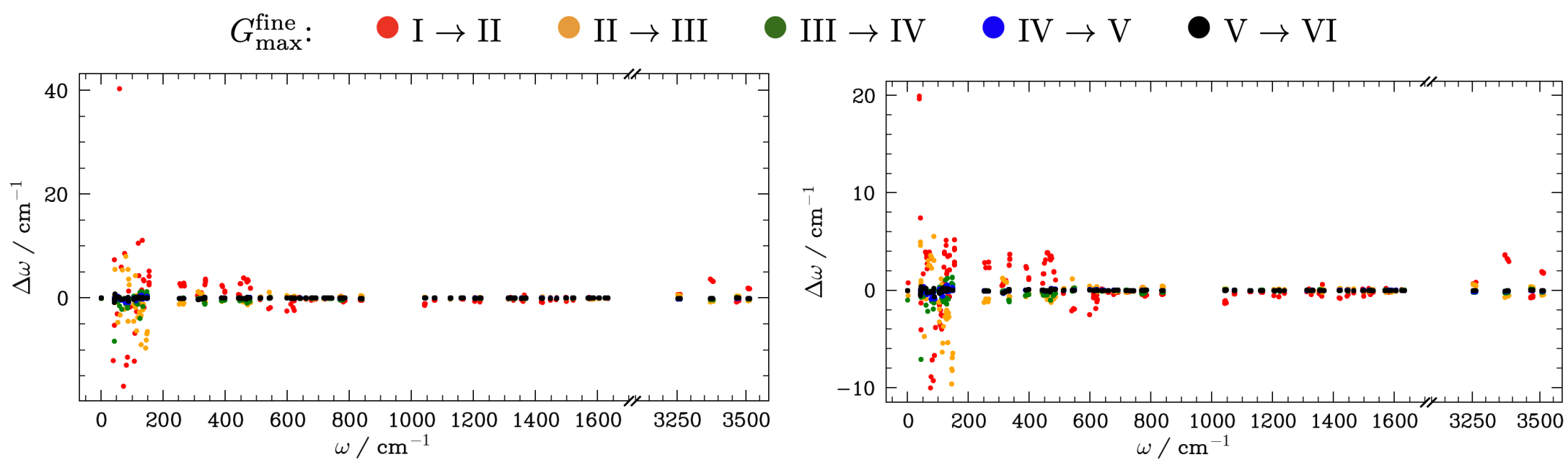

Fig. S3.2. Phonon frequency convergence with respect to increasing G_{max}^{fine} in β-**DADNE**, reported for: (left) frequencies re-ordered to maximise the magnitude of the scalar product between pairs of eigenvectors, and (right) frequencies compared directly in ascending order.

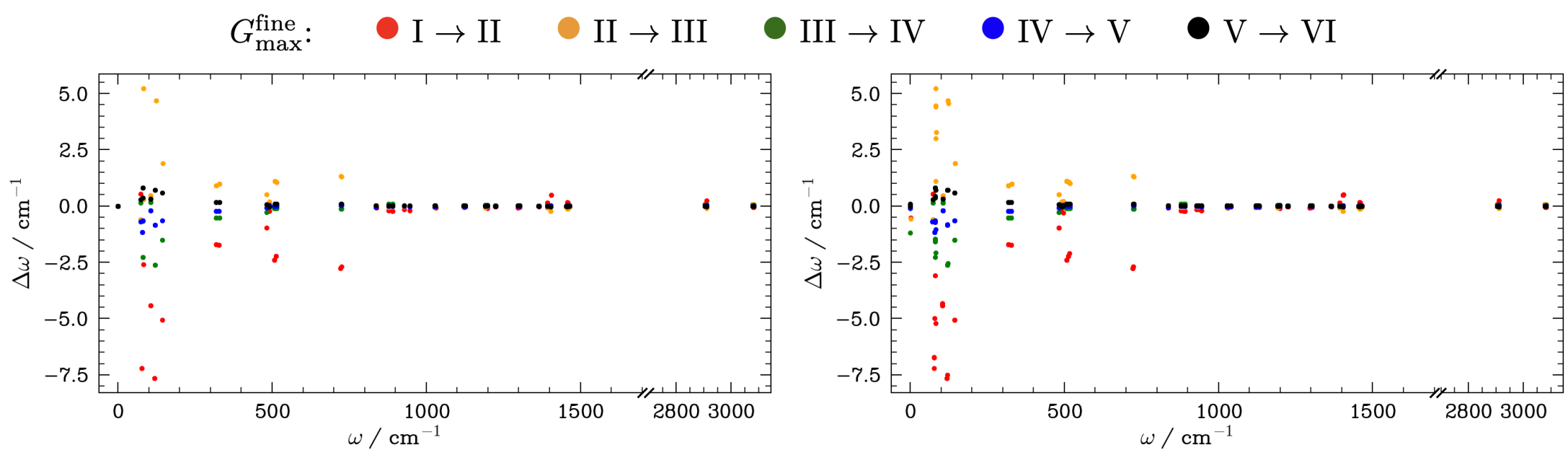

Fig. S3.3. Phonon frequency convergence with respect to increasing G_{max}^{fine} in **trioxane**, reported for: (left) frequencies re-ordered to maximise the magnitude of the scalar product between pairs of eigenvectors, and (right) frequencies compared directly in ascending order.

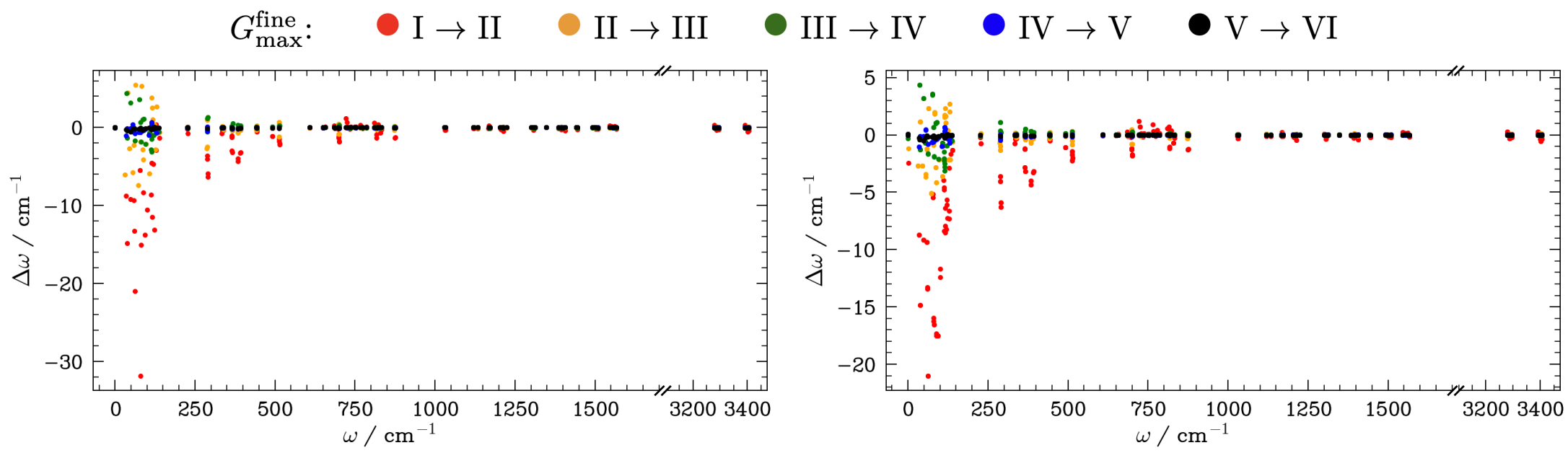

Fig. S3.4. Phonon frequency convergence with respect to increasing G_{max}^{fine} in **TATB**, reported for: (left) frequencies re-ordered to maximise the magnitude of the scalar product between pairs of eigenvectors, and (right) frequencies compared directly in ascending order.

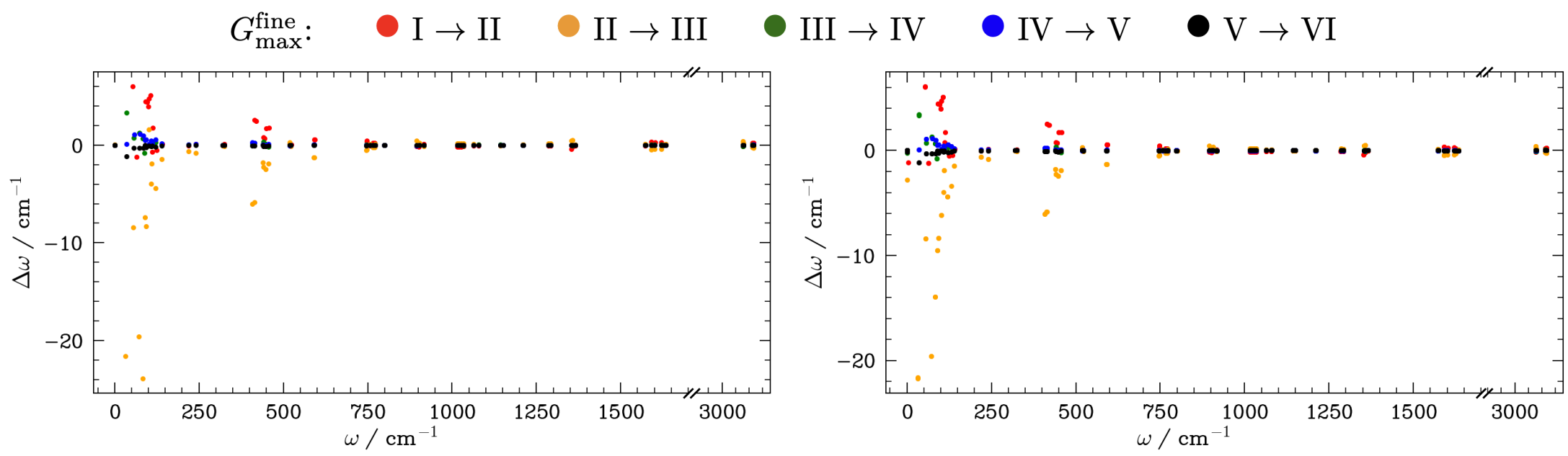

Fig. S3.5. Phonon frequency convergence with respect to increasing G_{max}^{fine} in **benzoquinone**, reported for: (left) frequencies re-ordered to maximise the magnitude of the scalar product between pairs of eigenvectors, and (right) frequencies compared directly in ascending order.

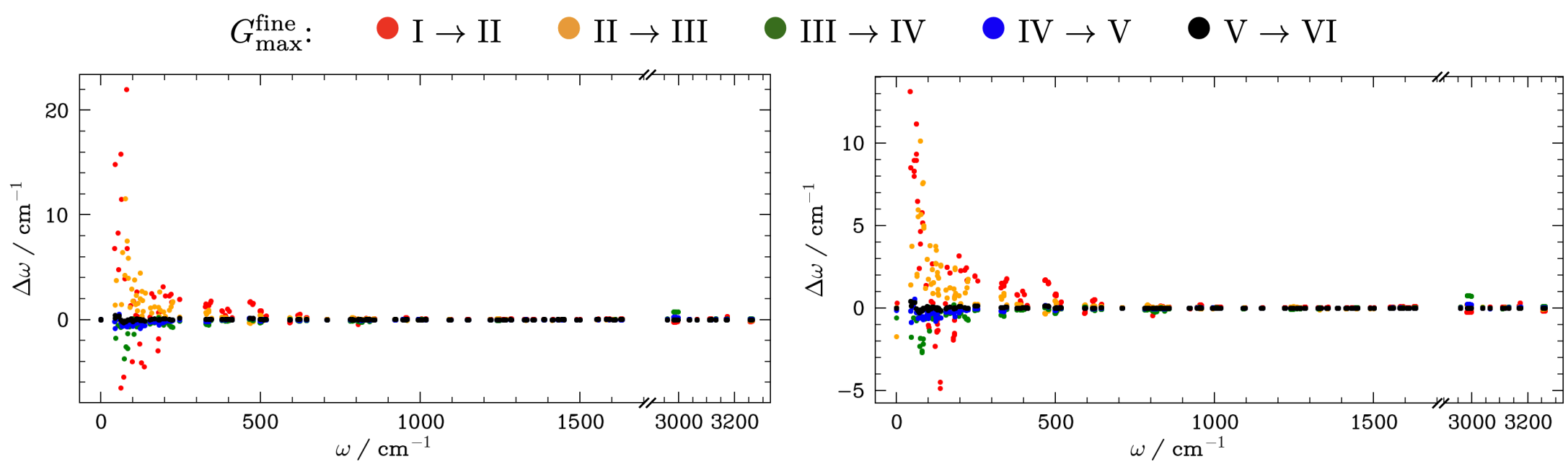

Fig. S3.6. Phonon frequency convergence with respect to increasing G_{max}^{fine} in **paracetamol (Form I)**, reported for: (left) frequencies re-ordered to maximise the magnitude of the scalar product between pairs of eigenvectors, and (right) frequencies compared directly in ascending order.

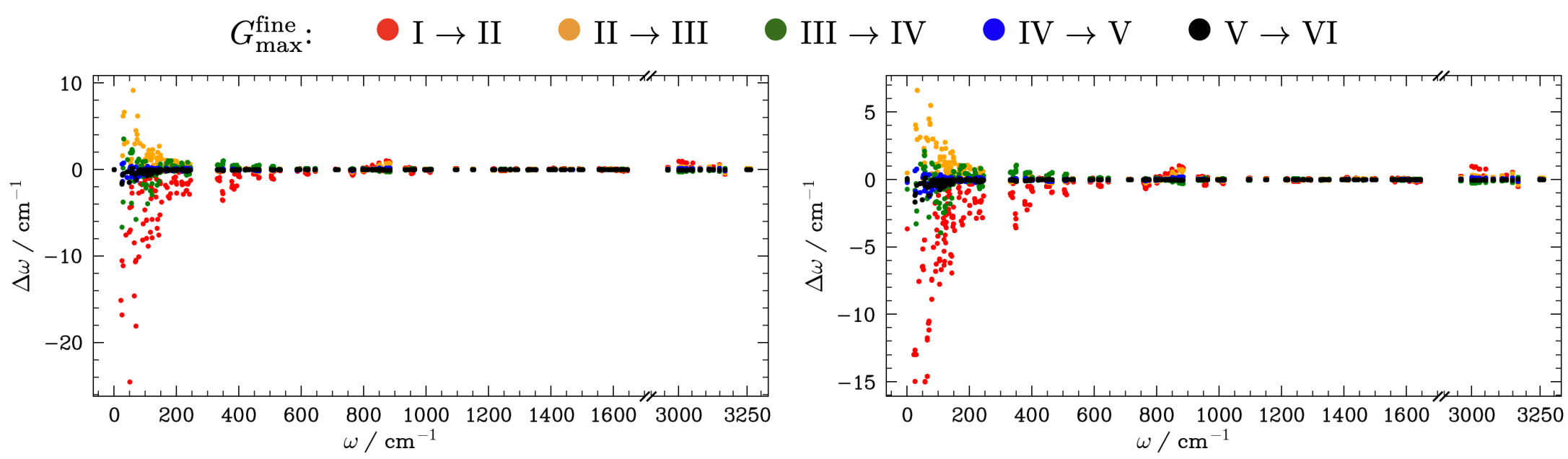

Fig. S3.7. Phonon frequency convergence with respect to increasing G_{max}^{fine} in **paracetamol (Form II)**, reported for: (left) frequencies re-ordered to maximise the magnitude of the scalar product between pairs of eigenvectors, and (right) frequencies compared directly in ascending order.

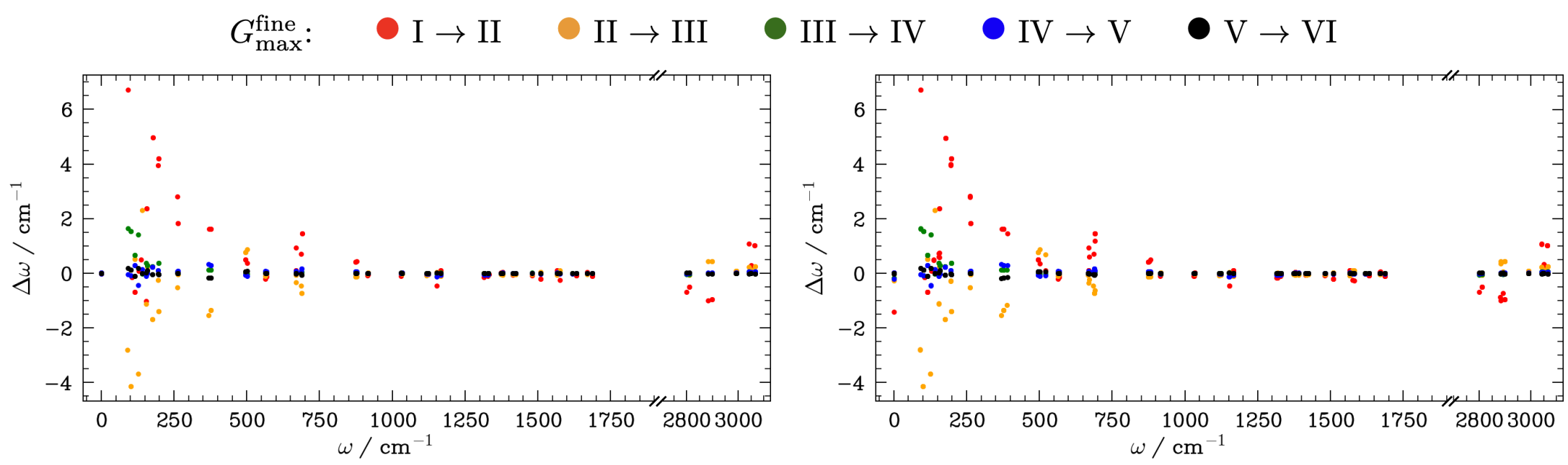

Fig. S3.8. Phonon frequency convergence with respect to increasing $G_{\mathrm{max}}^{\mathrm{fine}}$ in γ-**glycine**, reported for: (left) frequencies re-ordered to maximise the magnitude of the scalar product between pairs of eigenvectors, and (right) frequencies compared directly in ascending order.

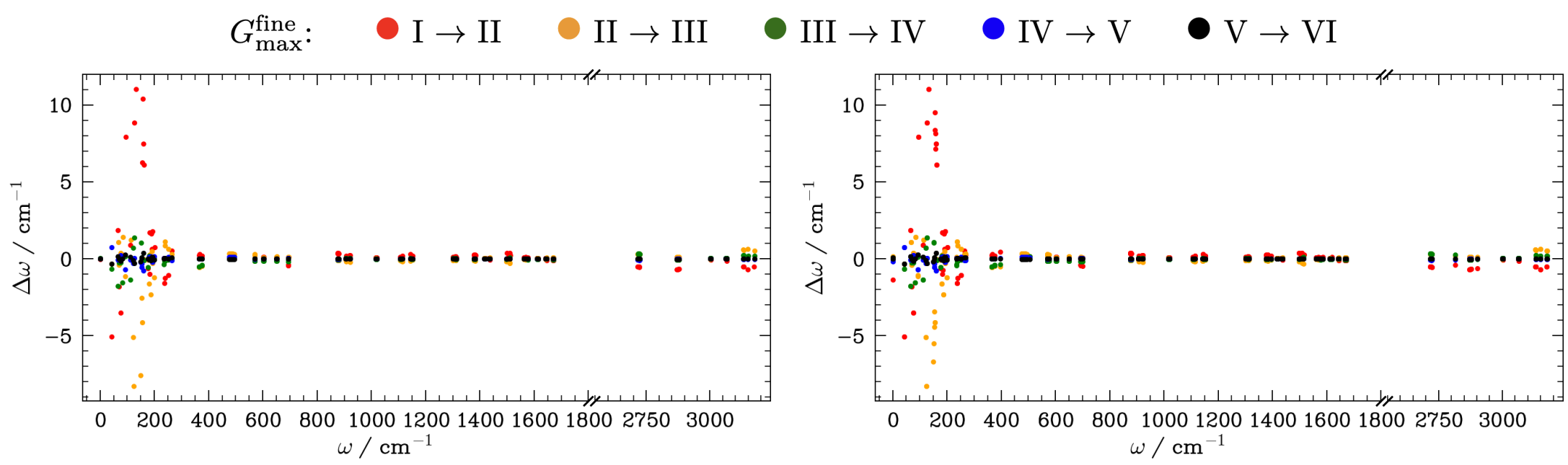

Fig. S3.9. Phonon frequency convergence with respect to increasing $G_{\mathrm{max}}^{\mathrm{fine}}$ in α-**glycine**, reported for: (left) frequencies re-ordered to maximise the magnitude of the scalar product between pairs of eigenvectors, and (right) frequencies compared directly in ascending order.

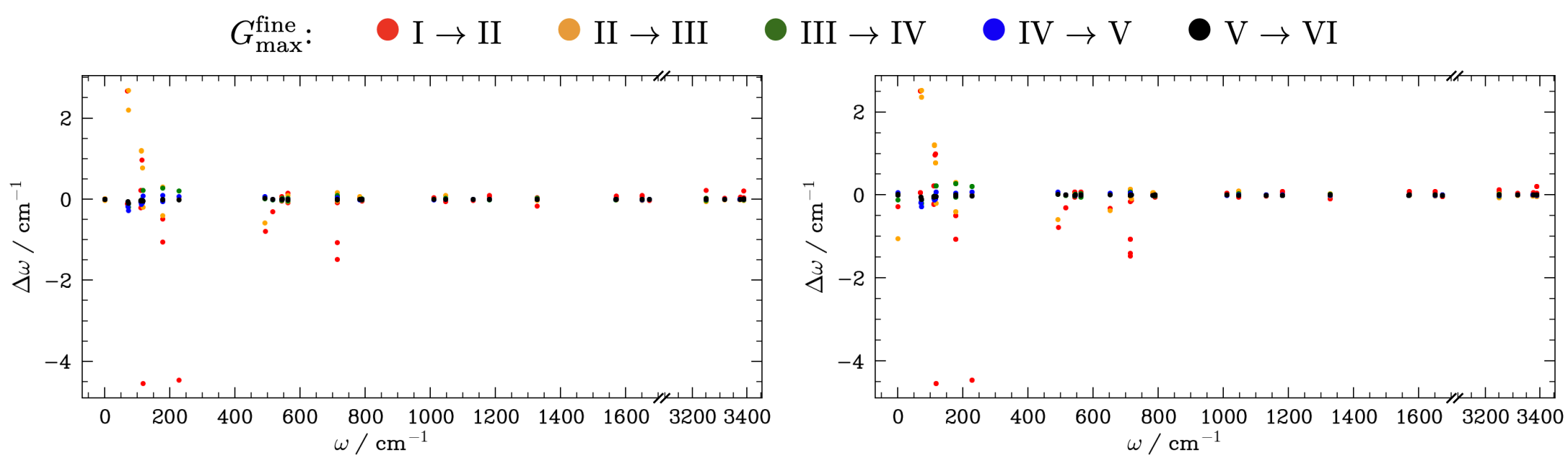

Fig. S3.10. Phonon frequency convergence with respect to increasing $G_{\mathrm{max}}^{\mathrm{fine}}$ in **guanidinium nitrate**, reported for: (left) frequencies re-ordered to maximise the magnitude of the scalar product between pairs of eigenvectors, and (right) frequencies compared directly in ascending order.

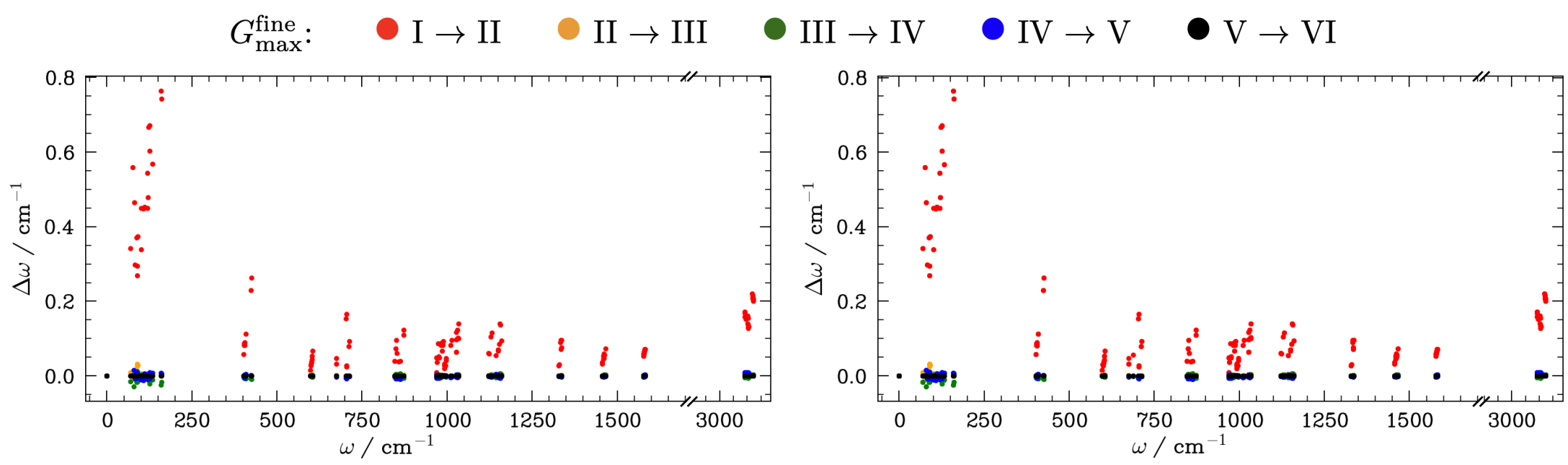

Fig. S3.11. Phonon frequency convergence with respect to increasing $G_{\mathrm{max}}^{\mathrm{fine}}$ in **benzene**, reported for: (left) frequencies re-ordered to maximise the magnitude of the scalar product between pairs of eigenvectors, and (right) frequencies compared directly in ascending order.

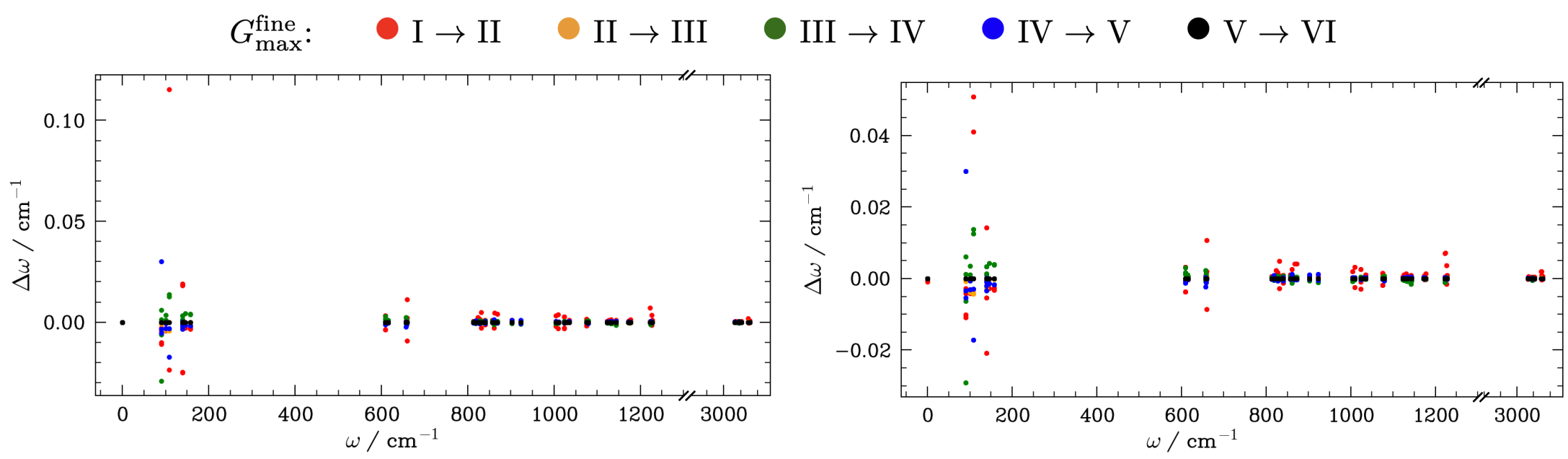

Fig. S3.12. Phonon frequency convergence with respect to increasing $G_{\rm max}^{\rm fine}$ in **cubane**, reported for: (left) frequencies re-ordered to maximise the magnitude of the scalar product between pairs of eigenvectors, and (right) frequencies compared directly in ascending order.

S3.2 Summary of frequency convergence to < 0.5 cm^{-1}, < 2 cm^{-1} and 5 cm^{-1} with respect to increasing $G_{\text{max}}^{\text{fine}}$

The convergence behaviour Figures S3.1–S3.12 is summarised in Figure S3.13 by marking which frequencies are converged to within (a) < 0.5 cm^{-1}, (b) < 2 cm^{-1} and (c) < 5 cm^{-1} as $G_{\text{max}}^{\text{fine}}$ is increased as described in Table S1.1. Clearly, CHNO and CHO systems containing harder pseudised atomic cores (oxygen and nitrogen) require significantly larger $G_{\text{max}}^{\text{fine}}$ than CH systems to ensure the convergence of frequencies at each threshold.

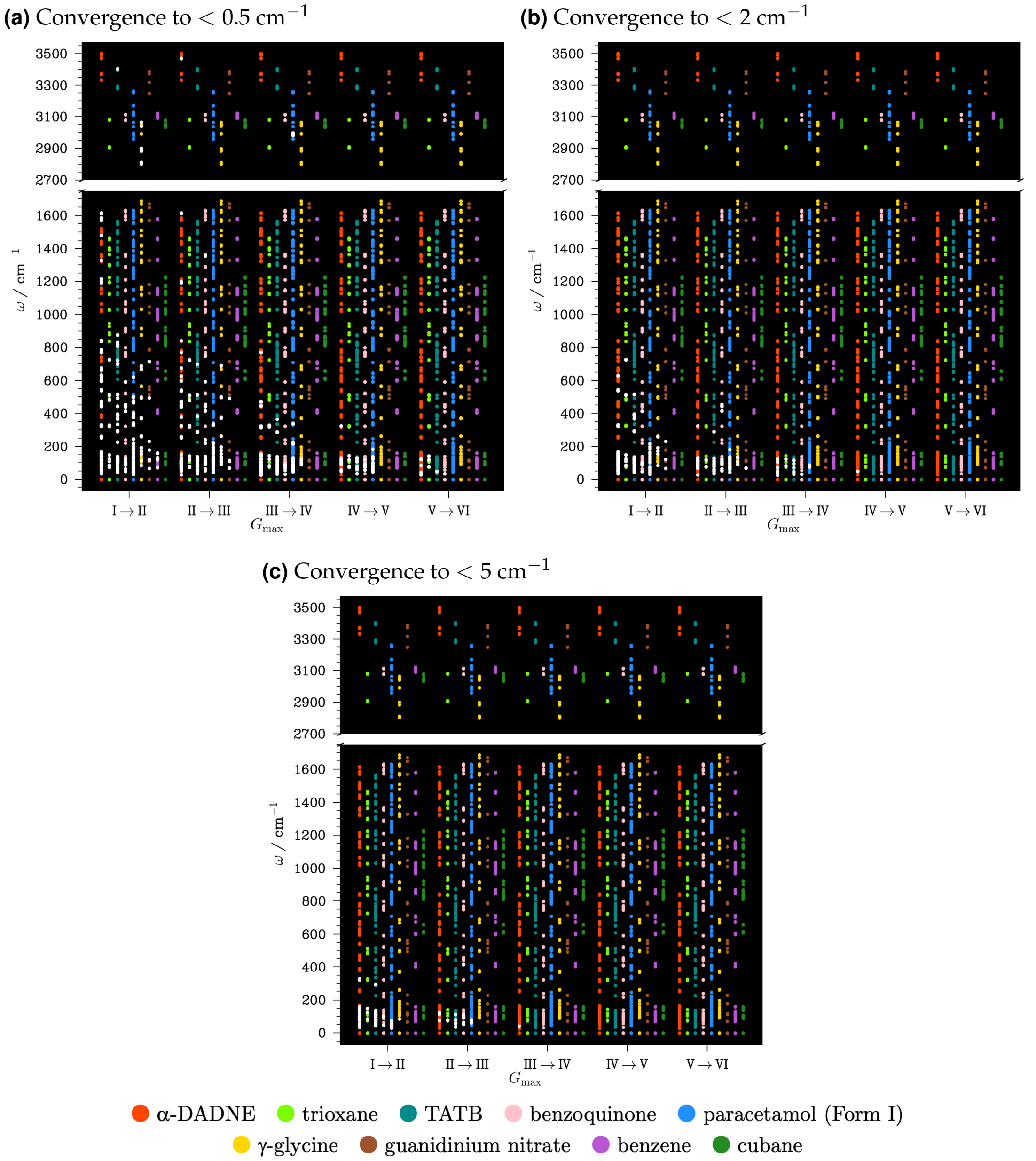

Fig. S3.13. Summary of phonon frequency convergence to (a) < 0.5 cm^{-1}, (b) < 2 cm^{-1} and (c) 5 cm^{-1} with respect to increasing $G_{\text{max}}^{\text{fine}}$. Unconverged frequencies are shown in white, with converged frequencies displayed in colour. Frequencies were compared in the order that maximises the magnitude of the scalar product between pairs of eigenvectors.

S3.3 Comparison of phonon density of states calculated with increasing G_{max}^{fine}

To compare the effect that G_{max}^{fine} has on the simulated phonon frequencies, we compare the phonon density of states (pDOS) for each of our model OMCs as calculated with the G_{max}^{fine} values tabulated in Table S1.1. The resulting pDOS are shown for the range $0 < \omega < 500$ cm^{-1} for each OMC in Figures S3.14–S3.21. There is a clear effect of increasing G_{max}^{fine} from a scale of I to II across the entire frequency range. As the scaling parameters increase beyond II, the most obvious effects are seen in the lowest frequencies. This is extremely evident for $\omega < 50$ cm^{-1} in all cases. These simulations suggest that any study of lattice vibrations in OMCs *must* ensure careful convergence of G_{max}^{fine}.

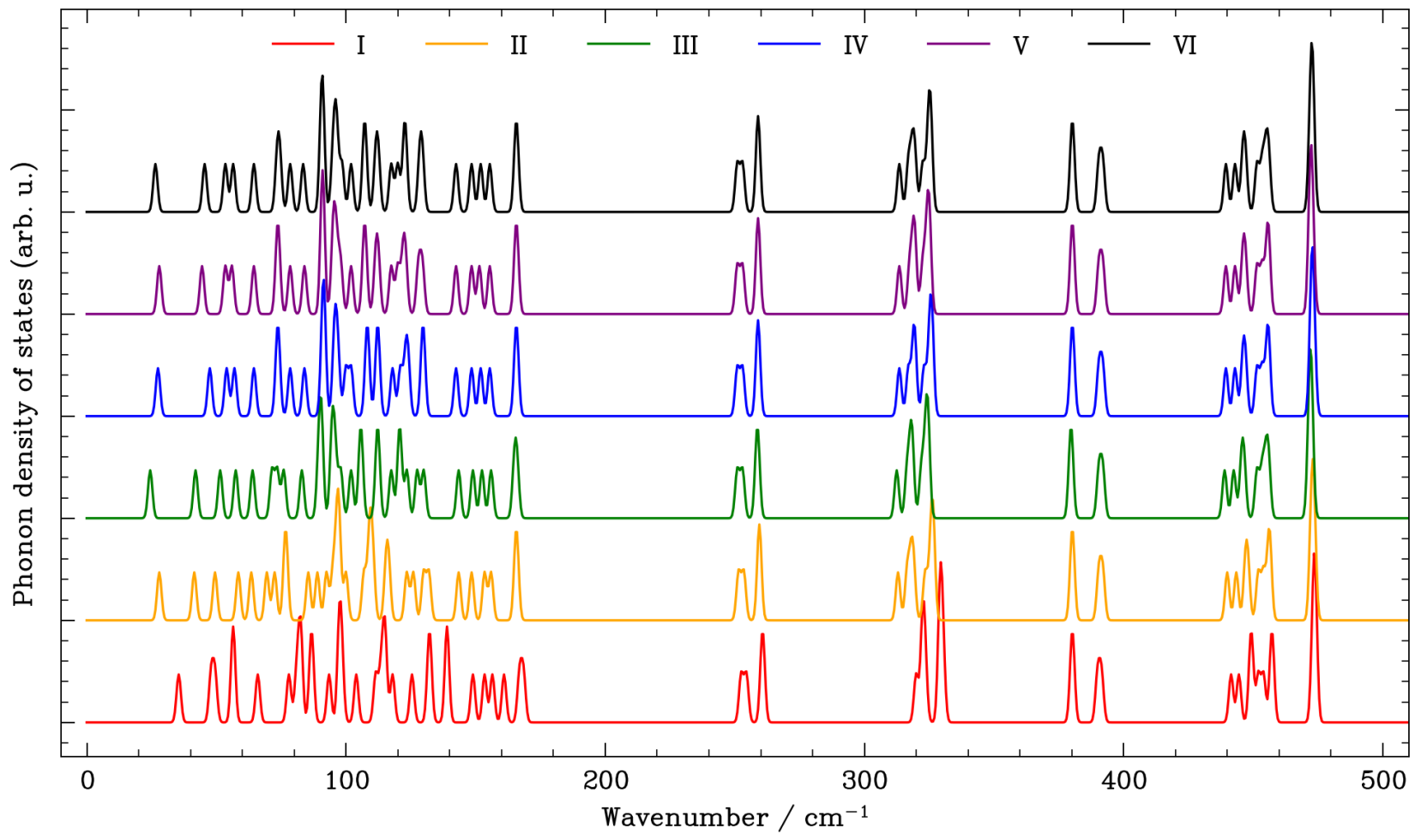

Fig. S3.14. Simulated phonon density of states up to 500 cm^{-1} using phonon frequencies calculated with G_{max}^{fine} varied according to Table S1.1 for α-**DADNE**.

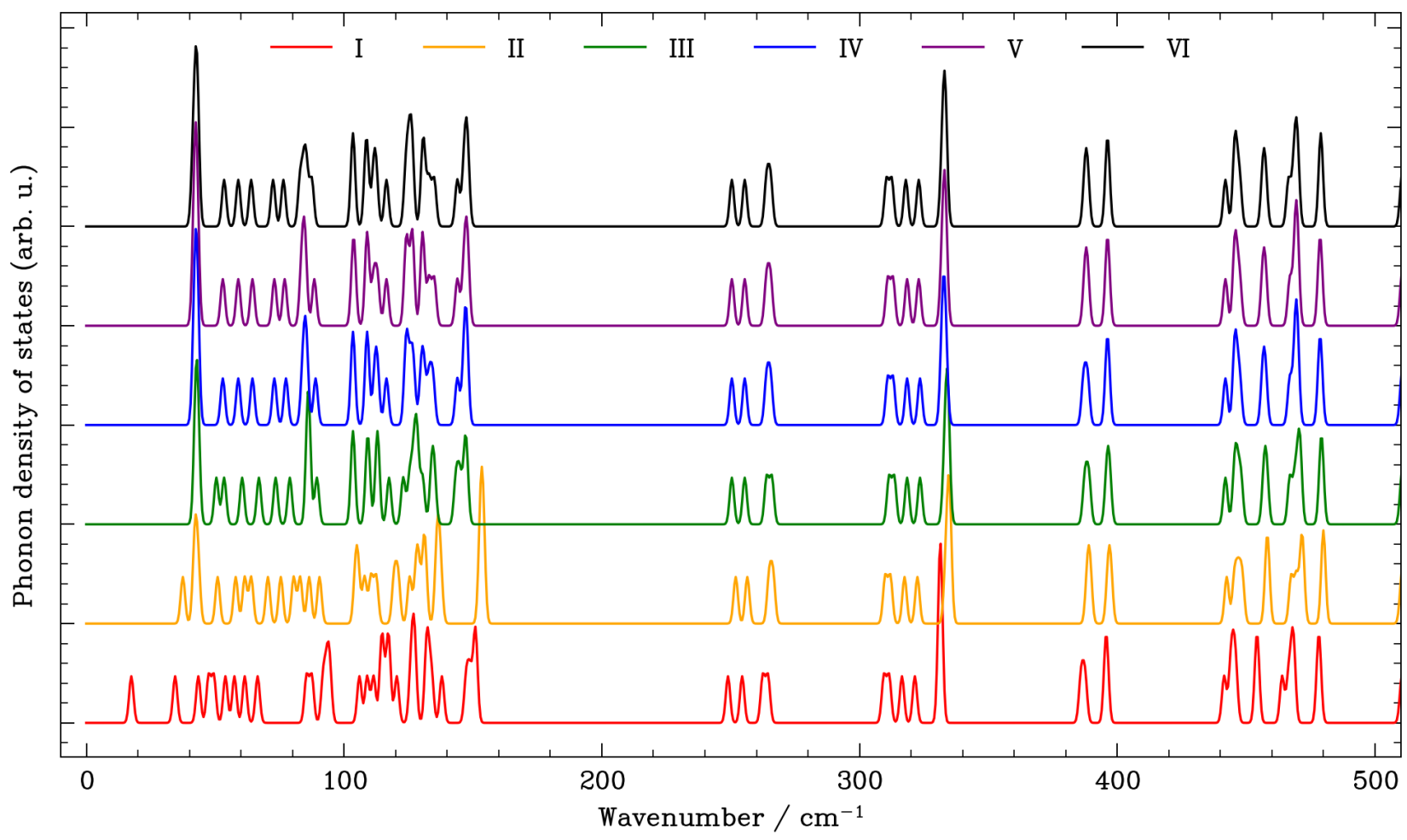

Fig. S3.15. Simulated phonon density of states up to 500 cm^{-1} using phonon frequencies calculated with G_{max}^{fine} varied according to Table S1.1 for β-**DADNE**.

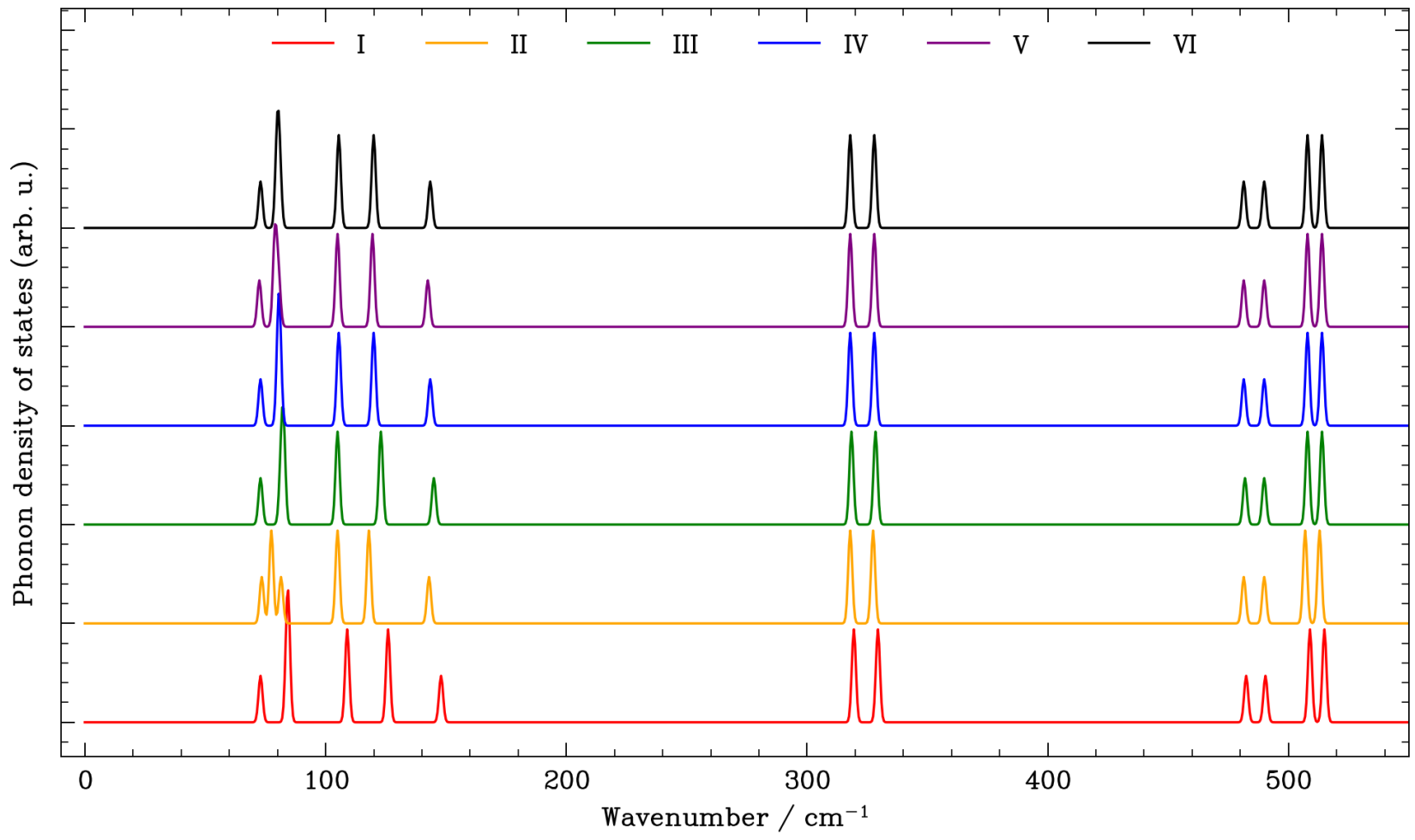

Fig. S3.16. Simulated phonon density of states up to 500 cm^{-1} using phonon frequencies calculated with G_{max}^{fine} varied according to Table S1.1 for **trioxane**.

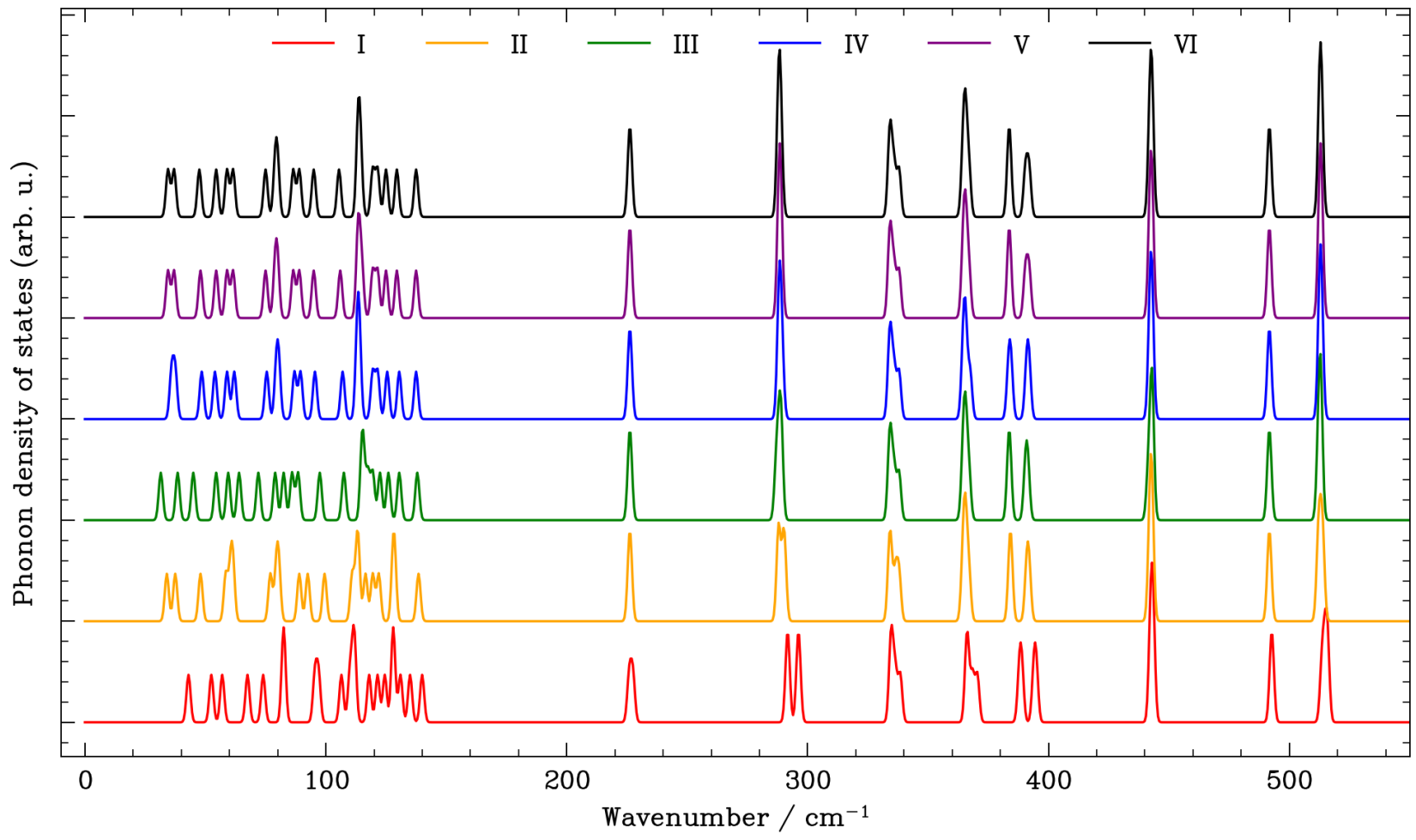

Fig. S3.17. Simulated phonon density of states up to 500 cm^{-1} using phonon frequencies calculated with G_{max}^{fine} varied according to Table S1.1 for **TATB**.

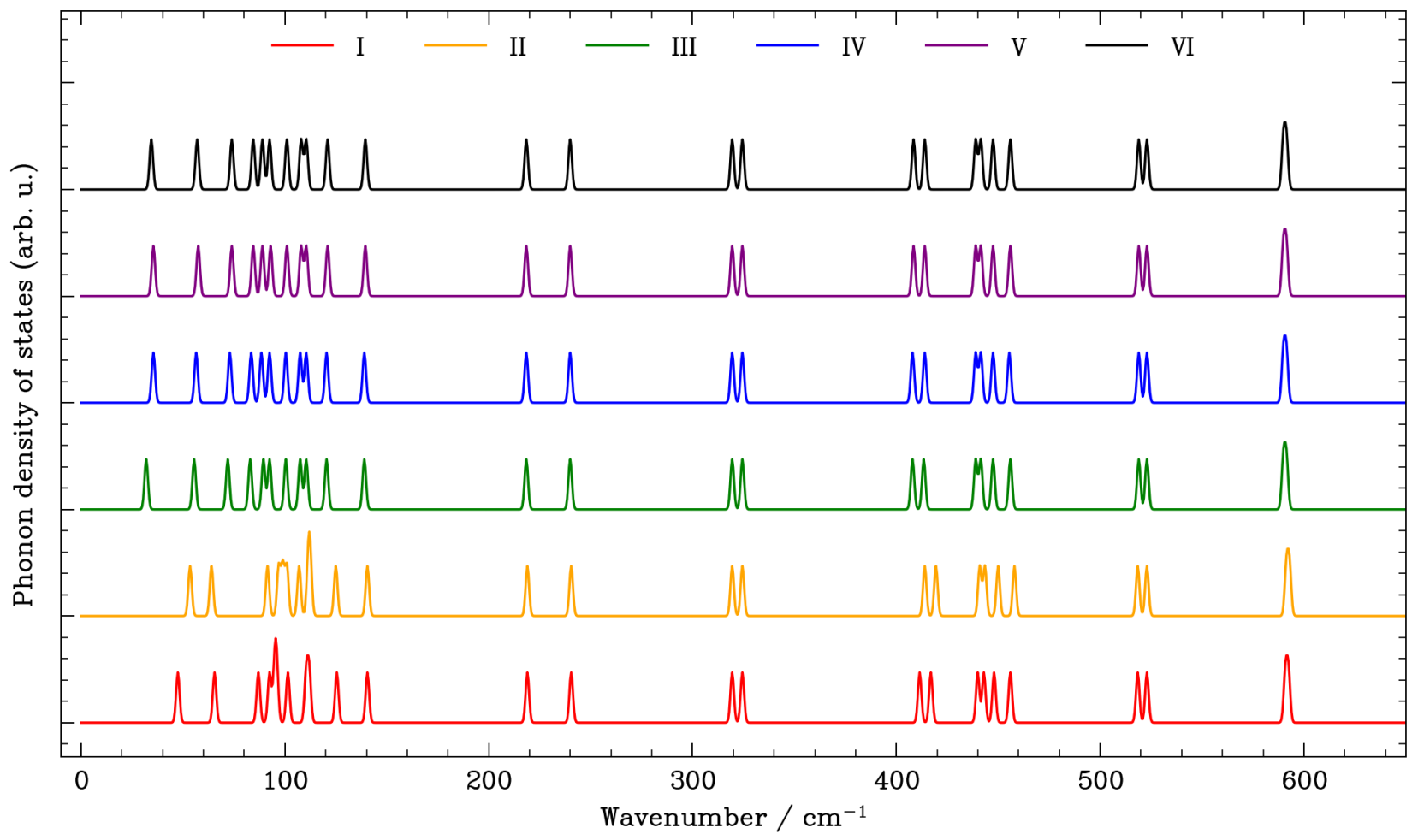

Fig. S3.18. Simulated phonon density of states up to 600 cm^{-1} using phonon frequencies calculated with G_{max}^{fine} varied according to Table S1.1 for **benzoquinone**.

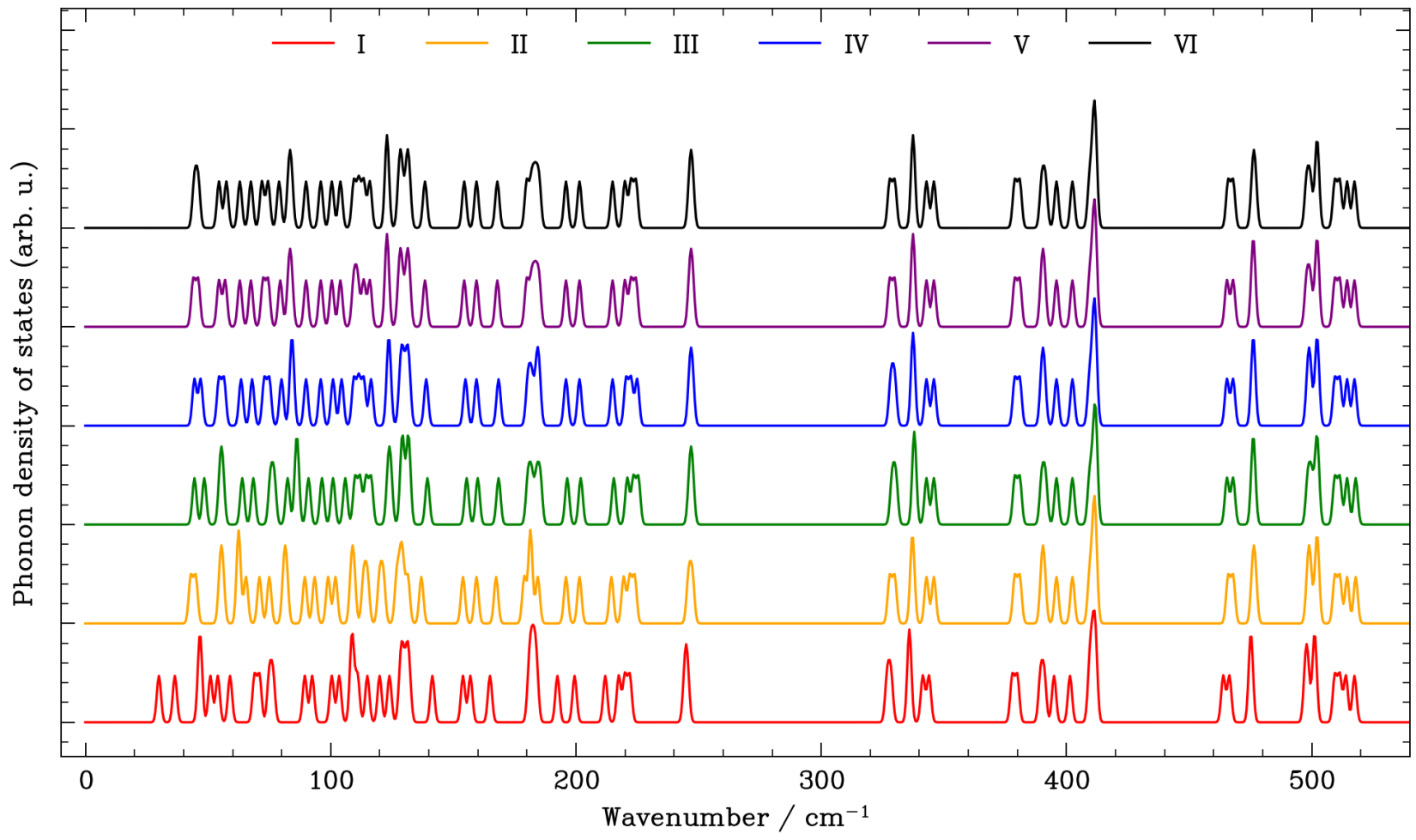

Fig. S3.19. Simulated phonon density of states up to 500 cm^{-1} using phonon frequencies calculated with $G_{\text{max}}^{\text{fine}}$ varied according to Table S1.1 for **paracetamol (Form I)**.

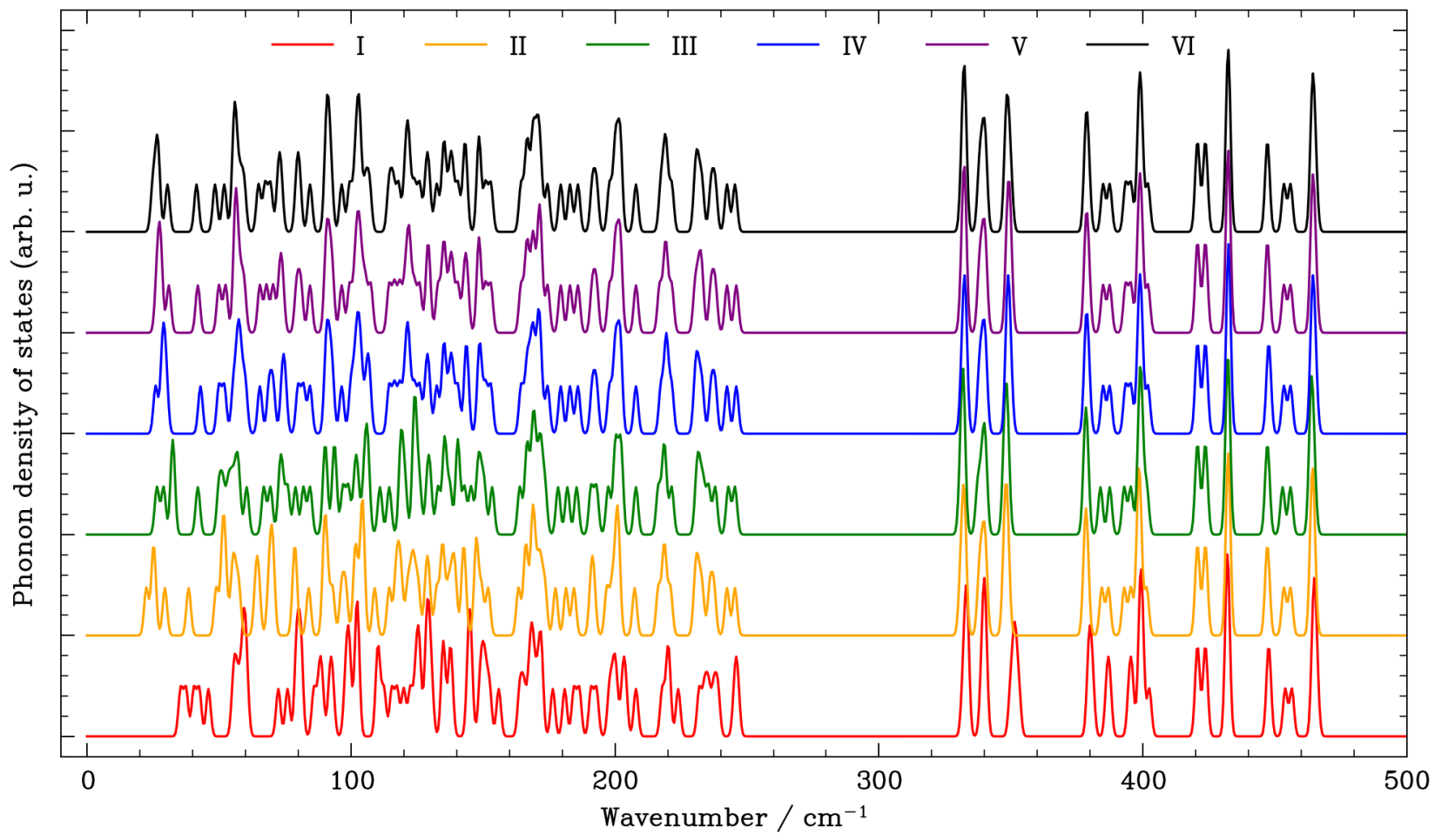

Fig. S3.20. Simulated phonon density of states up to 500 cm^{-1} using phonon frequencies calculated with $G_{\text{max}}^{\text{fine}}$ varied according to Table S1.1 for **paracetamol (Form II)**.

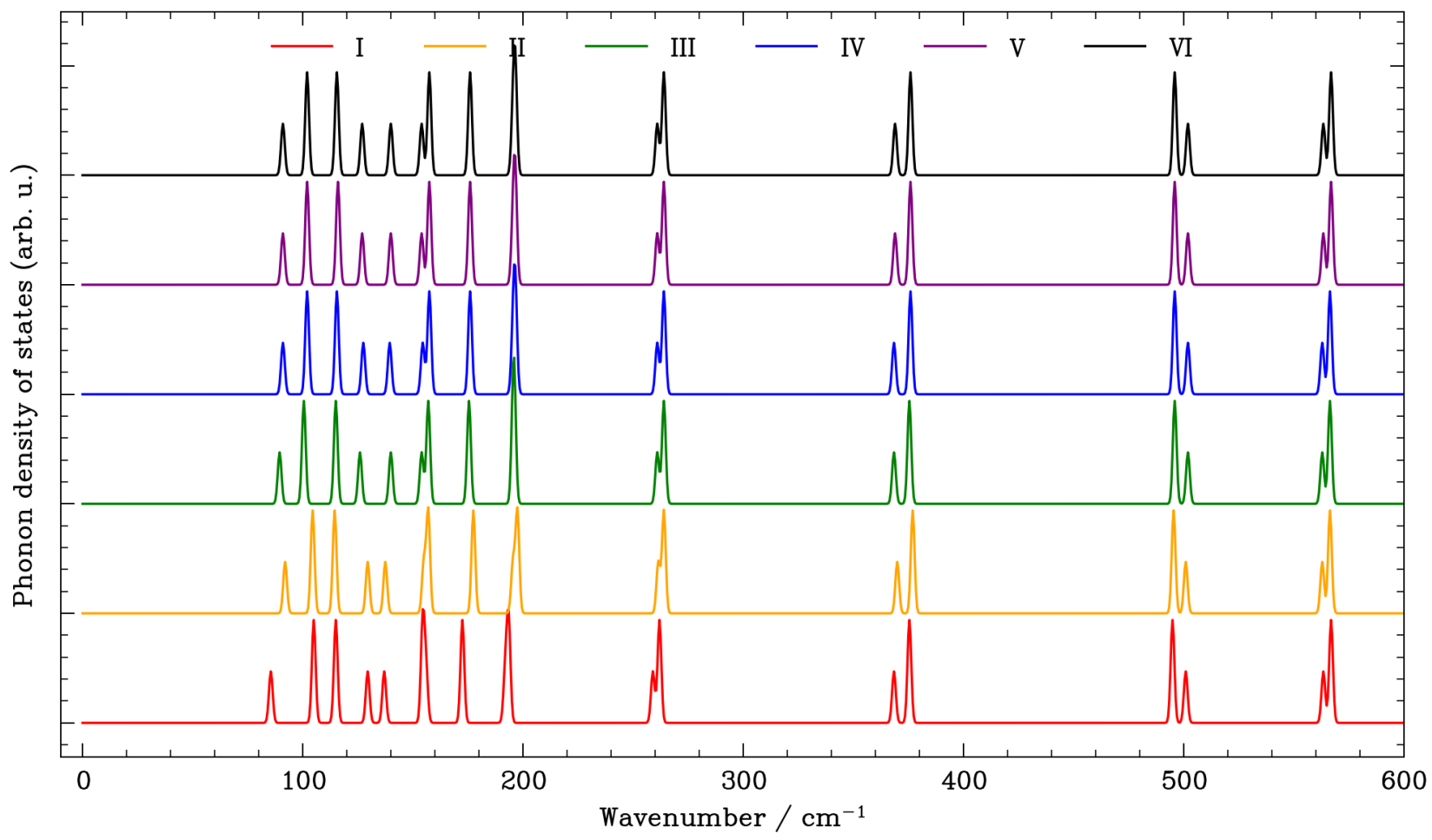

Fig. S3.21. Simulated phonon density of states up to 600 cm^{-1} using phonon frequencies calculated with $G_{\text{max}}^{\text{fine}}$ varied according to Table S1.1 for γ-**glycine**.

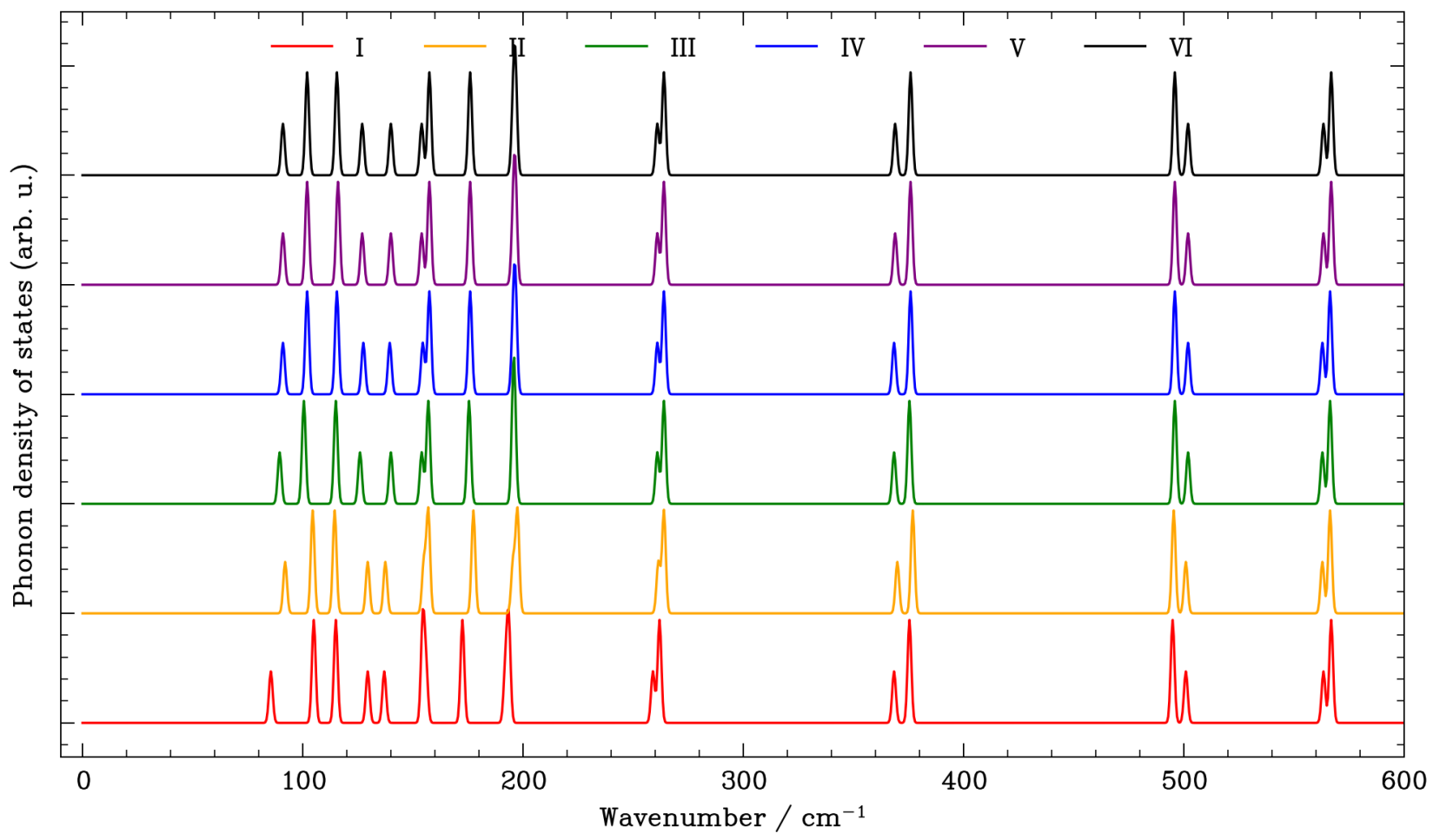

Fig. S3.22. Simulated phonon density of states up to 600 cm^{-1} using phonon frequencies calculated with G_{max}^{fine} varied according to Table S1.1 for α-**glycine**.

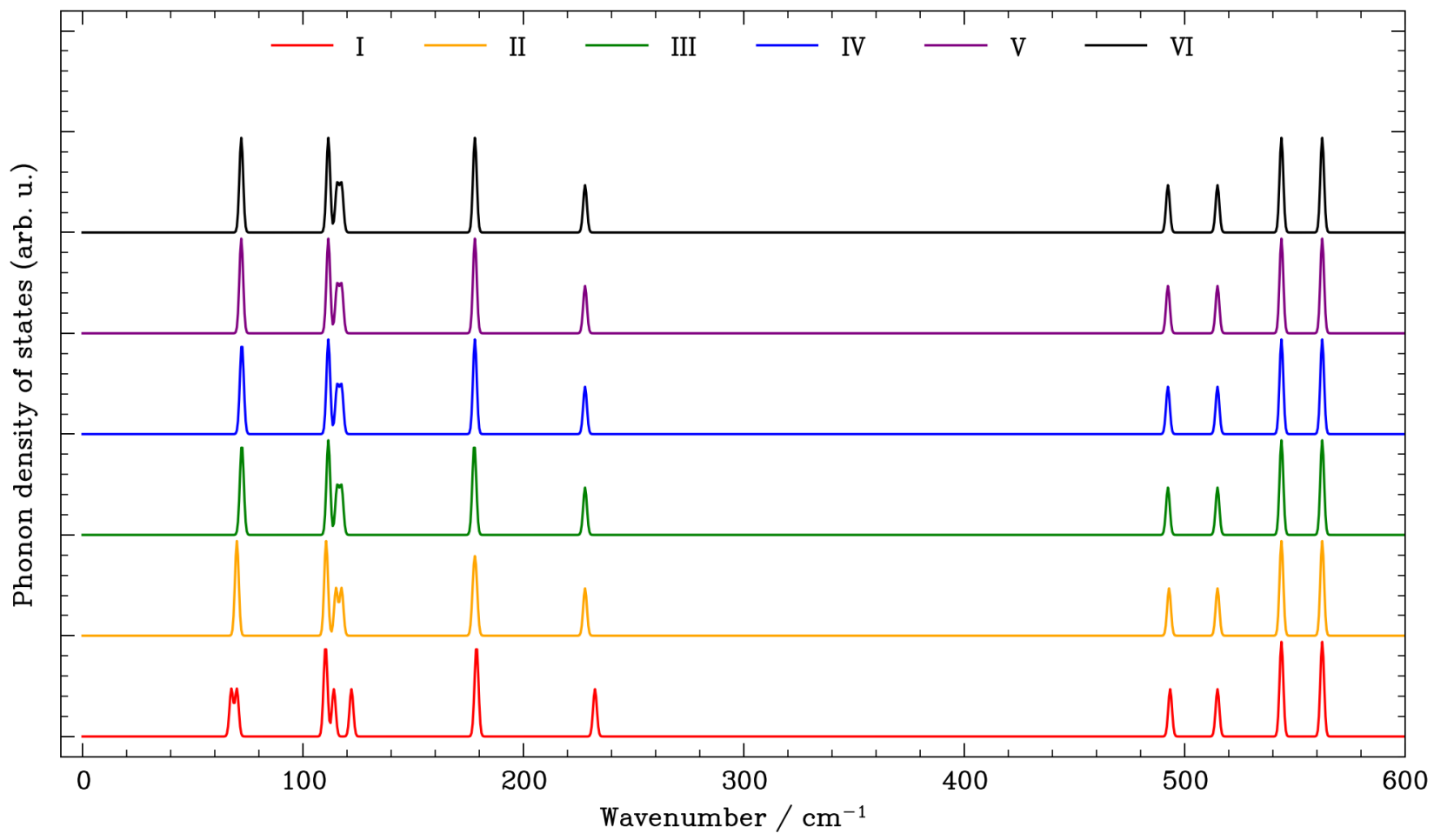

Fig. S3.23. Simulated phonon density of states up to 600 cm^{-1} using phonon frequencies calculated with G_{max}^{fine} varied according to Table S1.1 for **guanidinium nitrate**.

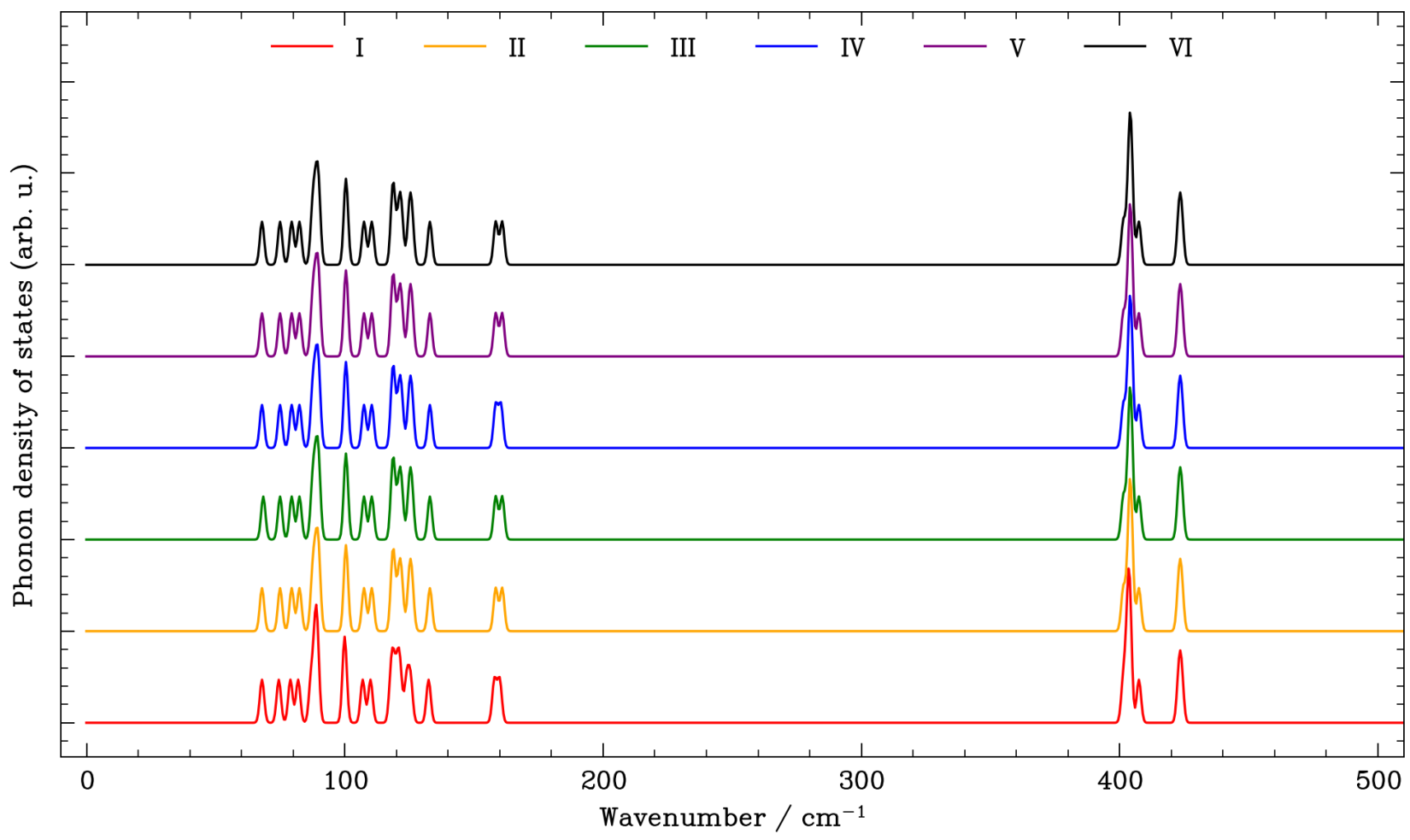

Fig. S3.24. Simulated phonon density of states using phonon frequencies calculated with G_{max}^{fine} varied according to Table S1.1 for **benzene**.

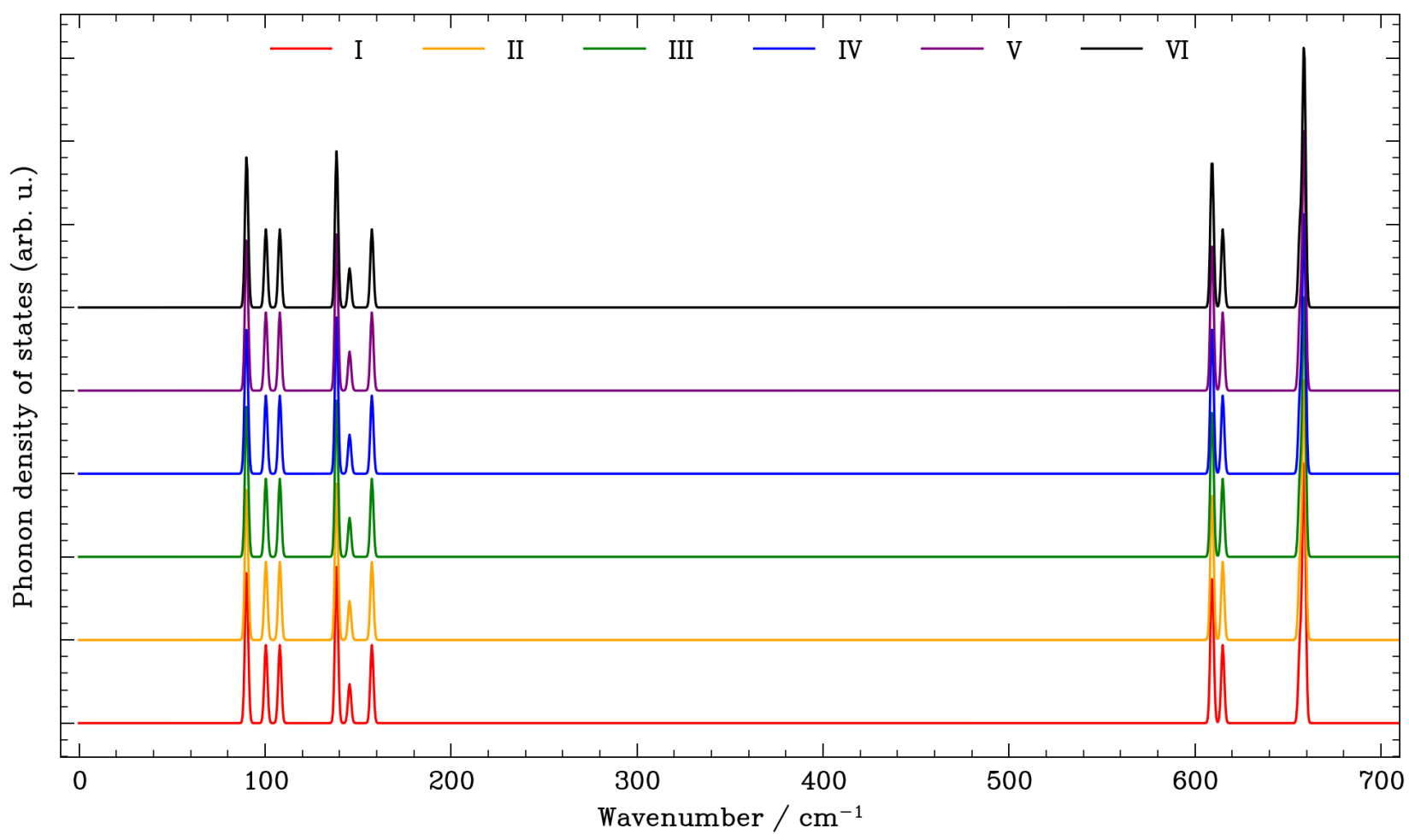

Fig. S3.25. Simulated phonon density of states up to 700 cm^{-1} using phonon frequencies calculated with G_{max}^{fine} varied according to Table S1.1 for **cubane**.

S4 Thermodynamic data

Using the phonon frequencies calculated for increasing $G^{\text{fine}}_{\text{max}}$ (see Section S2), we calculate the vibrational contributions to the free energy, F_{vib} and associated vibrational heat capacities. These are reported for our model OMCs in Tables S4.2–S4.12. Despite significant changes in the overall phonon frequencies, we observe only minor changes ($<$ 0.1 eV in the extreme case of DADNE and 0 eV for the case of cubane) in the predicted F_{vib} as we increase $G^{\text{fine}}_{\text{max}}$. Hence, we generally expect that $G^{\text{fine}}_{\text{max}}$ will have negligible impact on the thermodynamic quantities calculated for OMCs where all other convergence criteria are sufficiently converged.

Table S4.1. Thermodynamic data for the unit cell of α-**DADNE**, calculated at 300 K.

Fine Grid	ZPE / eV	F_{vib} / eV	C_v / J mol^{-1} K^{-1}
2	9.943	8.922	620.610
3	9.934	8.891	621.587
4	9.932	8.889	621.756
5	9.935	8.904	621.516
6	9.934	8.899	621.625
7	9.934	8.899	621.613

Table S4.2. Thermodynamic data for the unit cell of β-**DADNE**, calculated at 300 K.

Fine Grid	ZPE / eV	F_{vib} / eV	C_v / J mol^{-1} K^{-1}
2	9.928	8.846	621.171
3	9.933	8.871	620.740
4	9.929	8.864	621.043
5	9.927	8.854	621.191
6	9.927	8.852	621.195
7	9.926	8.852	621.198

Table S4.3. Thermodynamic data for the unit cell of **trioxane**, calculated at 300 K.

Fine Grid	ZPE / eV	F_{vib} / eV	C_v / J mol^{-1} K^{-1}
2	5.301	5.035	177.243
3	5.296	5.021	177.597
4	5.298	5.028	177.432
5	5.297	5.025	177.495
6	5.297	5.023	177.524
7	5.297	5.024	177.496

Table S4.4. Thermodynamic data for the unit cell of **TATB**, calculated at 300 K.

Fine Grid	ZPE / eV	F_{vib} / eV	C_v / J mol^{-1} K^{-1}
2	8.480	7.768	494.813
3	8.462	7.692	495.936
4	8.461	7.683	496.046
5	8.461	7.686	496.031
6	8.460	7.683	496.062
7	8.460	7.682	496.082

Table S4.5. Thermodynamic data for the unit cell of **benzoquinone**, calculated at 300 K.

Fine Grid	ZPE / eV	F_{vib} / eV	C_v / J mol^{-1} K^{-1}
2	4.551	4.195	221.965
3	4.553	4.205	221.780
4	4.546	4.167	222.249
5	4.546	4.170	222.233
6	4.546	4.172	222.209
7	4.546	4.171	222.219

Table S4.6. Thermodynamic data for the unit cell of **paracetamol (Form I)**, calculated at 300 K.

Fine Grid	ZPE / eV	F_{vib} / eV	C_v / J mol^{-1} K^{-1}
2	17.110	16.014	699.571
3	17.118	16.058	699.114
4	17.124	16.085	698.761
5	17.122	16.078	698.897
6	17.121	16.074	698.955
7	17.121	16.075	698.946

Table S4.7. Thermodynamic data for the unit cell of **paracetamol (Form II)**, calculated at 300 K.

Fine Grid	ZPE / eV	F_{vib} / eV	C_v / J mol^{-1} K^{-1}
2	34.380	32.284	1415.614
3	34.356	32.150	1417.100
4	34.364	32.193	1416.764
5	34.364	32.193	1416.764
6	34.363	32.183	1416.679
7	34.362	32.174	1416.740

Table S4.8. Thermodynamic data for the unit cell of γ-**glycine**, calculated at 300 K.

Fine Grid	ZPE / eV	F_{vib} / eV	C_v / J mol^{-1} K^{-1}
2	6.604	6.263	245.390
3	6.607	6.270	245.096
4	6.605	6.265	245.215
5	6.606	6.267	245.173
6	6.606	6.268	245.158
7	6.606	6.268	245.161

Table S4.9. Thermodynamic data for the unit cell of α-**glycine**, calculated at 300 K.

Fine Grid	ZPE / eV	F_{vib} / eV	C_v / J mol^{-1} K^{-1}
2	8.775	8.254	337.972
3	8.778	8.262	337.724
4	8.776	8.257	337.847
5	8.776	8.255	337.887
6	8.776	8.255	337.893
7	8.776	8.255	337.892

Table S4.10. Thermodynamic data for the unit cell of **guanidinium nitrate**, calculated at 300 K.

Fine Grid	ZPE / eV	F_{vib} / eV	C_v / J mol^{-1} K^{-1}
2	2.812	2.603	126.919
3	2.802	2.590	128.442
4	2.802	2.592	128.421
5	2.802	2.592	128.418
6	2.802	2.592	128.426
7	2.802	2.592	128.426

Table S4.11. Thermodynamic data for the unit cell of **benzene**, calculated at 300 K.

Fine Grid	ZPE / eV	F_{vib} / eV	C_v / J mol^{-1} K^{-1}
2	10.787	10.218	368.361
3	10.788	10.221	368.291
4	10.788	10.221	368.290
5	10.788	10.221	368.291
6	10.788	10.221	368.292

Table S4.12. Thermodynamic data for the unit cell of **cubane**, calculated at 300 K.

Fine Grid	ZPE / eV	F_{vib} / eV	C_v / J mol^{-1} K^{-1}
2	10.730	10.360	323.745
3	10.730	10.360	323.745
4	10.730	10.360	323.745
5	10.730	10.360	323.745
6	10.730	10.360	323.745

S5 Local Vibrational Mode Analysis

We performed local vibrational mode analysis using as input the phonon frequencies and eigenvectors at the highest and lowest $G_{\mathrm{max}}^{\mathrm{fine}}$ grids for each OMC. A comparison of the local mode force constants that results form this analysis provides insight into the effect that $G_{\mathrm{max}}^{\mathrm{fine}}$ has on bonding analysis within this framework, Figures S5.1-S5.8. Generally we see only small changes in local mode force constants as $G_{\mathrm{max}}^{\mathrm{fine}}$ is increased. Notably, this change is only seen for systems whose phonon frequencies are also sensitive to $G_{\mathrm{max}}^{\mathrm{fine}}$.

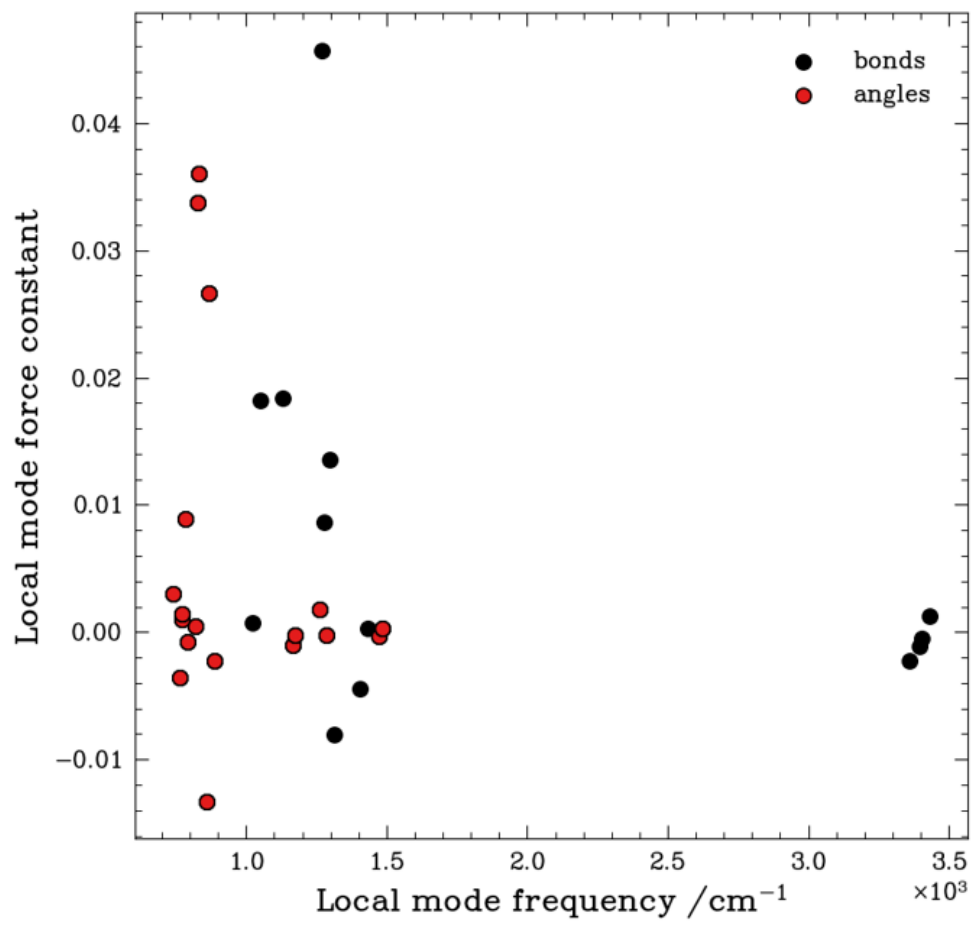

Fig. S5.1. Difference in local mode force constants for α-**DADNE** obtained from analysis performed on phonon spectra calculated using $G_{\mathrm{max}}^{\mathrm{fine}}$ with $S = 2$ and $S = 7$. Force constants have units mdyn Å^{-1} for bonds and mdyn Å rad^{-2} for angles.

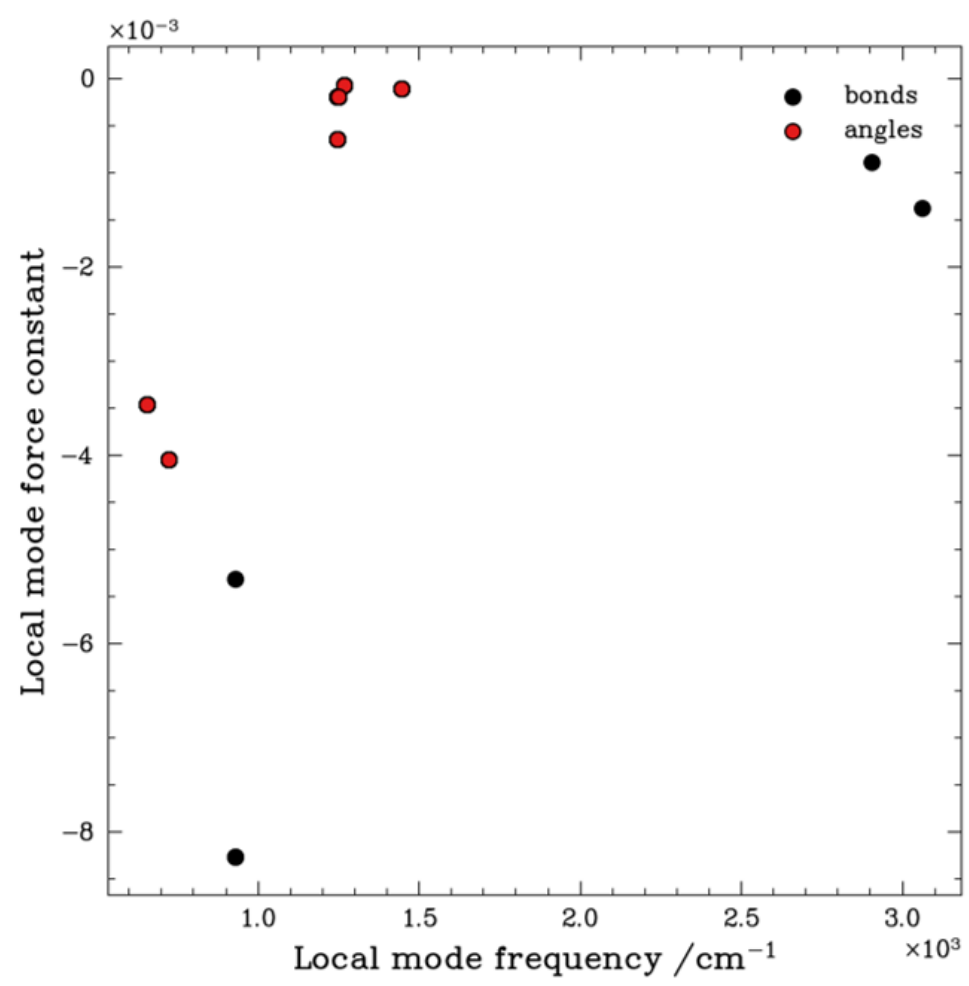

Fig. S5.2. Difference in local mode force constants for **trioxane** obtained from analysis performed on phonon spectra calculated using $G_{\mathrm{max}}^{\mathrm{fine}}$ with $S = 2$ and $S = 7$. Force constants have units mdyn Å^{-1} for bonds and mdyn Å rad^{-2} for angles.

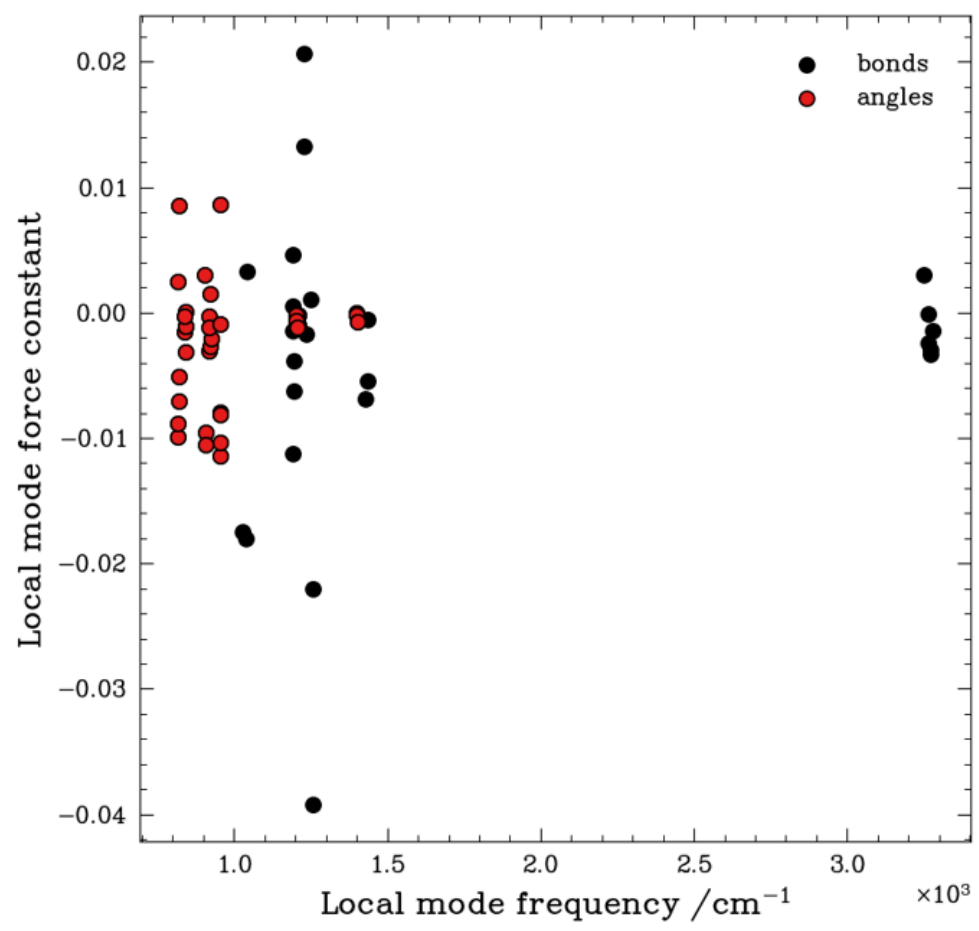

Fig. S5.3. Difference in local mode force constants for **TATB** obtained from analysis performed on phonon spectra calculated using $G_{\max}^{\text{fine}}$ with $S = 2$ and $S = 7$. Force constants have units mdyn Å^{-1} for bonds and mdyn Å rad^{-2} for angles.

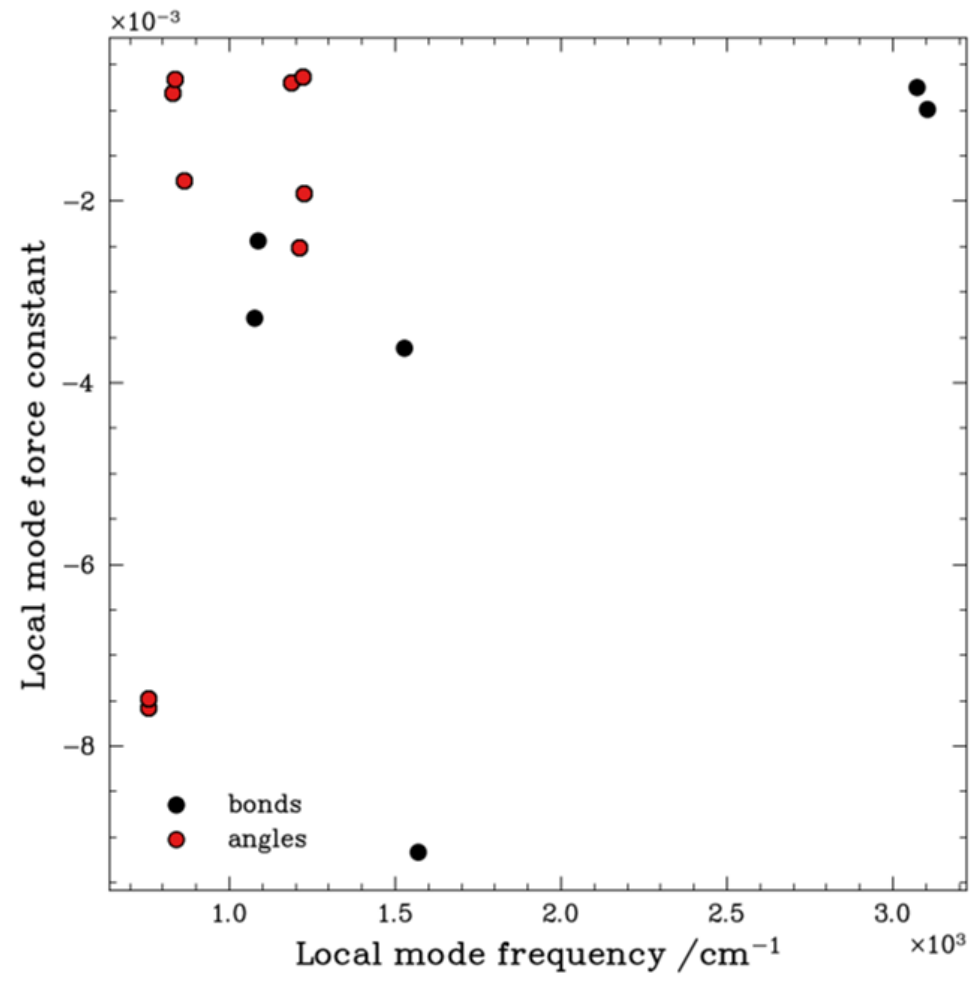

Fig. S5.4. Difference in local mode force constants for **benzoquinone** obtained from analysis performed on phonon spectra calculated using $G_{\max}^{\text{fine}}$ with $S = 2$ and $S = 7$. Force constants have units mdyn Å^{-1} for bonds and mdyn Å rad^{-2} for angles.

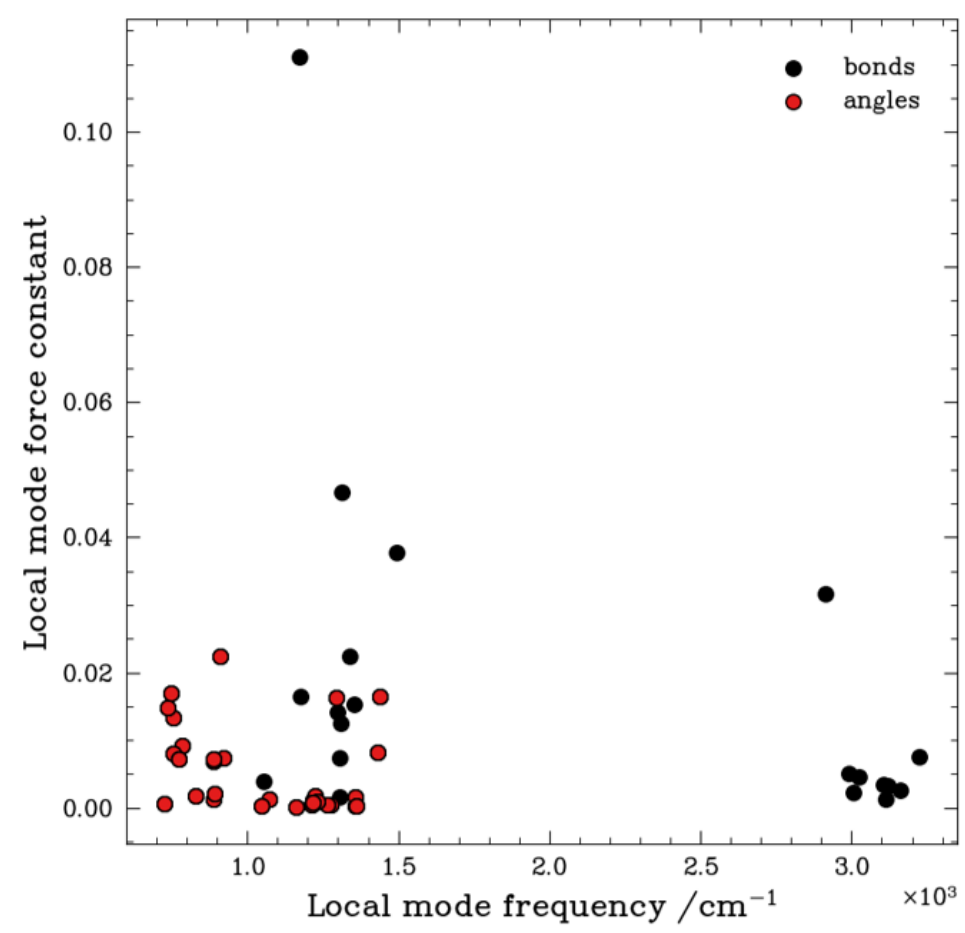

Fig. S5.5. Difference in local mode force constants for **paracetamol (Form I)** obtained from analysis performed on phonon spectra calculated using $G_{\max}^{\text{fine}}$ with $S = 2$ and $S = 7$. Force constants have units mdyn Å^{-1} for bonds and mdyn Å rad^{-2} for angles.

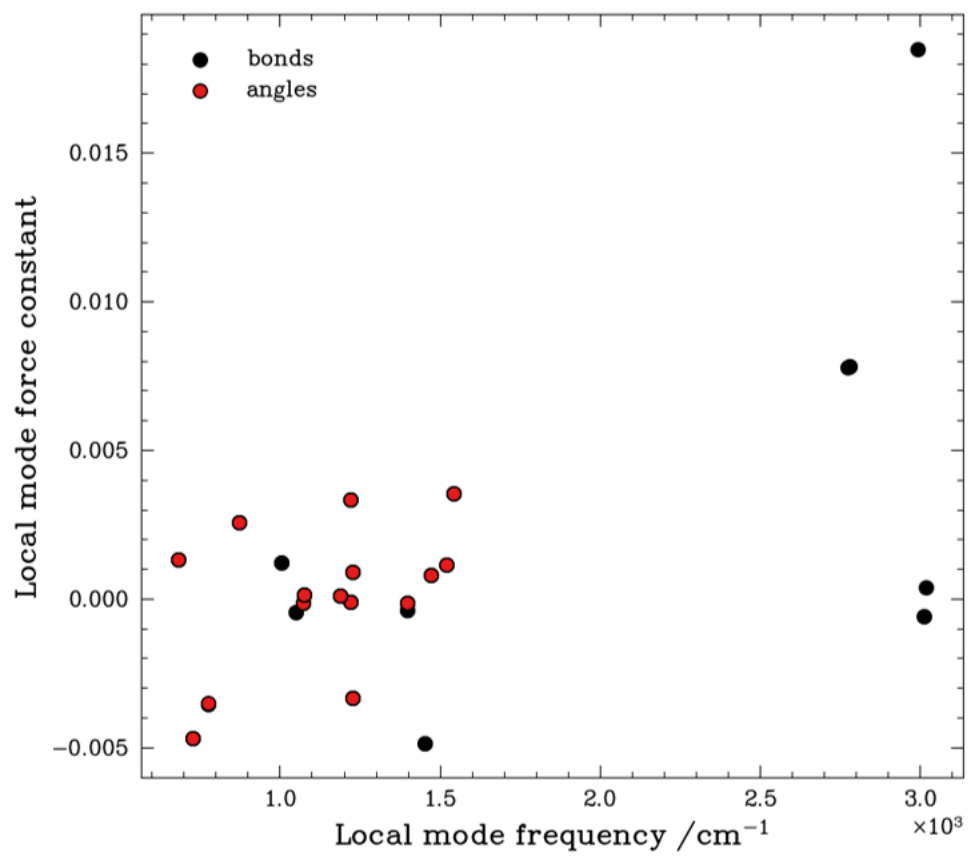

Fig. S5.6. Difference in local mode force constants for γ-**glycine** obtained from analysis performed on phonon spectra calculated using $G_{\text{max}}^{\text{fine}}$ with $S = 2$ and $S = 7$. Force constants have units mdyn Å^{-1} for bonds and mdyn Å rad^{-2} for angles.

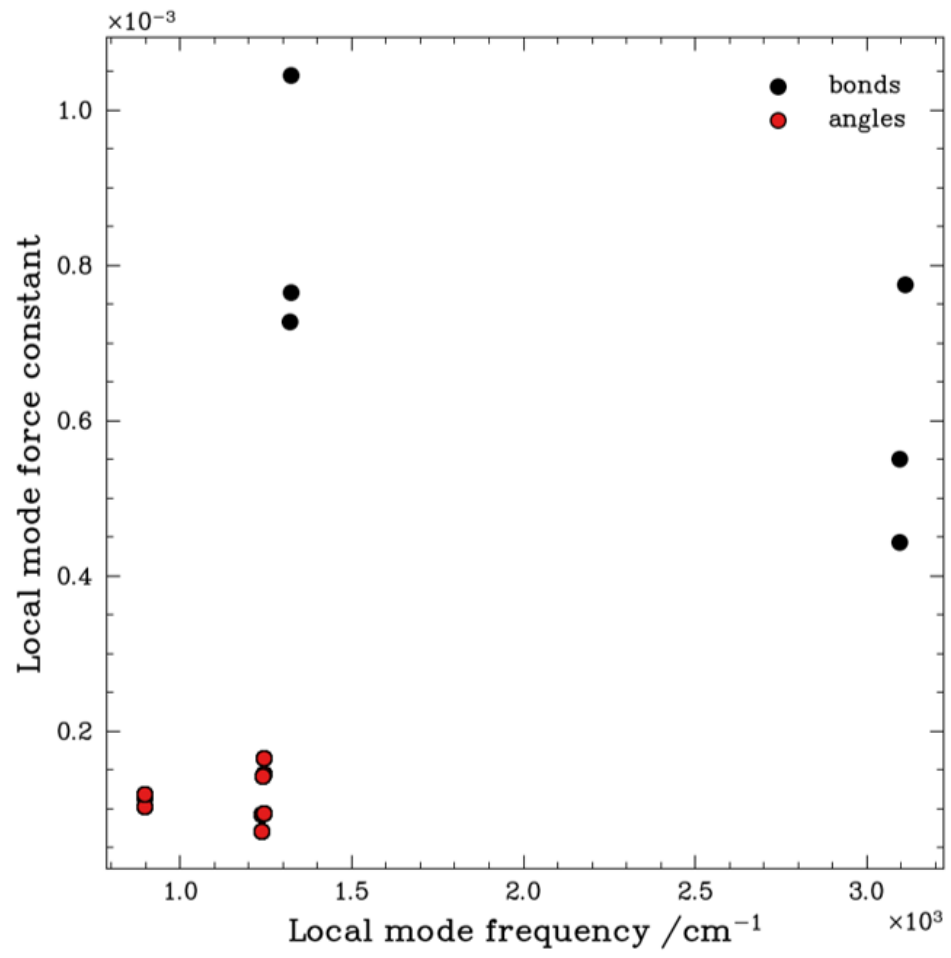

Fig. S5.7. Difference in local mode force constants for **benzene** obtained from analysis performed on phonon spectra calculated using $G_{\text{max}}^{\text{fine}}$ with $S = 2$ and $S = 7$. Force constants have units mdyn Å^{-1} for bonds and mdyn Å rad^{-2} for angles.

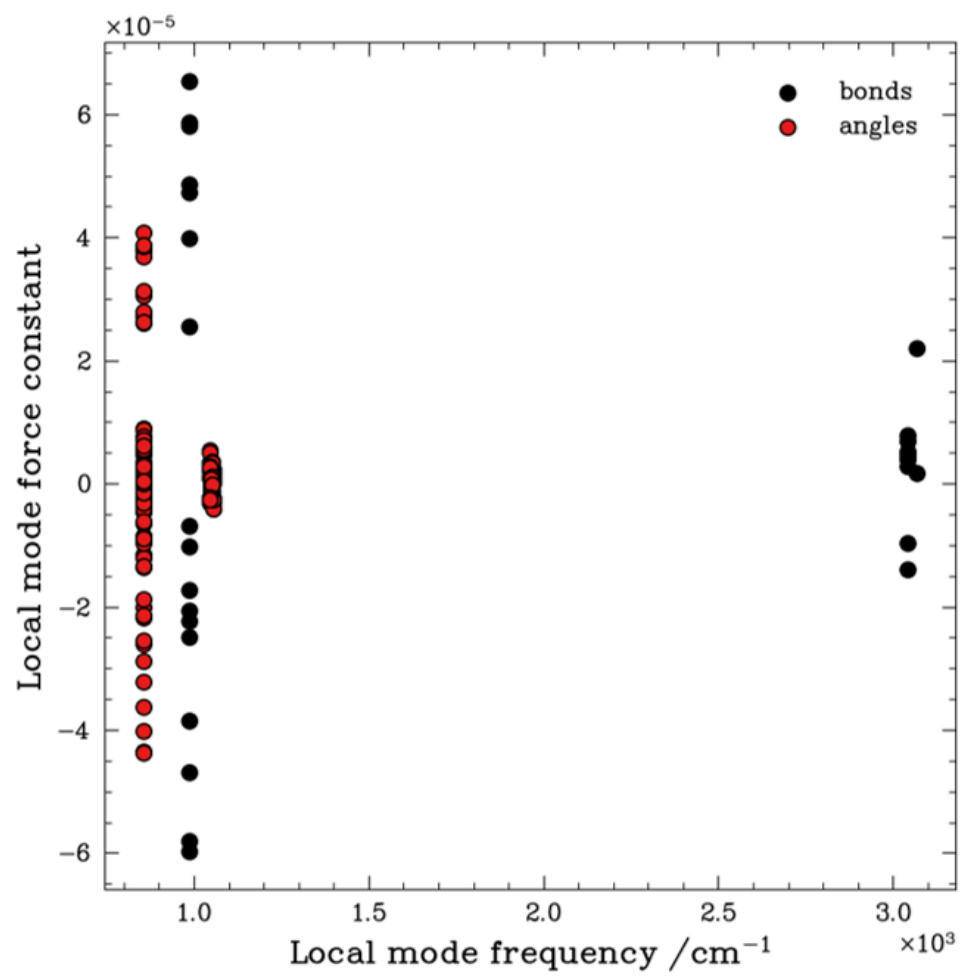

Fig. S5.8. Difference in local mode force constants for **cubane** obtained from analysis performed on phonon spectra calculated using $G_{\text{max}}^{\text{fine}}$ with $S = 2$ and $S = 7$. Force constants have units mdyn Å^{-1} for bonds and mdyn Å rad^{-2} for angles.

S6 Converging Phonon Dispersion Behaviour

S6.1 Phonon dispersion parameters and thermodynamic behaviour

Each of our phonon dispersion curves were calculated using $G^{std}_{max} = 2G_{max}$ and a G^{fine}_{max} defined using a scaling factor S (see Eqn S1.1) that ensured all Γ-point frequencies were converged, as identified in Section S2.

Table S6.1. Thermodynamic data for Glycine calculated on increasingly fine Monkhorst-Pack q-point grids using phonons calculated using electron densities represented on a Fourier grid with scaling $S = 6$, calculated at 300 K.

MP Grid (q-point Spacing / $Å^{-1}$) (# q-points)	ZPE / eV	F_{vib} / eV	C_v / J mol^{-1} K^{-1}
111 (above 0.182) (1)	7.036	6.226	248.505
112 (0.145–0.181) (2)	7.086	6.205	264.014
222 (0.091–0.144) (4)	7.102	6.204	268.910
223 (0.073–0.090) (4)	7.102	6.203	268.911
333 (0.061–0.072) (8)	7.102	6.204	268.912
334 (0.049–0.060) (11)	7.102	6.204	268.914
444 (0.046–0.048) (14)	7.102	6.204	268.913
445 (0.037–0.045) (16)	7.102	6.204	268.913

Table S6.2. Thermodynamic data for Benzoquinone calculated on increasingly fine Monkhorst-Pack q-point grids using phonons calculated using electron densities represented on a Fourier grid with scaling $S = 6$, calculated at 300 K.

MP Grid (q-point Spacing / $Å^{-1}$) (# q-points)	ZPE / eV	F_{vib} / eV	C_v / J mol^{-1} K^{-1}
111 (above 0.174) (1)	4.550	4.232	207.134
211 (0.153–0.173) (2)	4.548	4.167	216.877
222 (0.087–0.150) (8)	4.554	4.035	246.404
322 (0.077–0.086) (8)	4.554	4.036	246.405
333 (0.058–0.076) (10)	4.554	4.036	246.401
433 (0.051–0.057) (14)	4.554	4.036	246.400
444 (0.044–0.050) (30)	4.554	4.035	246.404
544 (0.039–0.043) (33)	4.554	4.035	246.405
555 (0.035–0.038) (39)	4.554	4.035	246.405

Table S6.3. Thermodynamic data for Benzene calculated on increasingly fine Monkhorst-Pack q-point grids using phonons calculated using electron densities represented on a Fourier grid with scaling $S = 3$, calculated at 300 K.

MP Grid (q-point Spacing / $Å^{-1}$) (# q-points)	ZPE / eV	F_{vib} / eV	C_v / J mol^{-1} K^{-1}
111 (above 0.155) (1)	10.785	10.072	384.079
112 (0.141–0.154) (2)	10.785	10.073	384.759
212 (0.111-0.140) (4)	10.791	10.068	392.921
222 (0.078-0.010) (8)	10.795	10.092	392.918
223 (0.071–0.077) (8)	10.792	10.082	393.079
323 (0.056–0.070) (8)	10.792	10.078	393.132
333 (0.052–0.055) (8)	10.792	10.078	393.144
334 (0.047–0.051) (12)	10.792	10.078	393.135
434 (0.039–0.046) (18)	10.792	10.080	392.997

Table S6.4. Thermodynamic data for Cubane calculated on increasingly fine Monkhorst-Pack q-point grids using phonons calculated using electron densities represented on a Fourier grid with scaling $S = 2$, calculated at 300 K.

MP Grid (q-point Spacing / Å^{-1}) (# q-points)	ZPE / eV	F_{vib} / eV	C_v / J mol^{-1} K^{-1}
111 (above 0.169) (1)	10.757	10.324	347.064
221 (0.092–0,168) (4)	10.739	10.247	347.858
222 (0.085–0.091) (8)	10.739	10.245	347.861
332 (0.057–0.084) (10)	10.739	10.246	347.866
442 (0.046–0.056) (20)	10.739	10.245	347.870
443 (0.043–0.045) (26)	10.739	10.244	347.876
553 (0.034–0.042) (38)	10.738	10.244	347.878

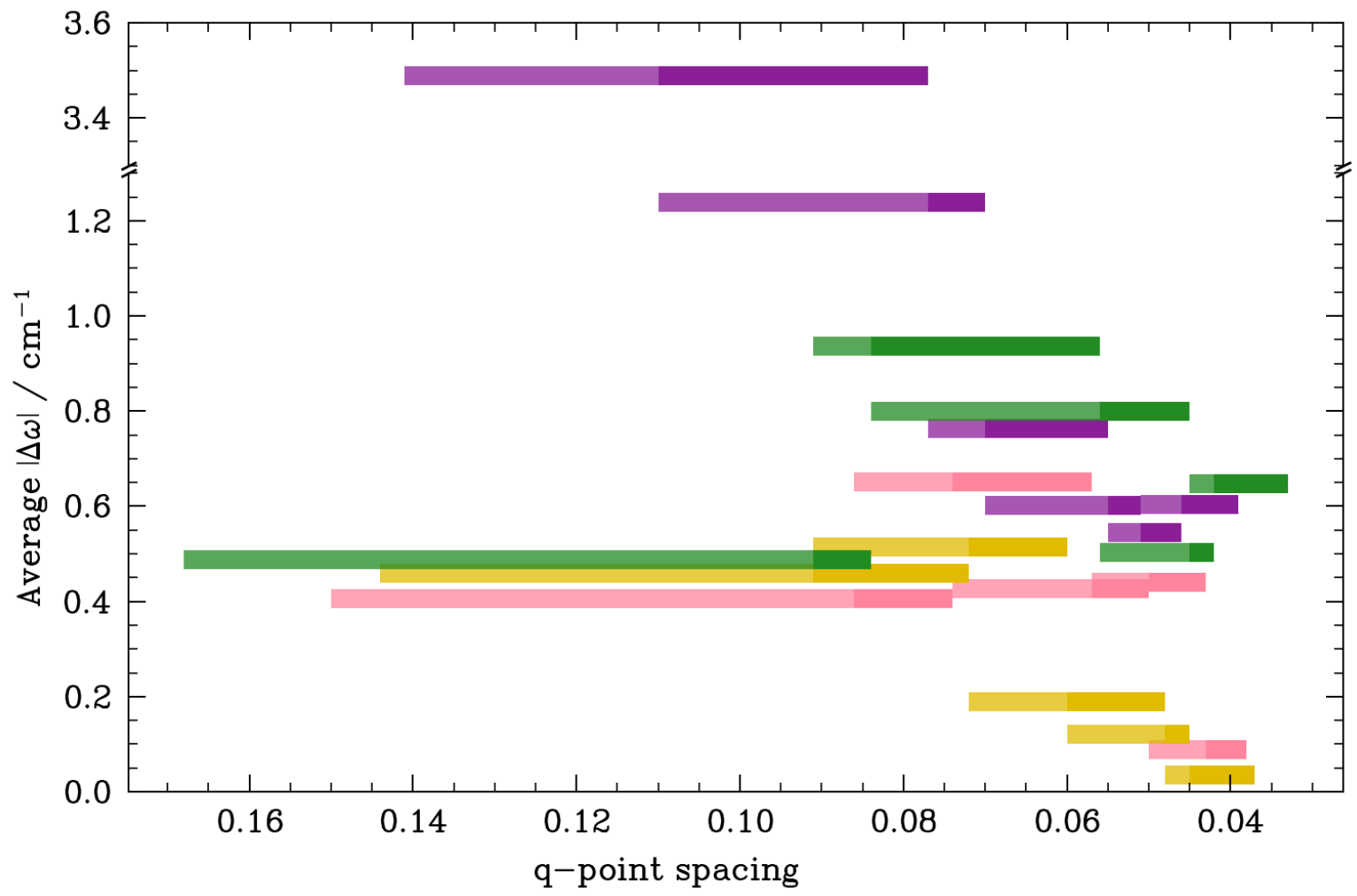

Fig. S6.1. Average absolute change in the phonon frequencies across the phonon dispersion as the q-point spacing is reduced for γ-**glycine** (yellow), **benzoquinone** (pink), **benzene** (violet) and **cubane** (green).

S6.2 Phonon dispersion calculated with different q-point grids.

The phonon dispersion curves were plotted for γ-glycine (Section S6.2.1), benzoquinone (Section S6.2.2), benzene (Section S6.2.3) and cubane S6.2.4) by interpolating dynamical matrices from increasingly densely sampled q-point grids, as detailed in Section S6.1. We additionally plot phonon dispersion difference with increasing q-point grid densities to demonstrate the how the phonon bands change with increasing q-point sampling density.

S6.2.1 Phonon dispersion for γ-glycine calculated on different explicit q-point grids using $G_{\text{max}}^{\text{fine}}$ setting V.

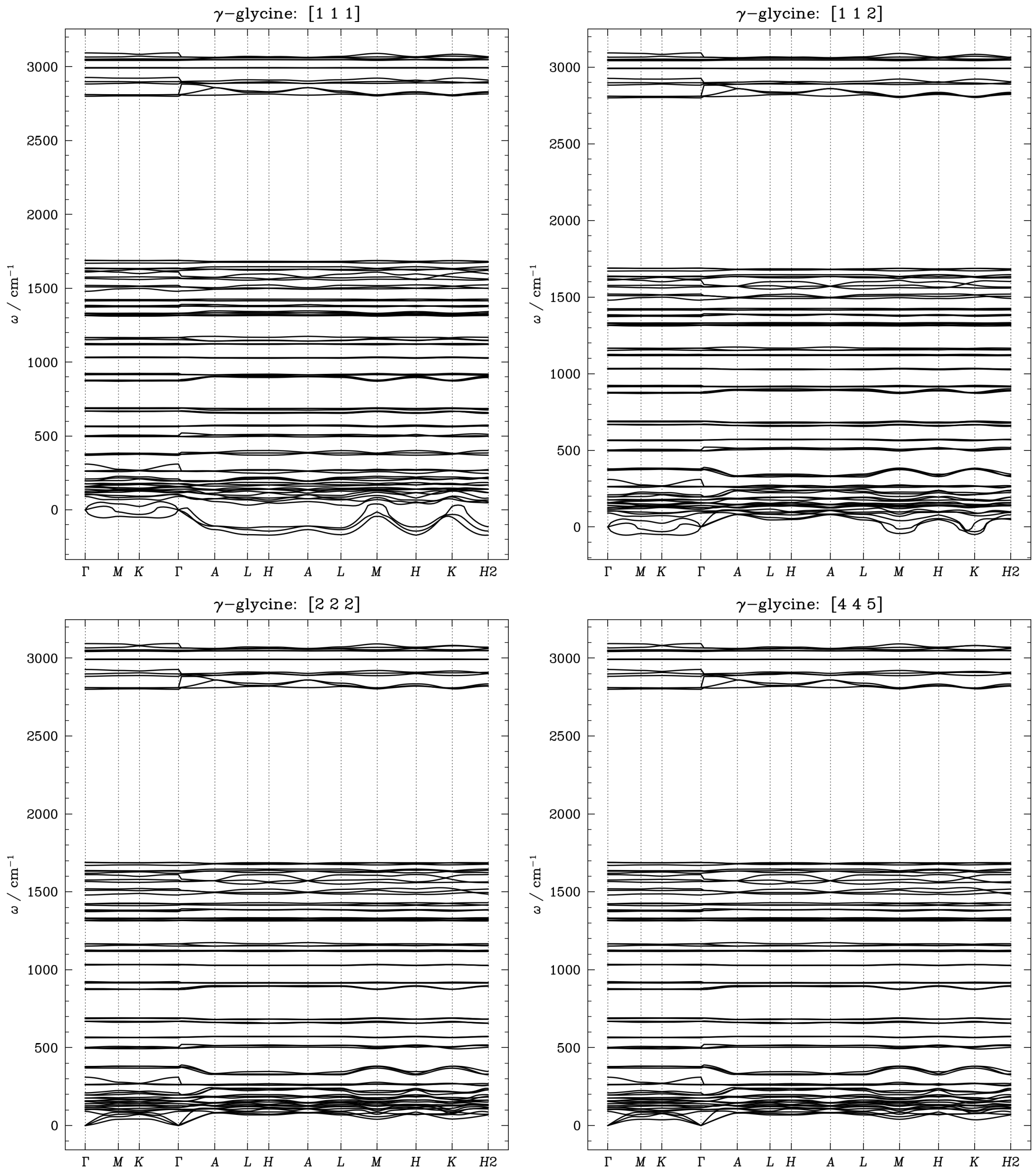

Fig. S6.2. Phonon dispersion for γ-**glycine** at explicit q-point grids of [1 1 1], [1 1 2], [2 2 2] and [4 4 5].

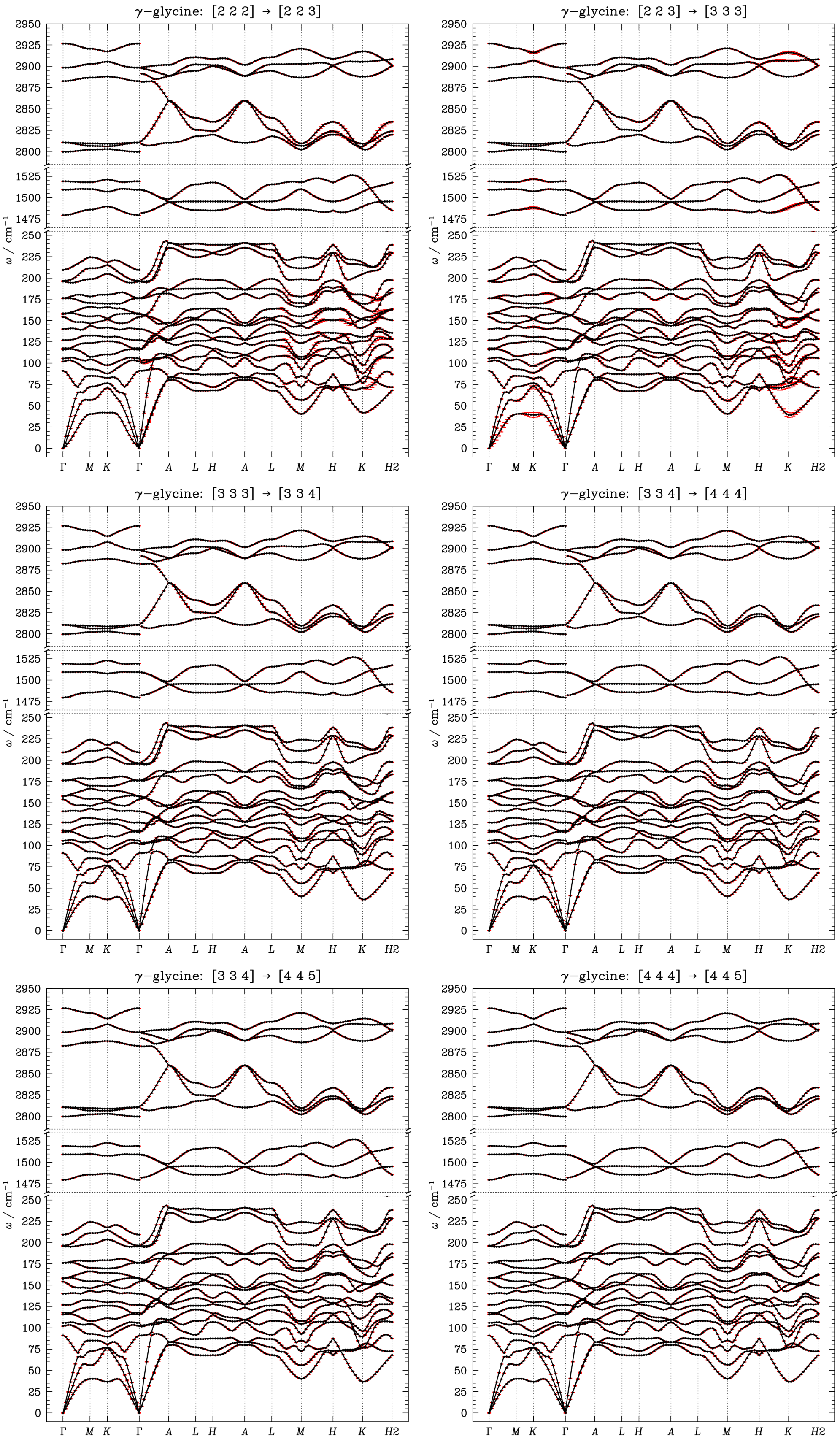

Fig. S6.3. Phonon dispersion difference with increasing q-point grid for γ-**glycine**. The average frequency for each phonon mode is plotted as black dots at each q-point, and the maximum and minimum values are shown as red error bars.

S6.2.2 Phonon dispersion for benzoquinone calculated on different explicit q-point grids using $G_{\max}^{\text{fine}}$ setting V.

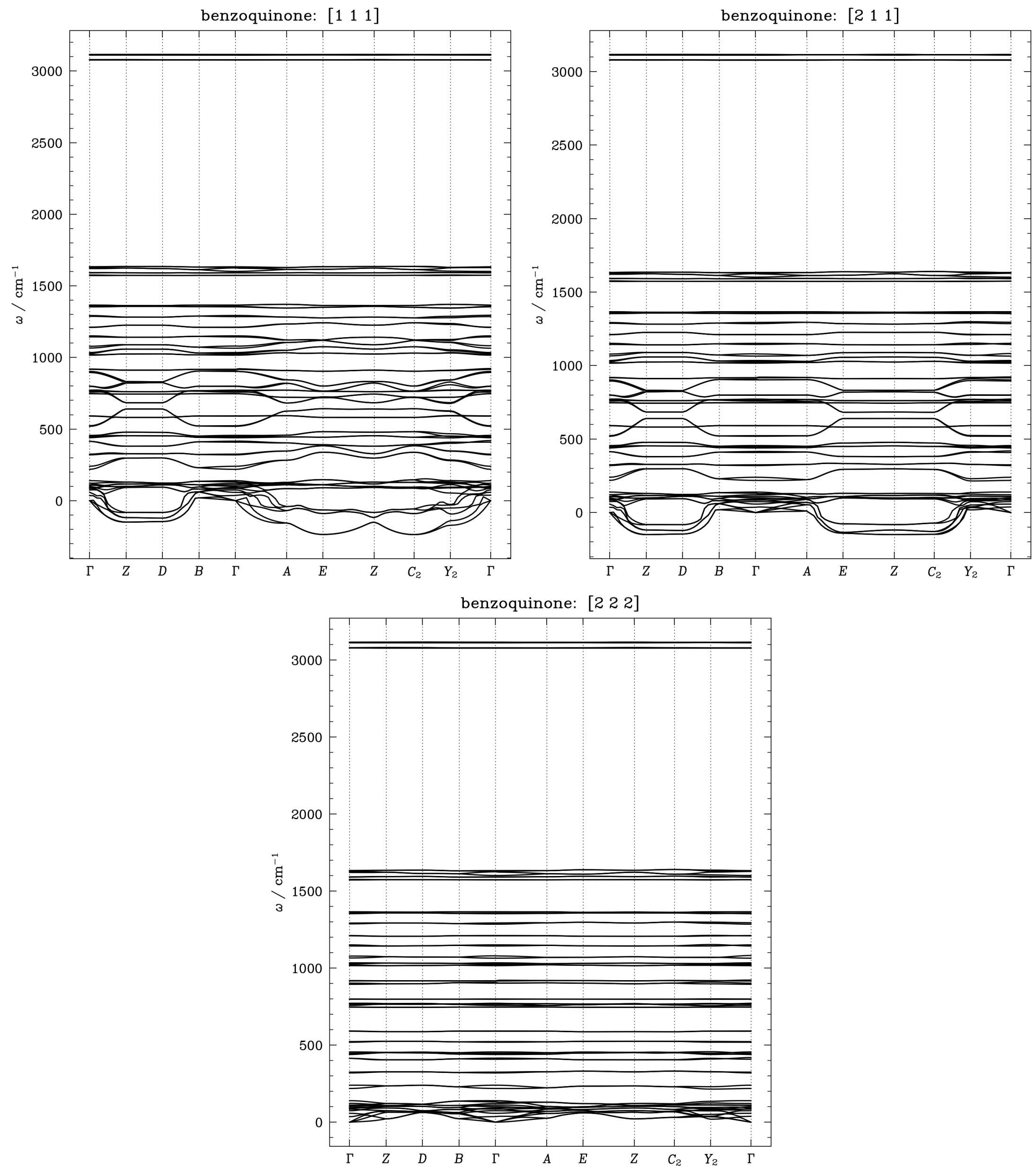

Fig. S6.4. Phonon dispersion for **benzoquinone** at explicit q-point grids of [1 1 1], [2 1 1], [2 2 2].

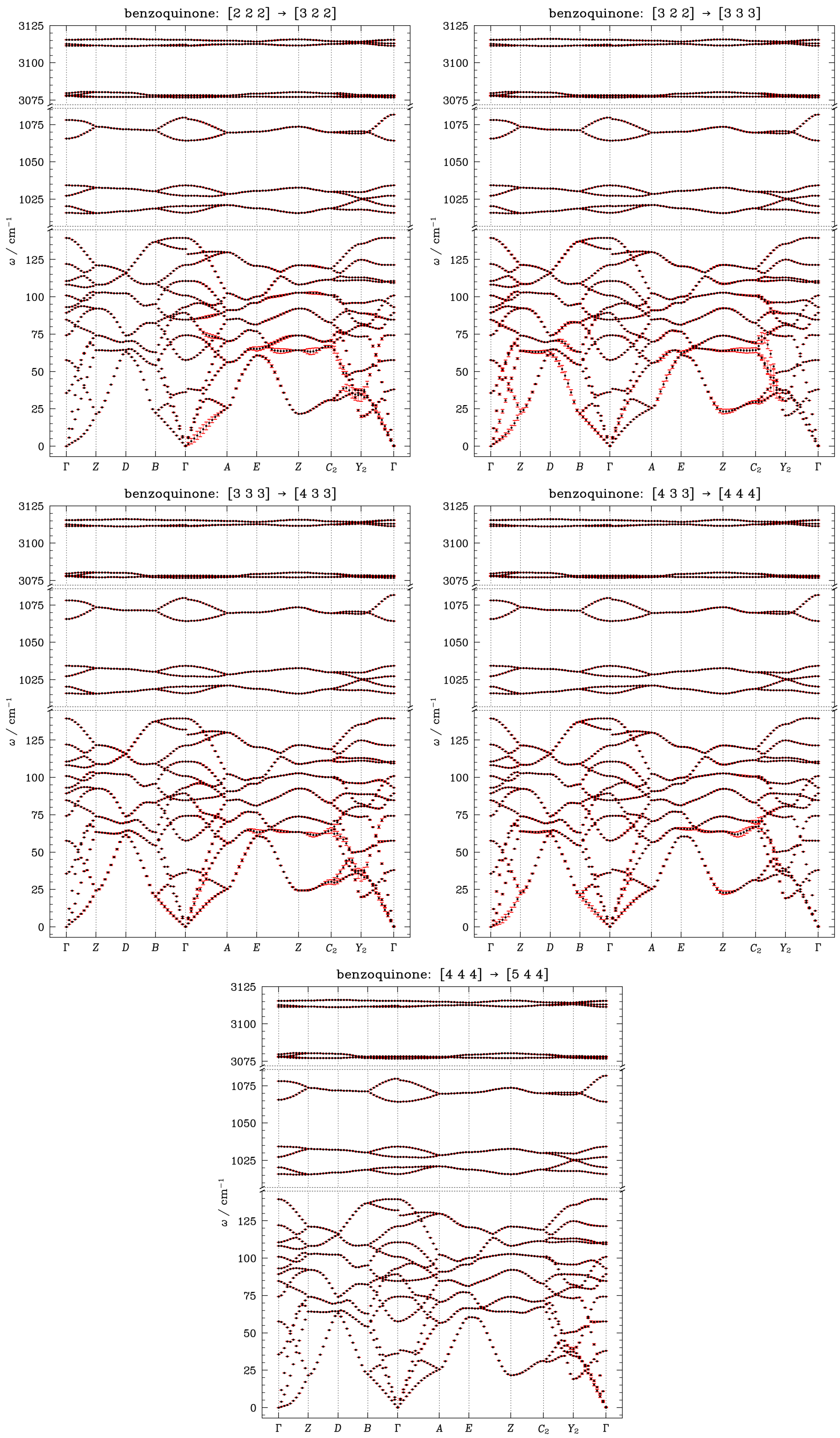

Fig. S6.5. Phonon dispersion difference with increasing q-point grid for **benzoquinone**. The average frequency for each phonon mode is plotted as black dots at each q-point, and the maximum and minimum values are shown as red error bars.

S6.2.3 Phonon dispersion for benzene calculated using different explicit q-point grids using $G_{\mathrm{max}}^{\mathrm{fine}}$ setting II.

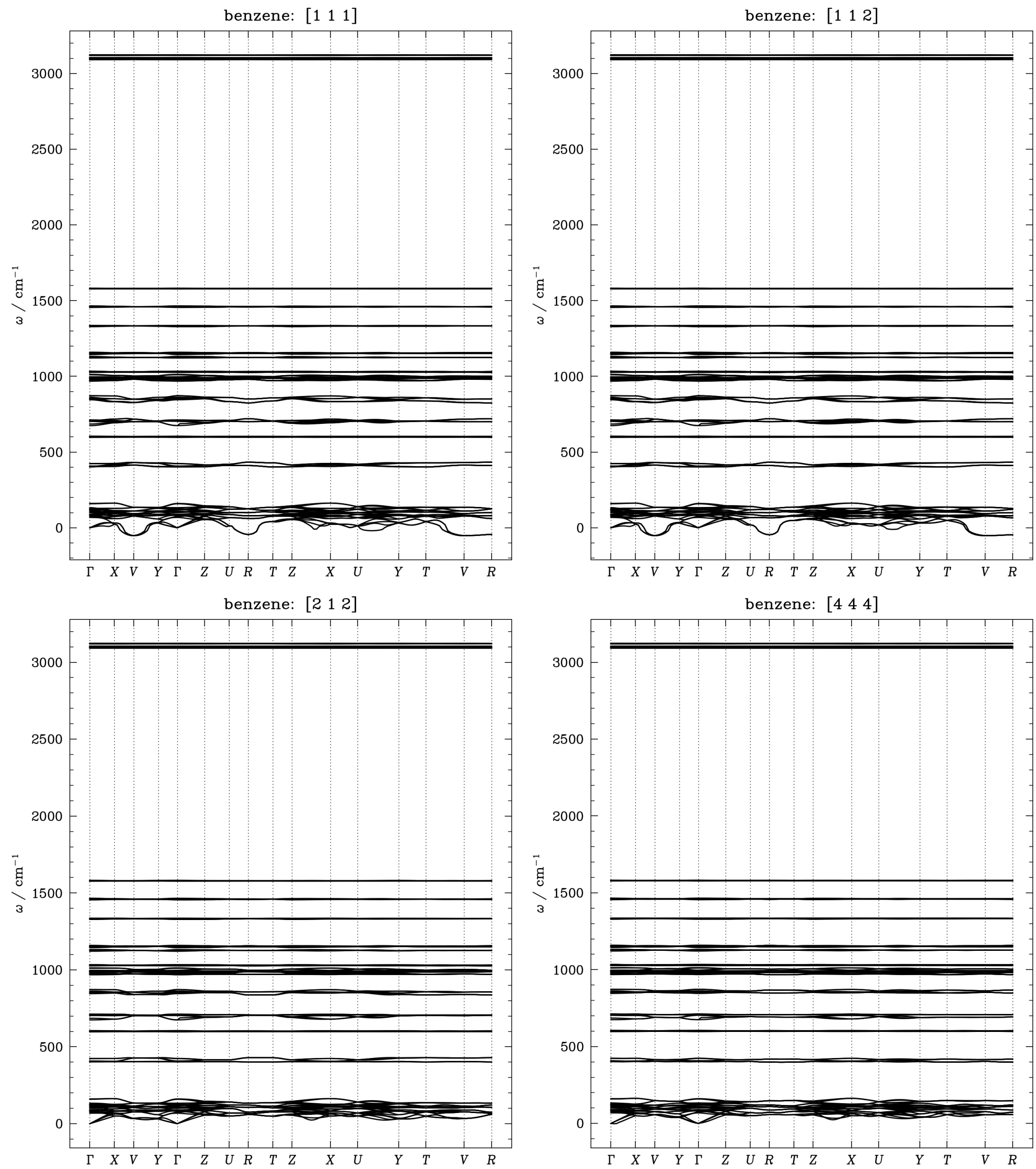

Fig. S6.6. Phonon dispersion for **benzene** at explicit q-point grids of [1 1 1], [1 1 2], [2 1 2], and [4 4 4].

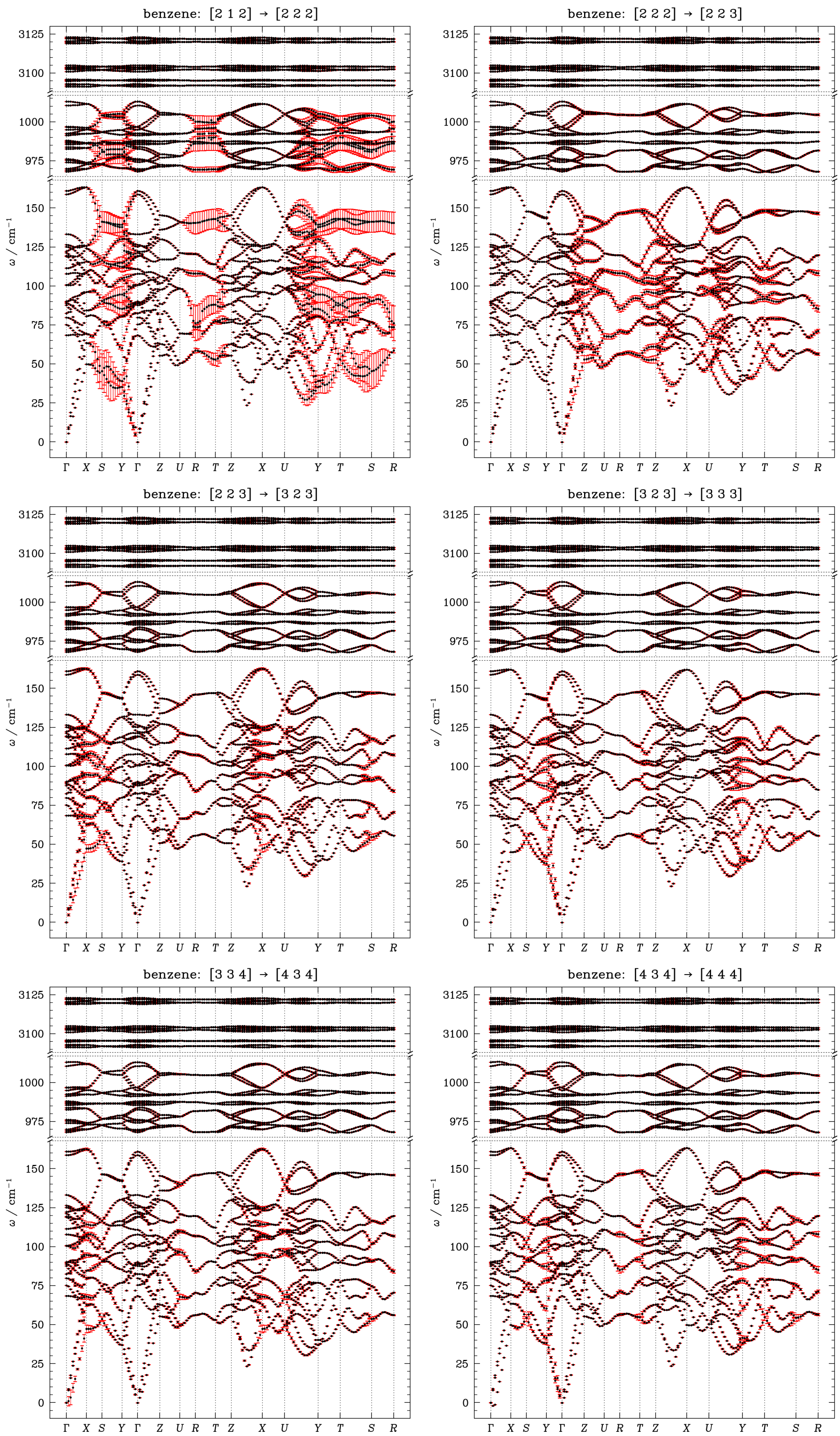

Fig. S6.7. Phonon dispersion difference with increasing q-point grid for **benzene**. The average frequency for each phonon mode is plotted as black dots at each q-point, and the maximum and minimum values are shown as red error bars.

S6.2.4 Phonon dispersion for cubane calculated on different explicit q-point grids using $G_{\mathrm{max}}^{\mathrm{fine}}$ setting I.

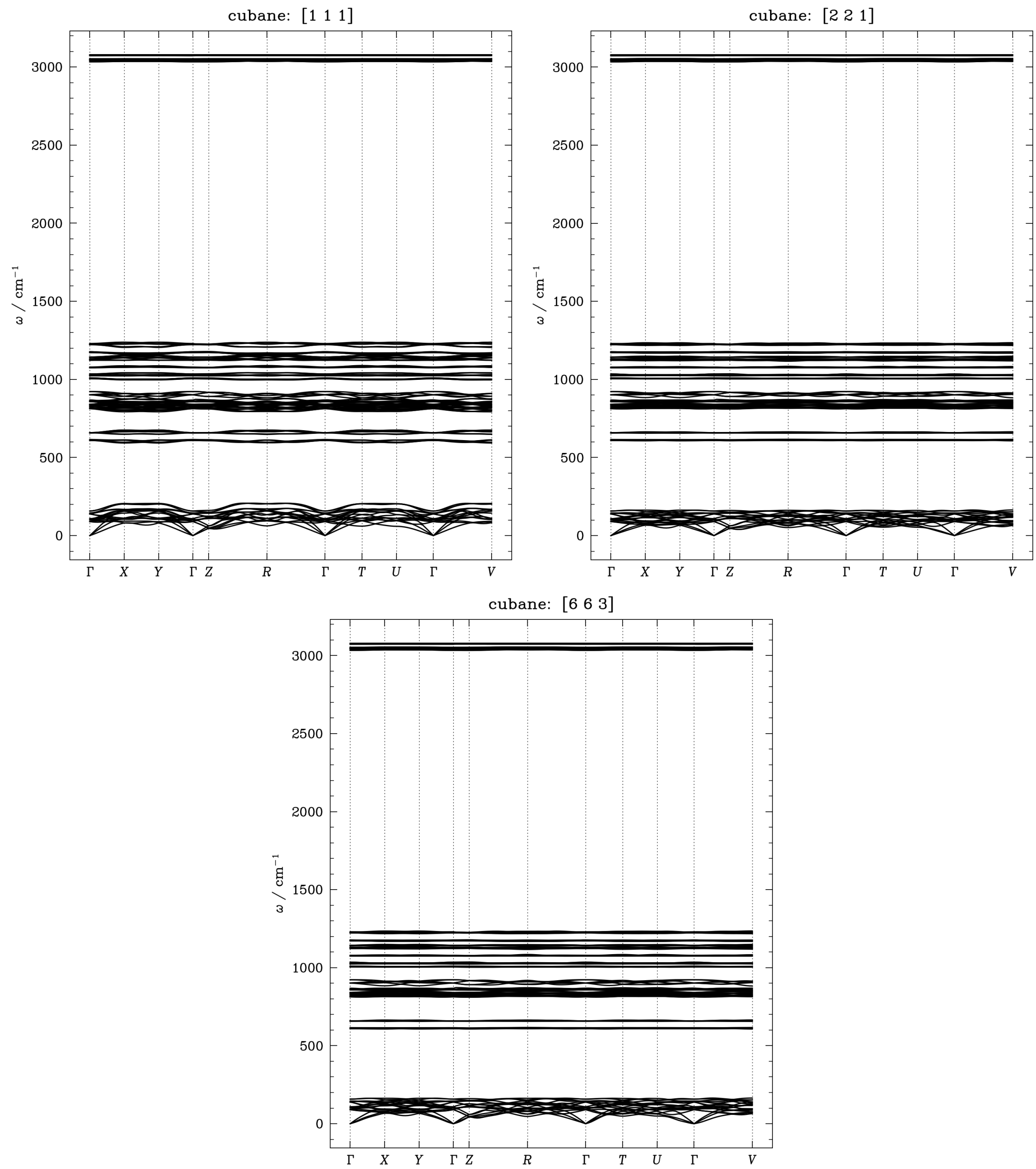

Fig. S6.8. Phonon dispersion for **cubane** at explicit q-point grids of [1 1 1], [2 2 1], and [6 6 3].

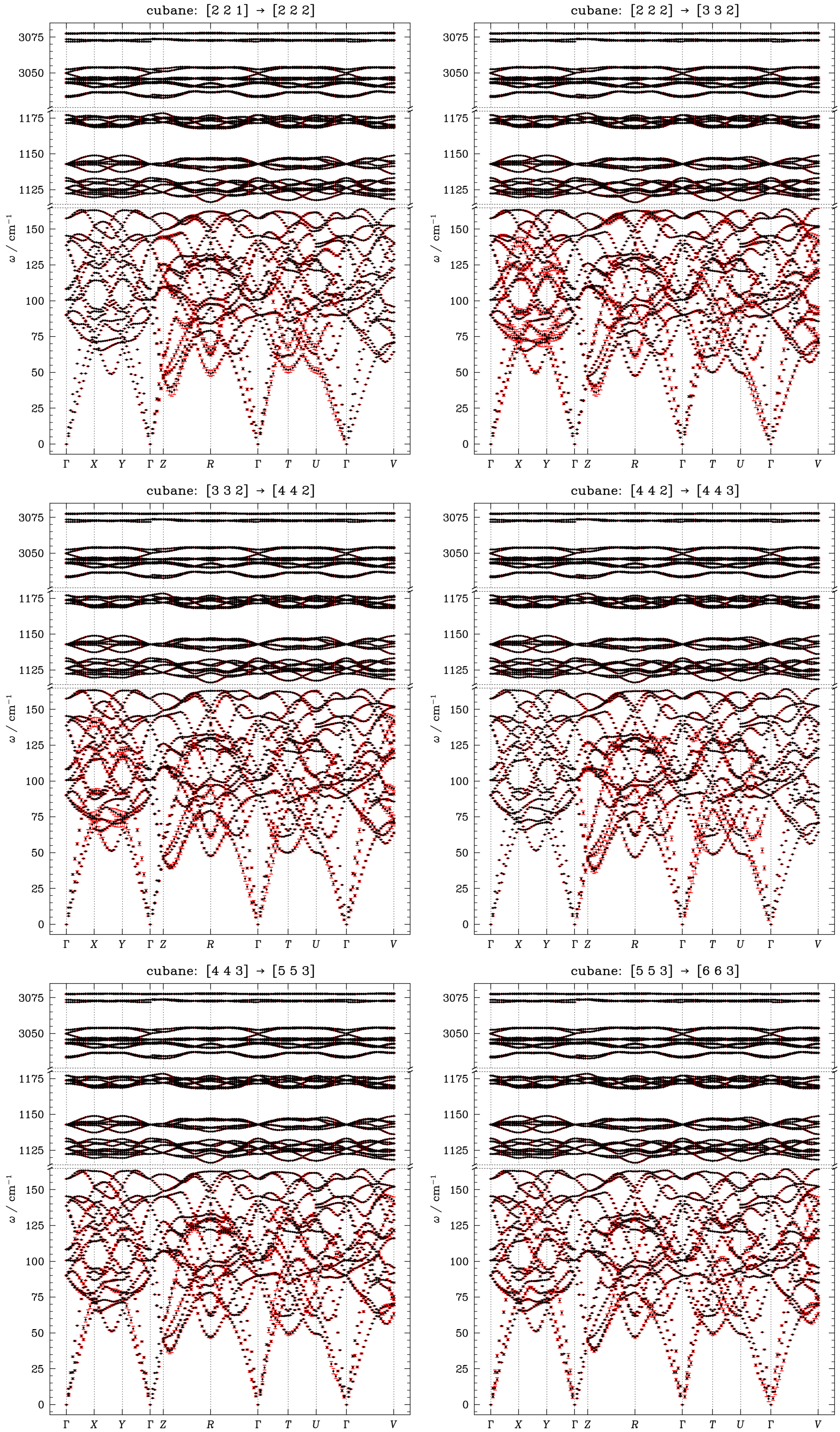

Fig. S6.9. Phonon dispersion difference with increasing q-point grid for **cubane**. The average frequency for each phonon mode is plotted as black dots at each q-point, and the maximum and minimum values are shown as red error bars.

References

[1] J. Evers, T. M. Klapötke, P. Mayer, G. Oehlinger and J. Welch, *Inorg. Chem.*, 2006, **45**, 4996–5007.

[2] V. Busetti, A. Del Pra and M. Mammi, *Acta Cryst.*, 1969, **B25**, 1191–1194.

[3] Z. Chua, C. G. Gianopoulos, B. Zarychta, E. A. Zhurova, V. V. Zhurov and A. A. Pinkerton, *Cryst. Growth Des.*, 2017, **17**, 5200–5207.

[4] M. Bolte, G. Margraf and W. Lerner, *CSD Communication*, 2002.

[5] D. Druzhbin, T. Drebushchak, V. Minkov and E. Boldyreva, *J. Str. Chem.*, 2015, **56**, 317–323.

[6] G. Nichols and C. S. Frampton, *Journal of Pharmaceutical Sciences*, 1998, **87**, 684–693.

[7] A. Kvick, W. M. Canning, T. F. Koetzle and G. J. B. Williams, *Acta Cryst. B: Str. Cryst. Cryst. Chem.*, 1980, **36**, 115–120.

[8] J. Almlöf, Å. Kvick and J. O. Thomas, *The Journal of Chemical Physics*, 1973, **59**, 3901–3906.

[9] A. Katrusiak and M. Szafrański, *Acta Crystallographica Section C*, 1994, **50**, 1161–1163.

[10] M. Woińska, S. Grabowsky, P. M. Dominiak, K. Woźniak and D. Jayatilaka, *Sci. Adv.*, 2016, **2**, e1600192.

[11] R. J. Doedens, P. E. Eaton and E. B. Fleischer, *Eur. J. Chem.*, 2017, **2017**, 2627–2630.

[12] A. A. L. Michalchuk, S. Rudić, C. R. Pulham and C. A. Morrison, *Chem. Commun.*, 2021, **57**, 11213–11216.

[13] A. A. L. Michalchuk, M. Trestman, S. Rudić, P. Portius, P. T. Fincham, C. R. Pulham and C. A. Morrison, *J. Mater. Chem. A*, 2019, **7**, 19539–19553.

[14] B. S. Hudson, *Inelastic Neutron Scattering spectrum of 1,3,5-Trioxane, (CH2O)3, measured on the TOSCA instrument.*, http://dx.doi.org/10.5286/edata/668, 2017, Accessed Nov 5, 2024.

[15] S. A. Rivera, D. G. Allis and B. S. Hudson, *Cryst. Growth Des.*, 2008, **8**, 3905 – 3907.

[16] H. Jobic, *Stud. Surf. Sci. Catal.*, 1996, **764**, 559 – 566.
